# The KAT8-CDK1 K33 acetylation axis drives doxorubicin resistance by suppressing ferroptosis and apoptosis in breast cancer

**DOI:** 10.1038/s41419-026-09162-6

**Published:** 2026-08-03

**Authors:** Qingzhi Zhao, Qixian Zou, Jinmeng Chu, Yizhen Wang, Tiantian Xu, Haoqing Dou, Chengyu Cai, Na Zhang, Fei Wang, Yin Gao, Yong Cai, Bing Liang, Jingji Jin

**Affiliations:** 1 School of Life Sciences, Jilin University, Changchun, China; 2 School of Nursing, Jilin University, Changchun City, China

**Keywords:** Breast cancer, Epigenetics

## Abstract

Doxorubicin (Dox) resistance severely limits therapeutic efficacy in breast cancer, yet the epigenetic mechanisms linking cell-cycle control to therapy-induced cell death remain unclear. Here, we identify the histone acetyltransferase KAT8 as a critical driver of Dox resistance. Transcriptomic analysis of neoadjuvant cohorts revealed elevated KAT8 expression in residual disease (RD) tumors, which was validated in an independent cohort of 95 patients. High KAT8 levels correlated with poor therapeutic response. Mechanistically, KAT8 directly acetylated CDK1 at lysine 33 (K33) in Dox-resistant MCF-7/ADR and MDA-MB-231/ADR cells. K33 acetylation sustained CDK1 phosphorylation at T14, Y15, and T161, maintaining kinase activity under chemotherapeutic stress. Disruption of KAT8, either by genetic silencing or pharmacological inhibition with MG149, reduced CDK1 activation, increased mitochondrial depolarization and oxidative stress, and restored Dox sensitivity. Functionally, KAT8-dependent CDK1 K33 acetylation suppressed both apoptosis and ferroptosis, two principal Dox-induced cell death pathways. Re-expression of wild-type CDK1, but not the acetylation-deficient K33R mutant, rescued chemoresistance. In vivo, MG149 co-treatment or expression of CDK1-K33R significantly enhanced Dox-mediated tumor suppression in xenograft models without overt toxicity. Together, these findings establish KAT8-dependent CDK1 K33 acetylation as a key epigenetic mechanism sustaining anthracycline resistance and suggest that targeting the KAT8-CDK1 axis may provide a therapeutic strategy to overcome refractory breast cancer.

**Figure:**
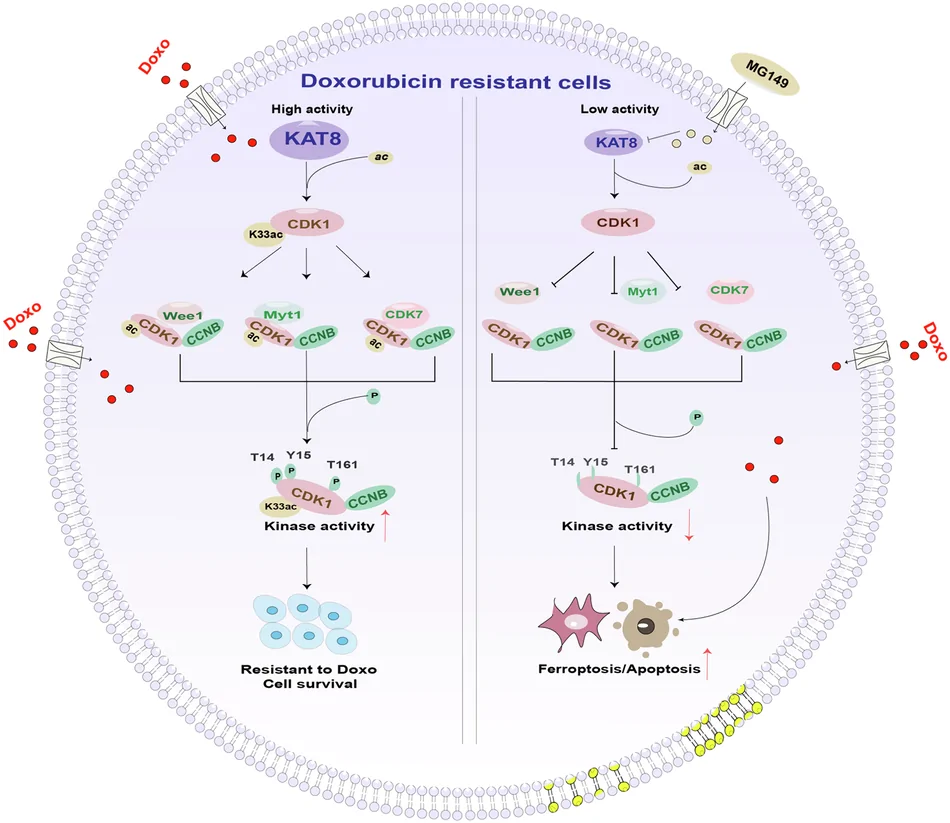

## Introduction

Breast cancer remains the most frequently diagnosed malignancy among women worldwide. Although doxorubicin (Dox) is a cornerstone of systemic therapy [1], the development of acquired resistance substantially limits its clinical efficacy [2]. Elucidating the molecular mechanisms underlying Dox resistance is therefore essential for improving therapeutic outcomes.

Post-translational modifications (PTMs), particularly lysine acetylation, have emerged as critical regulators of tumor drug resistance [3–6]. The acetyltransferase KAT8 (MOF) modulates chromatin dynamics, DNA damage repair, and mitochondrial homeostasis through acetylation of histone and non-histone substrates [7–10]. Increasing evidence implicates KAT8 in chemoresistance across multiple cancer types, and elevated KAT8 expression has been associated with poor chemotherapy response in breast cancer [11, 12], suggesting a potential role in anthracycline resistance.

Cyclin-dependent kinase 1 (CDK1) is a central regulator of cell cycle progression and DNA damage response [13, 14]. Its activity is primarily controlled by phosphorylation at T14, Y15, and T161, while additional PTMs, including acetylation, further modulate its function [15–17]. Acetylation of CDK1 at lysine 33 (K33) has been reported to alter kinase activity; however, whether K33 acetylation directly affects phosphorylation dynamics and downstream cell death signaling remains unclear.

Dox exerts cytotoxic effects by inducing DNA damage, oxidative stress, and mitochondrial dysfunction, thereby activating regulated cell death programs, including apoptosis and ferroptosis [18, 19]. Restoration of ferroptotic sensitivity has recently emerged as a promising strategy for overcoming chemoresistance.

In this study, we identify KAT8 as a key epigenetic regulator of Dox resistance in breast cancer. We demonstrate that KAT8 directly acetylates CDK1 at K33, thereby modulating its phosphorylation status and attenuating apoptotic and ferroptotic cell death. Disruption of the KAT8–CDK1 axis restores Dox sensitivity in resistant models, providing a mechanistic rationale for targeting anthracycline resistance.

## Materials and methods

Additional methods are described in the Supplemental Methods.

### Ethics statement and breast cancer specimens

Human breast cancer specimens (*n* = 95) were obtained from the Department of Biobank, Division of Clinical Research, The First Hospital of Jilin University (Approval Number: 2023-395). Patients were categorized according to neoadjuvant treatment history and pathological response into three groups: no doxorubicin treatment (No-Dox, *n* = 40), residual disease (RD, *n* = 49), and pathological complete response (pCR, *n* = 6). All specimens were subjected to immunohistochemical staining for KAT8 and H4K16ac. The association between KAT8 and H4K16ac expression was evaluated using the H-score method followed by Spearman’s rank correlation analysis. Fresh tumor tissues were snap-frozen in liquid nitrogen and stored at −80 °C until use. Clinical data, including tumor stage and pathological diagnosis, were retrieved from medical records. For RNA extraction, 10 mg of tissue was homogenized in 400 μL TRIzol reagent. For protein analysis, 10 mg of tissue was lysed in 200 μL RIPA buffer.

### Public transcriptomic data analysis

Publicly available raw transcriptomic datasets were obtained from the Gene Expression Omnibus (GEO; http://www.ncbi.nlm.nih.gov/geo/). KAT8 expression in breast cancer tissues was analyzed using the GEO datasets GSE25065 and GSE34138. Survival analysis was conducted using the GEPIA platform.

### Cell culture

HEK293T, HepG2, MCF-7, and MDA-MB-231 cells were obtained from the Cell Bank of the Chinese Academy of Sciences. MCF-7/ADR and MDA-MB-231/ADR cells were purchased from iCell and IMMOCELL, respectively. All cells were cultured in DMEM supplemented with 10% fetal bovine serum and 1% penicillin–streptomycin at 37 °C in a humidified atmosphere containing 5% CO₂. To maintain the drug-resistant phenotype, MCF-7/ADR and MDA-MB-231/ADR cells were routinely maintained in medium supplemented with 0.4 µg/mL doxorubicin. Cell line authentication was performed by short tandem repeat (STR) profiling within the last three years, and all experiments were conducted using mycoplasma-free cells.

### Patient-derived organoids and xenograft assays

Patient-derived breast cancer organoids were established from freshly resected tumor specimens using a Matrigel-based three-dimensional culture system, as previously described [20]. Organoids were treated with DMSO, doxorubicin, MG149 or their combination. Organoid number and size were quantified from bright-field images using ImageJ software.

### Animal experiments

Five-week-old female BALB/c nude mice were purchased from SPF (Beijing) Biotechnology Co., Ltd (Beijing, China). Mice were maintained under specific pathogen-free conditions at 23 ± 2 °C with a 12 h light/dark cycle and ad libitum access to food and water. All animal procedures were approved by the Institutional Animal Care and Use Committee (IACUC) of Jilin University (Approval No. IACUC Issue No. (2025) Shen YNPZSY No 0608). Sample sizes were determined using the resource equation method. Mice were randomly assigned, based on body weight, to the following groups: control, Dox, MG149, Dox + MG149, Dox + CDK1-WT, and Dox + CDK1-K33R. MCF-7/ADR cells stably expressing CDK1-WT or CDK1-K33R were expanded to logarithmic growth phase and subcutaneously injected into the right axilla (1 × 10^7^ cells per mouse). When tumor volumes reached ~80–100 mm³, mice received intraperitoneal injections of saline, Dox and/or MG149 on days 1, 4, 7, 11, 14, 17, 21, and 24, according to group allocation. Tumor growth was monitored throughout the study. Tumor length and width were measured every three days using calipers, and tumor volume (mm^3^) was calculated as (length × width²)/2. At the study endpoint, mice were euthanized and tumors were harvested for Western blot analysis, hematoxylin and eosin (H&E) staining, and immunohistochemistry (IHC). Serum samples were collected simultaneously for biochemical analysis.

### Immunohistochemistry quantification

IHC staining was quantified using the H-score method. Staining intensity was graded as 0 (negative), 1 (weak), 2 (moderate), or 3 (strong). The percentage of tumor cells at each intensity level was determined, and the H-score was calculated according to the formula: H-score = (1 × % weakly stained cells) + (2 × % moderately stained cells) + (3 × % strongly stained cells). The final H-score ranged from 0 to 300. At least five representative high-power fields were analyzed per section. All slides were independently evaluated by two investigators blinded to clinical information, and the mean value was used for subsequent statistical analysis.

### Statistical analysis

Data are presented as mean ± SD. Differences between two groups were evaluated using Student’s *t*-test, and comparisons among multiple groups were analyzed by one-way ANOVA with post-hoc testing. Spearman’s rank correlation was used for H-score correlation; chi-square or Fisher’s exact test was used for high/low categorical association. *P* < 0.05 was considered statistically significant.

## Results

### High KAT8 expression correlates with doxorubicin resistance in breast cancer

**Fig. 1:**
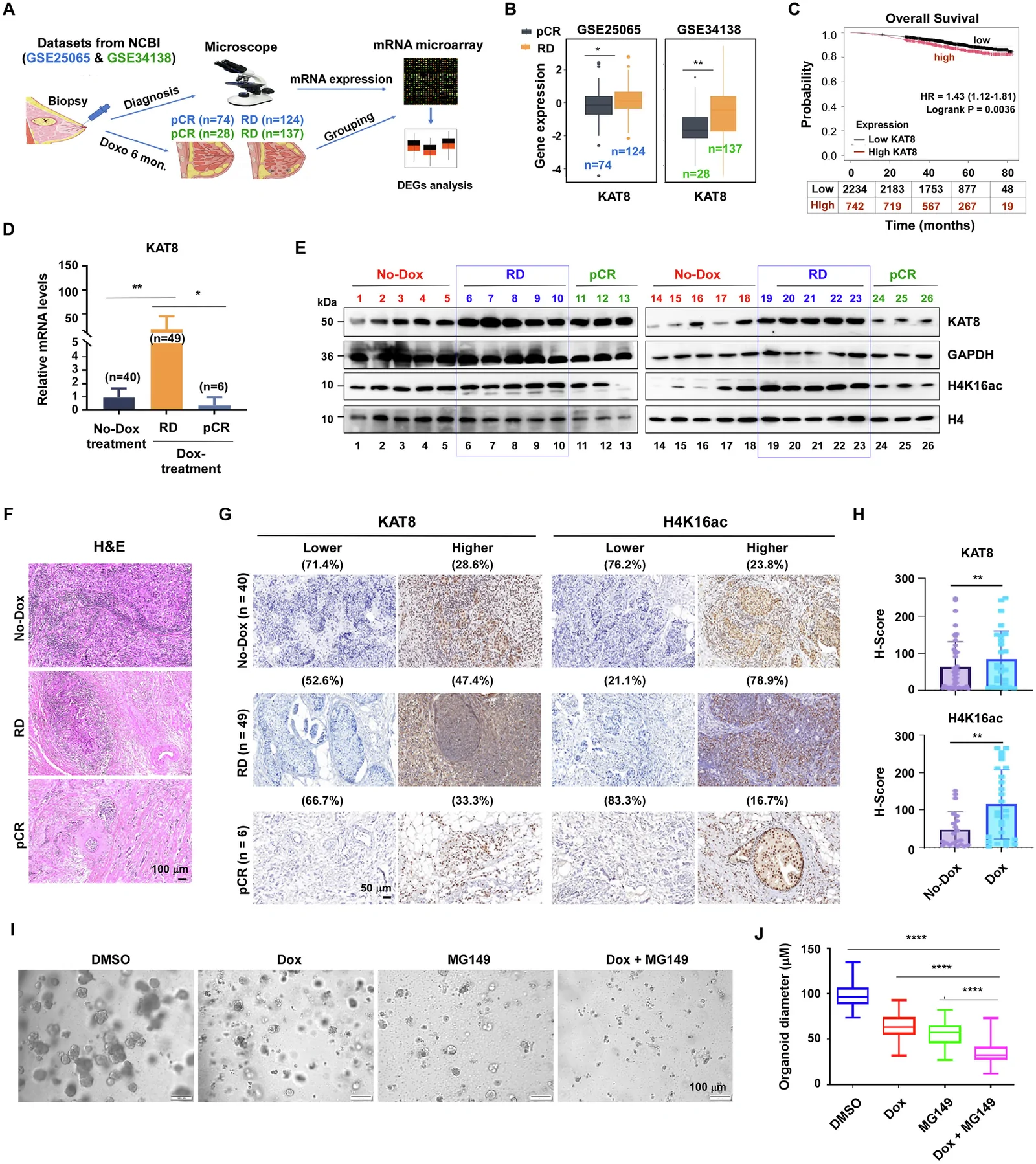
Elevated KAT8 expression in breast cancer patients with residual disease (RD). **A** Workflow for transcriptomic analysis of GEO datasets GSE25065 and GSE34138, stratified by pathological response following Dox-containing neoadjuvant therapy. **B** KAT8 mRNA expression in pCR and RD groups in GSE25065 and GSE34138. Sample sizes: GSE25065, pCR (*n* = 74), RD (*n* = 124); GSE34138, pCR (*n* = 28), RD (*n* = 137). **C** Kaplan–Meier overall survival analysis stratified by KAT8 expression level. **D** qRT-PCR analysis of KAT8 mRNA in clinical breast cancer tissues from No-Dox (*n* = 40), RD (*n* = 49), and pCR (*n* = 6) groups. **E** Immunoblot analysis of KAT8 and H4K16ac in representative clinical samples. **F** Representative H&E staining of No-Dox, RD, and pCR tumor tissues. **G** Representative IHC staining images and proportional classification of KAT8 and H4K16ac expression in the IHC cohort. **H** H-score quantification of KAT8 and H4K16ac expression. **I** Representative images of patient-derived organoids treated with DMSO, Dox, MG149, or Dox + MG149. **J** Quantification of organoid diameter under indicated treatments. Data are presented as mean ± SD unless otherwise specified. *P < 0.05, **P < 0.01, ***P < 0.001, ****P < 0.0001.

To investigate the clinical relevance of KAT8 in Dox resistance, we first analyzed two neoadjuvant breast cancer cohorts (GSE25065 and GSE34138). Patients were stratified according to pathological response following Dox-containing therapy, with residual disease (RD) defined as a resistant phenotype and pathological complete response (pCR) as a sensitive phenotype (Fig. 1A). In both datasets, KAT8 mRNA levels were significantly higher in RD tumors than in pCR tumors (Fig. 1B). Kaplan-Meier analysis further demonstrated that elevated KAT8 expression was associated with shorter overall survival (HR = 1.43, 95% CI 1.12-1.81, log-rank *P* = 0.0036; Fig. 1C). These findings were validated in an independent clinical cohort comprising No-Dox patients (*n* = 40), RD patients (*n* = 49), and pCR patients (*n* = 6). Consistently, KAT8 mRNA expression was markedly increased in RD tumors compared with No-Dox and pCR samples (Fig. 1D). Immunoblot analysis of representative specimens revealed elevated KAT8 and H4K16ac protein levels in RD tumors, whereas pCR tumors exhibited lower expression (Fig. 1E). H&E staining confirmed the histopathological characteristics of No-Dox, RD, and pCR tissues (Fig. 1F). In a subset of samples subjected to IHC, the proportions of KAT8-high and H4K16ac-high tumors were increased in RD tissues relative to No-Dox or pCR tissues (Fig. 1G). Quantitative H-score analysis further demonstrated enhanced KAT8 and H4K16ac staining in Dox-exposed tumors (Figs. 1H, S1A). Moreover, in patient-derived organoid models, pharmacological inhibition of KAT8 with MG149 suppressed organoid growth and potentiated Dox-induced growth inhibition (Fig. 1I, J). To assess the association between KAT8 expression and molecular subtypes, breast cancer samples were stratified by Ki-67, ER, HER2, and PR status, and KAT8 mRNA levels were compared between No-Dox and Dox-treated groups. KAT8 expression was significantly higher in the Dox-treated group among ER-positive (*P* = 0.022), HER2-negative (*P* = 0.007), and PR-positive (*P* = 0.002) tumors. No significant differences were observed after Ki-67 stratification or within ER-negative, HER2-positive, and PR-negative subgroups (Table S1). Collectively, these data demonstrated that KAT8 is upregulated in Dox-resistant breast cancer and that pharmacological targeting of KAT8 restores drug sensitivity in clinically relevant organoid models.

### KAT8 mediates doxorubicin resistance independently of P-gp

**Fig. 2:**
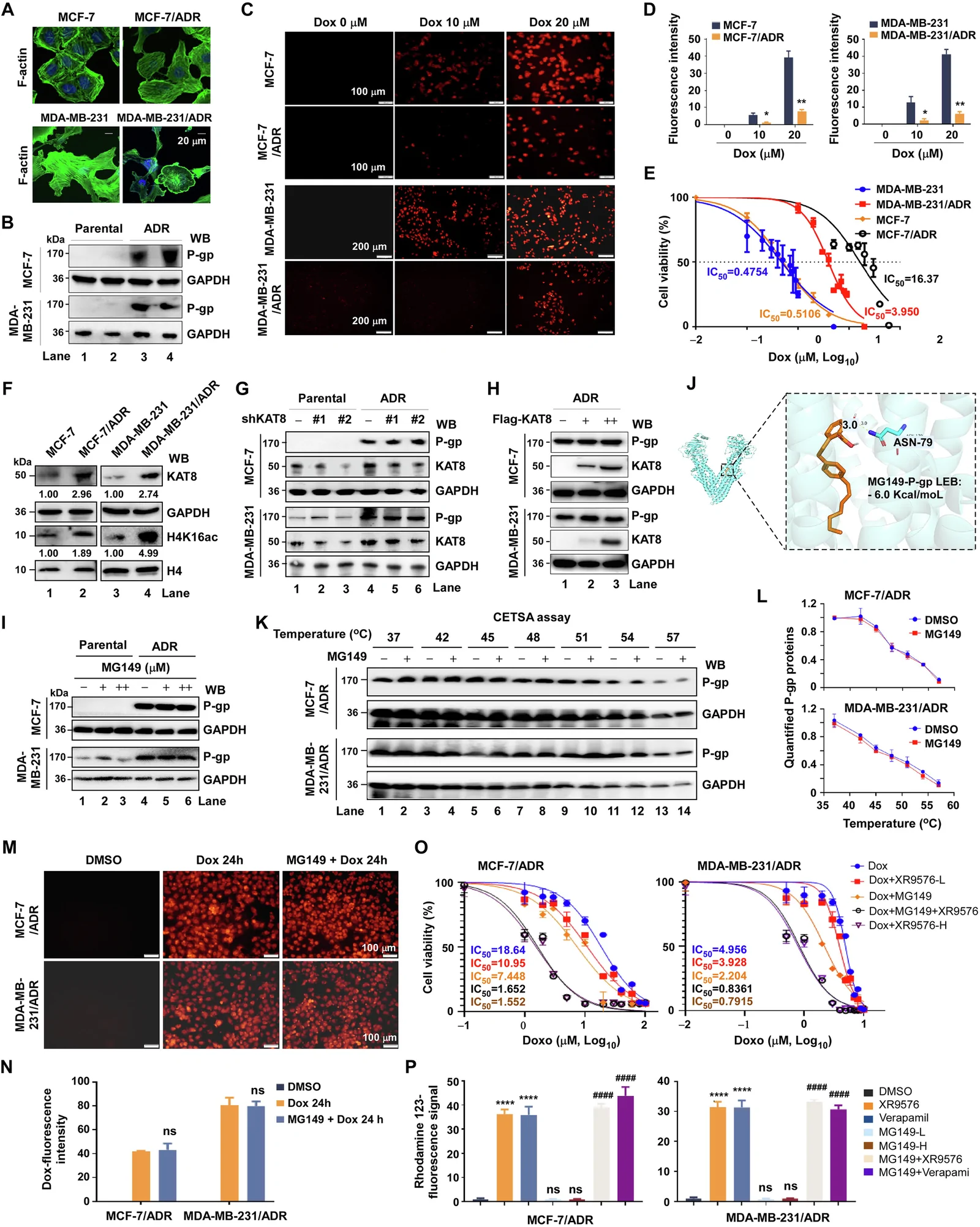
KAT8-mediated Dox resistance is independent of P-gp activity. **A** Phalloidin staining of F-actin showing morphological differences between parental and ADR breast cancer cells. **B** Immunoblot analysis of P-gp expression in parental and resistant cells. **C**, **D** Intracellular Dox fluorescence imaging and quantitative analysis. **E** Dox dose–response curves and corresponding IC_50_ values in parental and ADR cells. **F** Immunoblot analysis of KAT8 and H4K16ac in parental and ADR cells. **G**–**I** P-gp protein levels following KAT8 knockdown, overexpression or MG149 treatment. **J** Molecular docking analysis of MG149 with P-gp. **K** Effects of MG149 on P-gp abundance and thermal stability assessed by CETSA. **L** Quantification of CETSA results. **M**, **N** Intracellular Dox fluorescence and quantification following MG149 treatment. **O** Dox dose–response curves under MG149 treatment and/or pharmacological P-gp inhibition. **P** Rhodamine 123 accumulation assay assessing P-gp efflux activity. ns, not significant. *P < 0.05, **P < 0.01, ***P < 0.001, ****P < 0.0001.

Dox-resistant breast cancer cell lines MCF-7/ADR and MDA-MB-231/ADR exhibited marked morphological alterations compared with their parental counterparts, including thinner F-actin fibers and a shift from polygonal to elongated cell shapes (Fig. 2A). Immunoblotting confirmed upregulation of P-glycoprotein (P-gp), consistent with a multidrug-resistant phenotype [21, 22] (Fig. 2B). Correspondingly, intracellular Dox accumulation was significantly reduced in resistant cells (Fig. 2C, D). CCK-8 assays demonstrated substantially elevated IC_50_ in resistant lines (MCF-7 vs. MCF-7/ADR: 0.51 µM vs. 16.37 µM; MDA-MB-231 vs. MDA-MB-231/ADR: 0.48 µM vs. 3.95 µM), confirming robust drug resistance (Fig. 2E). Notably, resistant cells displayed markedly increased KAT8 expression accompanied by elevated H4K16ac levels (Fig. 2F).

To determine whether KAT8 modulates Dox response through regulation of P-gp, we assessed P-gp expression levels following KAT8 knockdown, KAT8 overexpression, or pharmacological inhibition with MG149. Neither genetic manipulation nor MG149 treatment substantially altered P-gp protein levels (Fig. 2G–I). Molecular docking predicted weak binding affinity between MG149 and P-gp, and cellular thermal shift assays (CETSA) failed to demonstrate a direct stabilizing interaction between MG149 and P-gp (Fig. 2J–L). Consistently, MG149 did not increase intracellular Dox accumulation in resistant cells (Fig. 2M, N). In contrast, established P-gp inhibitors, including XR-9576 and verapamil, significantly enhanced Rhodamine 123 accumulation, confirming functional P-gp activity, whereas MG149 alone did not induce a P-gp-like accumulation phenotype (Fig. 2O, P). Collectively, these results indicate that KAT8-mediated Dox resistance occurs largely independently of P-gp-driven drug efflux and instead involves alternative resistance mechanisms.

### Targeted inhibition of KAT8 sensitizes drug-resistant breast cancer cells to doxorubicin

**Fig. 3:**
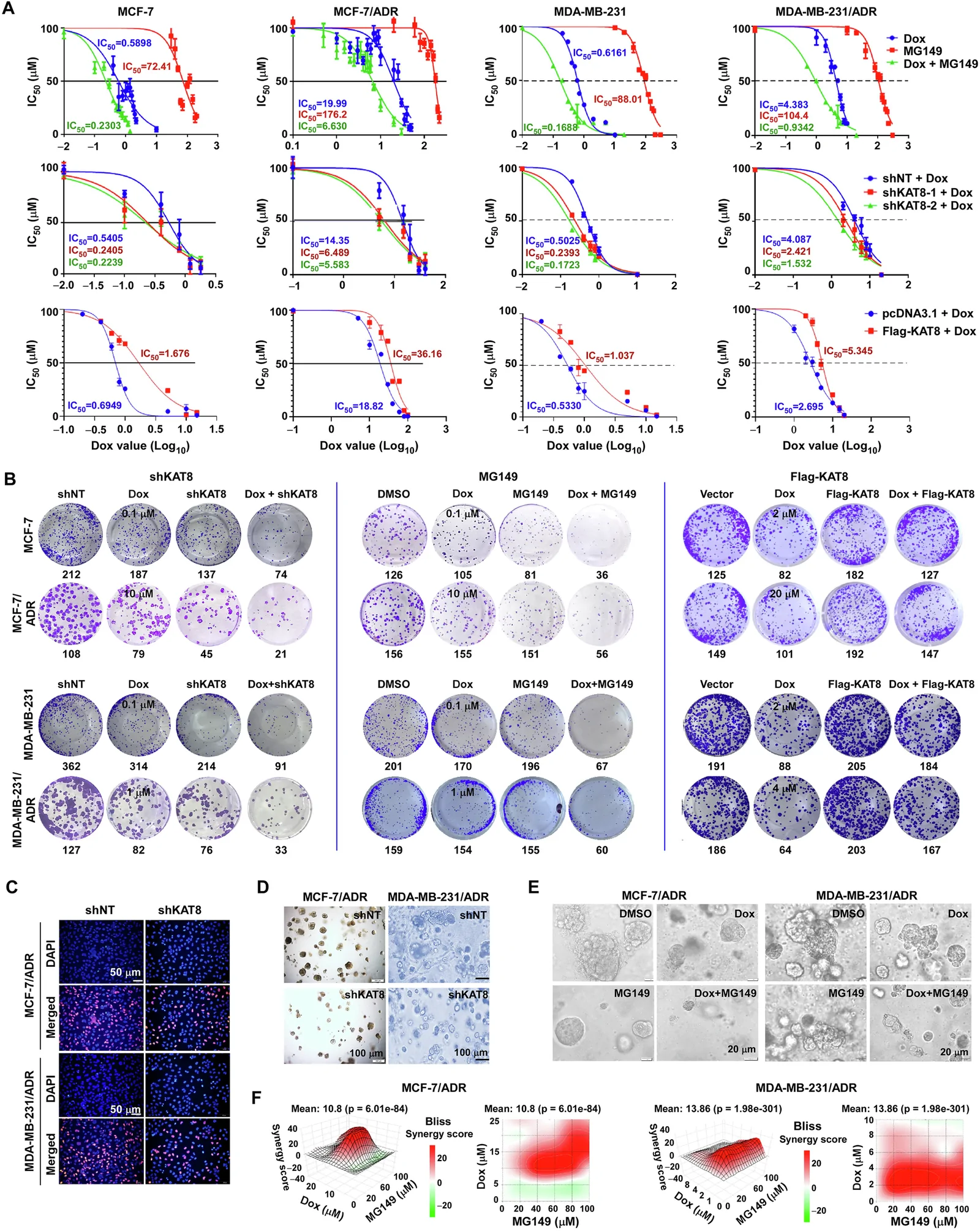
KAT8 inhibition sensitizes drug-resistant breast cancer cells to Dox. **A** Dox dose–response curves in MCF-7, MDA-MB-231, and their resistant derivatives following treatment with MG149, shKAT8, or KAT8 overexpression. **B** Colony formation assays under the indicated treatments. **C** EdU incorporation assays assessing cell proliferation. **D**, **E** 3D spheroid growth in the presence or absence of MG149 and Dox. **F** Bliss independence analysis demonstrating synergistic effects between MG149 and Dox in resistant cells.

To determine whether KAT8 modulates Dox sensitivity, parental and resistant breast cancer cells were treated with the KAT8 inhibitor MG149. Co-treatment with MG149 and Dox induced a pronounced leftward shift in the dose-response curves and significantly reduced IC_50_ values in both sensitive and resistant lines (MCF-7: 0.59 → 0.23 µM; MDA-MB-231: 0.62 → 0.17 µM; MCF-7/ADR: 19.99 → 6.63 µM; MDA-MB-231/ADR: 4.38 → 0.93 µM; Fig. 3A, upper panel). Notably, the IC_50_ values of MG149 alone were higher in resistant cells than in parental cells (MCF-7/ADR, 176.2 µM; MDA-MB-231/ADR, 104.4 µM vs. MCF-7, 72.4 µM; MDA-MB-231, 88.1 µM), indicating that the enhanced Dox response was not attributable to nonspecific cytotoxicity. Consistently, genetic silencing of KAT8 sensitized all cell lines to Dox, whereas KAT8 overexpression attenuated Dox responsiveness (Fig. 3A, middle and lower panels).

Colony formation assays further demonstrated that combining Dox with either shKAT8 or MG149 markedly suppressed clonogenic survival in both parental and resistant cells. In contrast, KAT8 overexpression partially rescued colony formation under high-dose Dox exposure (Fig. 3B). The chemosensitizing effect of KAT8 inhibition was particularly pronounced in resistant cells. Mechanistically, KAT8 depletion reduced EdU incorporation (Fig. 3C, quantified in Fig. S1B), impaired 3D spheroid growth (Fig. 3D, quantified in Fig. S1C), and enhanced mitochondrial depolarization, as indicated by JC-1 staining (Fig. S1D). Combined MG149 and Dox treatment suppressed spheroid expansion more effectively than either agent alone (Figs. 3E, S1E), and Bliss independence analysis confirmed significant synergistic effects in resistant lines (Fig. 3F). Given that MG149 targets both KAT5 and KAT8, we generated KAT8-knockout resistant cells to assess target specificity. In these cells, MG149 failed to further enhance Dox sensitivity beyond KAT8 loss alone, supporting a KAT8-dependent mechanism in our models (Fig. S1F).

Collectively, these findings demonstrated that both pharmacological and genetic inhibition of KAT8 effectively restores Dox sensitivity in resistant breast cancer cells, highlighting KAT8 as a potential therapeutic target to overcome chemoresistance.

### Suppression of the KAT8-CDK1 axis enhances doxorubicin sensitivity in resistant breast cancer cells

**Fig. 4:**
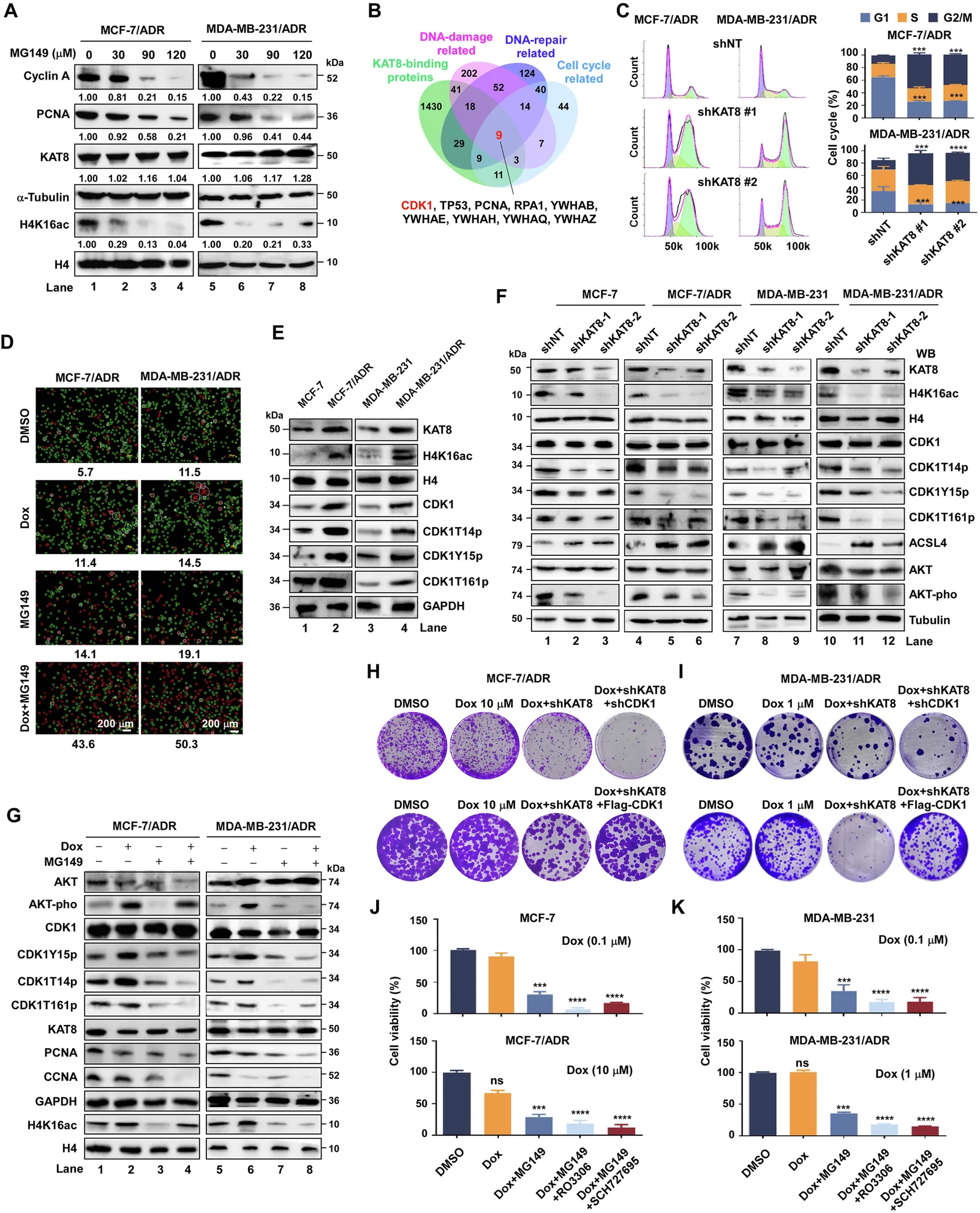
Suppression of the KAT8–CDK1 axis enhances Dox sensitivity. **A** Immunoblot analysis of H4K16ac, PCNA, and Cyclin A following MG149 treatment. **B** Identification of candidate KAT8-interacting proteins involved in DNA damage and cell-cycle regulation. **C** Flow cytometric analysis showing G2/M arrest following KAT8 knockdown. **D** AO/PI staining evaluating cell viability under indicated treatments. **E**–**G** CDK1 phosphorylation (T14, Y15, T161) and ACSL4 expression in resistant cells with or without KAT8 inhibition (genetic or pharmacological). **H**, **I** Colony formation assays in shKAT8 cells with additional CDK1 knockdown or overexpression. **J**, **K** CCK-8 cell viability assays assessing Dox sensitivity in the presence or absence of MG149 and/or CDK inhibitors.

Having established that KAT8-mediated Dox resistance occurs independently of P-gp, we next investigated alternative mechanisms involving cell-cycle regulation. Pharmacological inhibition of KAT8 with MG149 dose-dependently reduced H4K16ac levels without affecting total KAT8 protein and concomitantly decreased the proliferation markers PCNA and Cyclin A (Fig. 4A). Integrative analysis of KAT8-interacting proteins together with DNA damage and cell-cycle regulators identified nine candidate effectors, including CDK1, PCNA, and RPA, implicating KAT8 in cell-cycle control (Fig. 4B). Consistently, KAT8 knockdown induced G2/M arrest, accompanied by Cyclin B1 accumulation and increased BAX expression (Figs. 4C, S2A). Acridine orange/propidium iodide (AO/PI) staining further demonstrated that MG149 significantly potentiated Dox-induced cell death compared with either treatment alone (Fig. 4D).

Resistant cells exhibited elevated KAT8 and H4K16ac levels, together with increased CDK1 phosphorylation at T14, Y15, and T161 (Fig. 4E). These phosphorylation events were markedly reduced following KAT8 knockdown (Fig. 4F) or MG149 treatment (Fig. 4G). In line with previous evidence that CDK1 promotes ACSL4 degradation via phosphorylation [23], inhibition of KAT8 reduced CDK1 activity and restored ACSL4 protein levels (Fig. 4F, IB: ACSL4). Functionally, combined shKAT8 and Dox treatment significantly suppressed clonogenic survival, an effect further enhanced by CDK1 silencing. Conversely, CDK1 overexpression partially rescued colony formation under combined Dox and shKAT8 exposure (Fig. 4H, I; quantification in Fig. S2B, C). CCK-8 assays yielded consistent results: MG149 augmented Dox-induced cytotoxicity, and this effect was further potentiated by the CDK1 inhibitor RO3306 or the pan-CDK inhibitor SCH727965 (Fig. 4J, K).

Collectively, these data demonstrate that KAT8 promotes Dox resistance in MCF-7/ADR and MDA-MB-231/ADR cells through regulation of CDK1 kinase activity, thereby sustaining cell-cycle progression and cellular survival under chemotherapeutic stress.

### KAT8-driven CDK1 K33 acetylation regulates CDK1 phosphorylation and activation

**Fig. 5:**
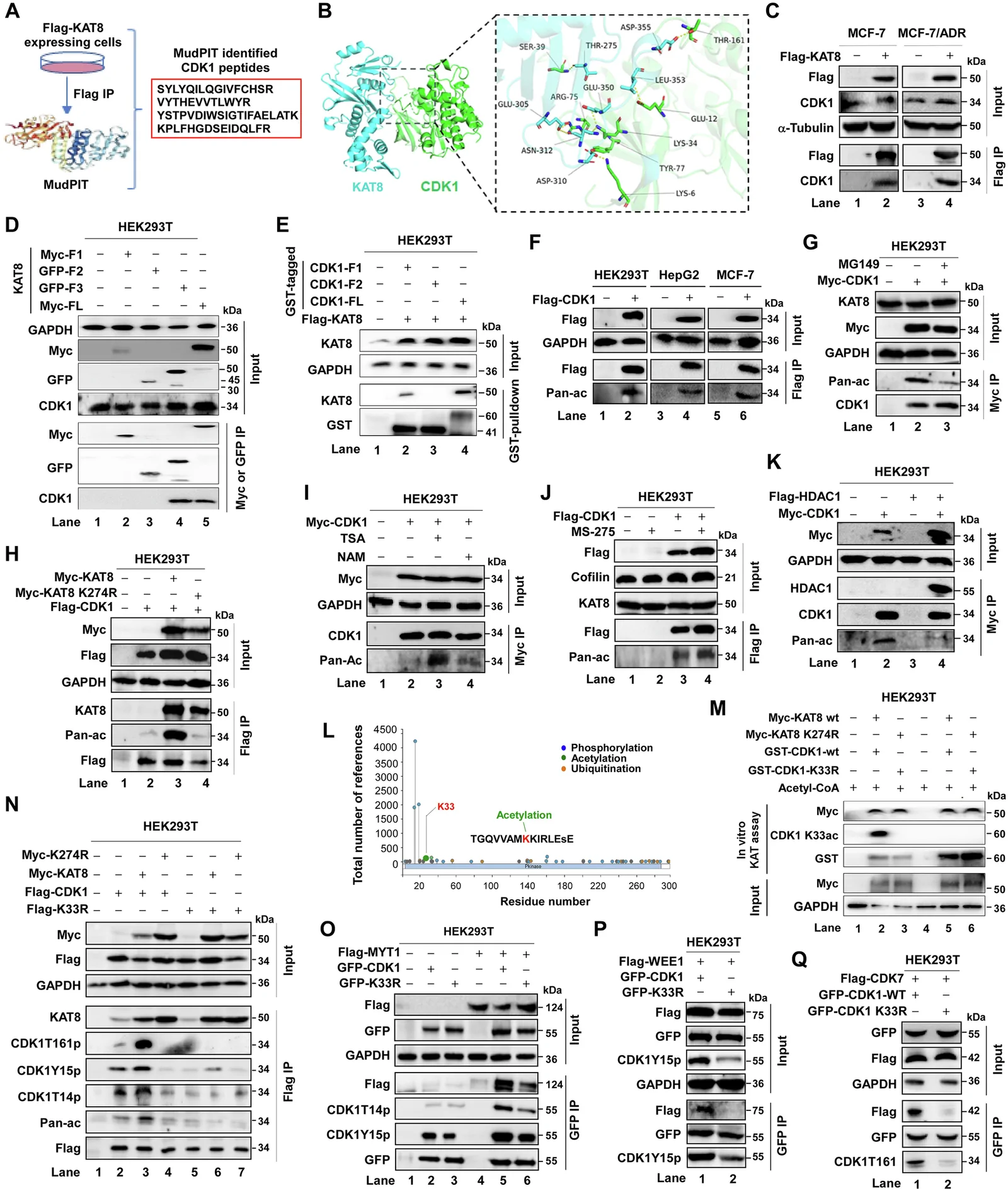
KAT8 acetylates CDK1 at K33 to regulate phosphorylation and activation. **A** MudPIT mass spectrometry analysis of Flag-KAT8 immunoprecipitates identifying CDK1 derived peptides (detected peptide sequences highlighted in red). **B** Predicted hydrogen-bond interactions between KAT8 and CDK1 based on molecular docking analysis. **C**–**E** Co-IP, GST pull-down, and truncation mapping analyses defining the KAT8–CDK1 interaction domains. **F**–**H** CDK1 acetylation depends on KAT8 expression and catalytic activity. **I**, **J** HDAC inhibitor screening identifying HDAC1 as the principal CDK1 deacetylase. **K** HDAC1 overexpression reduces CDK1 acetylation. **L**, **M** Identification of K33 as the major acetylation site; the CDK1-K33R mutant is non-acetylatable. **N**–**Q** K33 acetylation is required for CDK1 phosphorylation at T14, Y15, T161 as well as for interaction with upstream kinases (MYT1, WEE1, CDK7).

Mass spectrometric analysis of Flag-KAT8 immunoprecipitates identified CDK1-derived peptides, implicating CDK1 as a KAT8-associated protein (Fig. 5A). Molecular docking predicted extensive contact interactions between KAT8 and CDK1 (Fig. 5B). Co-immunoprecipitation assays confirmed their interaction in breast cancer cells (Fig. 5C). Truncation mapping and GST pull-down assays further demonstrated that the MYST domain of KAT8 directly interacts with the N-terminal region of CDK1 (Fig. 5D, E).

Flag-CDK1 displayed robust acetylation across multiple cell types, which was markedly reduced by MG149 treatment or KAT8 knockdown (Figs. 5F, G, S3A). Reconstitution experiments showed that wild-type (WT) KAT8, but not the catalytically inactive KAT8-K274R mutant, restored CDK1 acetylation (Fig. 5H), indicating dependence on KAT8 enzymatic activity. Deacetylase inhibitor screening revealed that TSA and the HDAC1/3-selective inhibitor MS-275 increased CDK1 acetylation, whereas NAM had no effect. Conversely, HDAC1 overexpression significantly decreased CDK1 acetylation (Fig. 5I–K), identifying HDAC1 as a principal deacetylase opposing KAT8-mediated modification.

PhosphoSitePlus analysis identified lysine 33 (K33) as a conserved acetylation site within CDK1 (Fig. 5L). In vitro lysine acetylation (KAT) assays demonstrated that KAT8 efficiently acetylated WT CDK1 but not the CDK1-K33R mutant, while KAT8-K274R lacked acetyltransferase activity (Fig. 5M). Disruption of K33 acetylation, either by expressing KAT8-K274R or CDK1-K33R, significantly reduced phosphorylation at T14, Y15, and T161 (Fig. 5N). Moreover, the CDK1 regulatory kinases MYT1, WEE1 and CDK7 effectively phosphorylated WT CDK1, whereas both phosphorylation efficiency and kinase binding were attenuated in the K33R mutant (Fig. 5O–Q). Consistently, CDK1-K33R exhibited diminished kinase activity toward STAT3 and AKT substrates (Fig. S3B, C). Consistently, upon Dox induction, wild-type CDK1 showed enhanced phosphorylation of its substrates, including SOD2, ATP5, and SIRT3. In contrast, the deacetylation-mimetic mutant CDK1-K33R displayed significantly diminished phosphorylation of these substrates (Fig. S3D). Collectively, these results demonstrate that acetylation at K33 not only modulates CDK1 interaction with upstream regulatory kinases (MYT1/WEE1/CDK7), but also directly enhances its intrinsic kinase activity. Loss of K33 acetylation therefore disrupts both upstream regulatory phosphorylation and downstream substrate phosphorylation, thereby functionally interrupting the mechanistic signaling cascade.

### CDK1 K33 acetylation is elevated in doxorubicin-resistant breast cancer cells and governs drug responsiveness

**Fig. 6:**
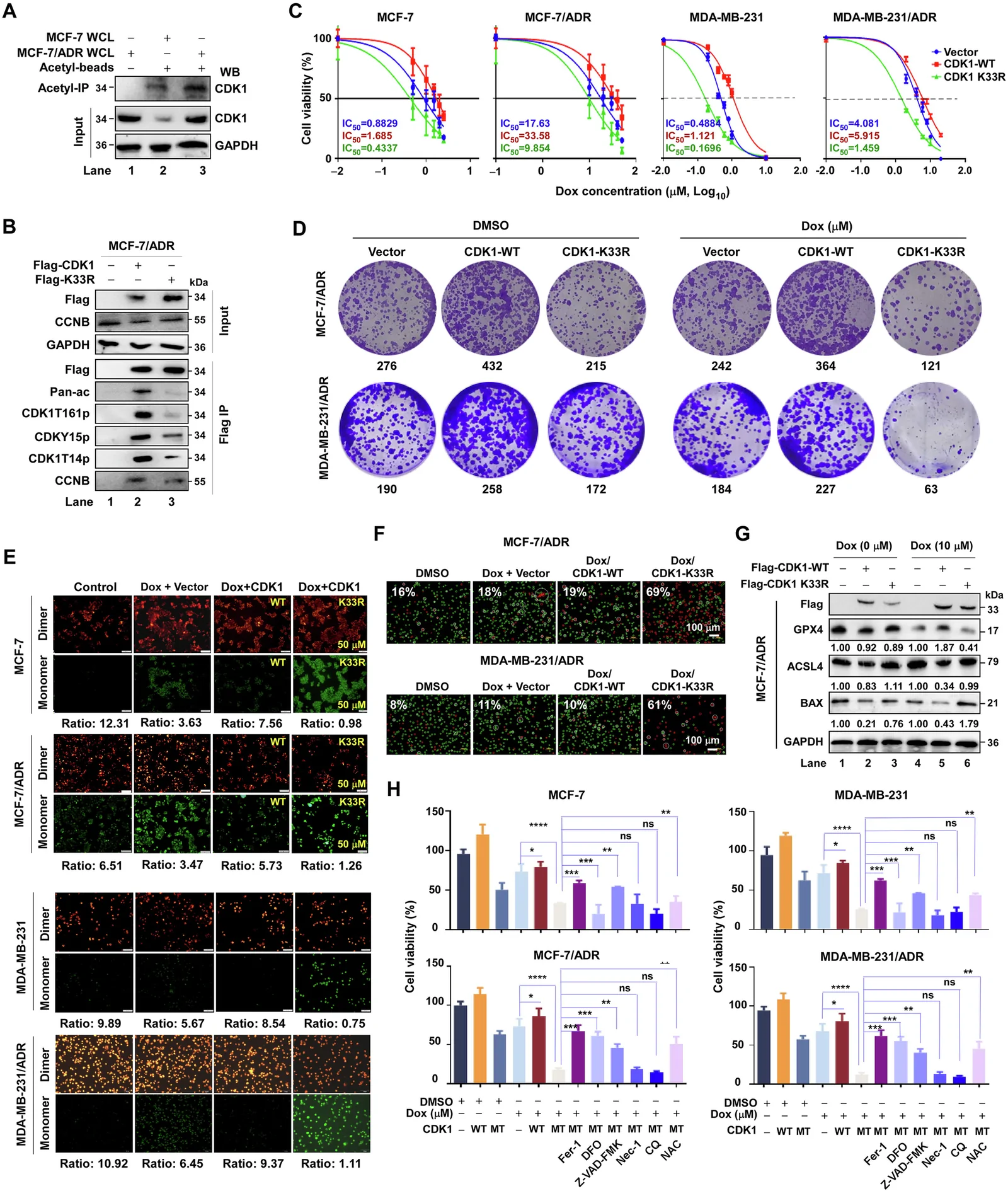
CDK1 K33 acetylation is elevated in resistant cells and governs Dox responsiveness. **A** Enrichment analysis of acetylated CDK1 in MCF-7 and MCF-7/ADR cells. **B** Flag IP showing that the K33 mutation reduces CDK1 acetylation and phosphorylation at T14, Y15, and T161. **C** CCK-8 dose-response curves and IC_50_ analysis of CDK1-K33R-expressing cells. **D** Colony formation assays of CDK1-K33R-expressing cells under Dox treatment. **E** JC-1 staining assessing mitochondrial membrane depolarization. **F** AO/PI staining evaluating cell death in CDK1-WT and CDK1-K33R-expressing cells. **G** Immunoblot analysis of ACSL4, BAX, and GPX4 expression. **H** Rescue experiments using ferroptosis, apoptosis, and ROS inhibitors.

To investigate whether the KAT8–CDK1 K33 acetylation axis contributes to chemoresistance in breast cancer, acetylated proteins were enriched from MCF-7 and Dox-resistant MCF-7/ADR cells. CDK1 acetylation was markedly increased in resistant cells (Fig. 6A). Expression of the acetylation-deficient mutant CDK1-K33R in MCF-7/ADR cells substantially reduced CDK1 acetylation and concomitantly decreased phosphorylation at T14, Y15, and T161 compared with WT CDK1 (Fig. 6B). Functionally, CDK1-K33R significantly sensitized both parental and resistant cells to Dox, as evidenced by decreased IC_50_ values, left-shifted dose–response curves, and reduced clonogenic survival (Fig. 6C, D). Consistently, CDK1-K33R-expressing cells exhibited enhanced cell death under Dox treatment, as shown by AO/PI staining, along with pronounced mitochondrial depolarization detected by JC-1 assay (Fig. 6E, F). Mechanistically, CDK1-K33R reversed the regulatory effects of CDK1-WT on ferroptosis- and apoptosis-related proteins, including decreased GPX4 expression and increased ACSL4 and BAX levels, indicating elevated susceptibility to ferroptotic and apoptotic stress (Fig. 6G). Pharmacological rescue experiments using Ferrostatin-1 (Fer-1), deferoxamine (DFO), Z-VAD-FMK, N-acetylcysteine (NAC), and other inhibitors partially restored cell viability, further supporting the involvement of multiple regulated cell death pathways (Fig. 6H). Given that Dox induces DNA damage and cell-cycle disruption, we further examined whether CDK1 K33 acetylation regulates these processes. KAT8 depletion resulted in sustained γ-H2AX accumulation following Dox treatment, indicating impaired resolution of DNA damage signaling. This effect was rescued by CDK1-WT but not by CDK1-K33R (Fig. S3E, F). In addition, KAT8 knockdown induced G2/M cell-cycle arrest, which was partially reversed by CDK1-WT but further exacerbated by CDK1-K33R (Fig. S3G, H). Collectively, these findings demonstrated that CDK1 K33 acetylation is elevated in Dox-resistant cells and is functionally required for the maintenance of chemoresistance. Mechanistically, KAT8-mediated CDK1 K33 acetylation promotes Dox resistance by coordinating DNA damage response and cell-cycle progression, together with regulation of ferroptotic and apoptotic susceptibility.

### KAT8–mediated CDK1 K33 acetylation sustains doxorubicin resistance by suppressing ferroptosis and apoptosis

**Fig. 7:**
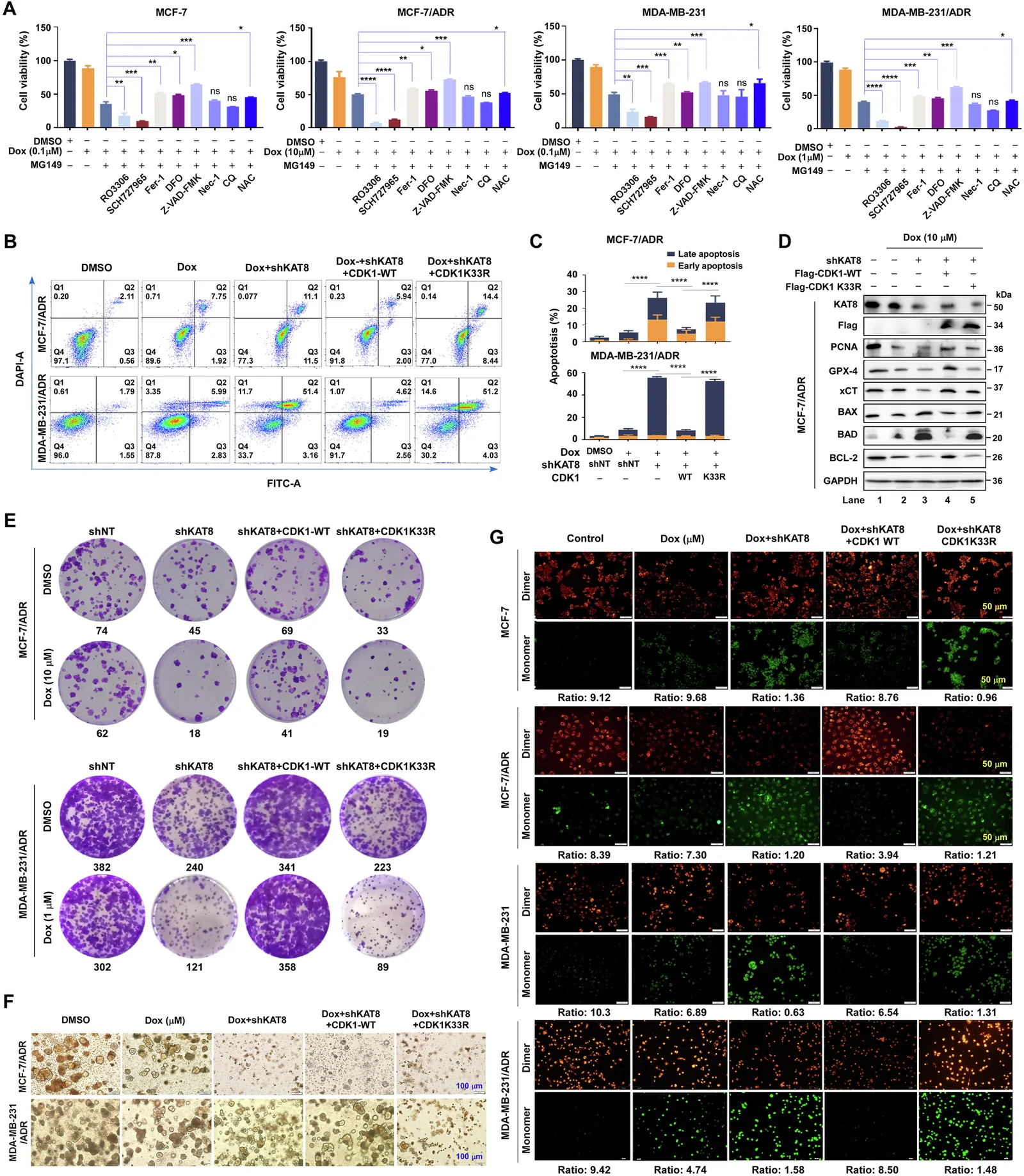
KAT8-mediated CDK1 K33 acetylation suppresses ferroptosis and apoptosis. **A** CCK-8 viability assays following treatment with Dox + MG149 in the presence or absence of CDK inhibitors or cell-death modulators. **B**, **C** Annexin V apoptosis analysis in CDK1-WT or CDK1-K33R-expressing cells with or without KAT8 knockdown. **D** Immunoblot analysis of pro-apoptotic (BAX, BAD) and anti-ferroptotic (xCT, GPX4) proteins. **E** Cell viability and clonogenic assays demonstrating restoration of chemoresistance by WT CDK1 but not CDK1-K33R. **F** 3D spheroid growth assays with shKAT8 and CDK1 mutants. **G** JC-1 staining showing mitochondrial membrane potential under the indicated treatments.

To delineate the role of the KAT8–CDK1 axis in Dox resistance, MCF-7/ADR and MDA-MB-231/ADR cells were treated with Dox and the KAT8 inhibitor MG149, alone or in combination with CDK inhibitors (RO3306, SCH727965) or cell-death modulators, including Fer-1, DFO, Z-VAD-FMK, Necrostatin-1 (Nec-1), chloroquine (CQ), and NAC. CCK-8 assays showed that CDK1 inhibition further enhanced Dox sensitivity, whereas ferroptosis and apoptosis inhibitors (Fer-1, DFO, Z-VAD-FMK, NAC) partially restored cell viability (Fig. 7A), indicating that MG149-induced cytotoxicity is CDK1-dependent and mediated through ferroptotic and apoptotic pathways. In addition, combined treatment with the ferroptosis inhibitor (Fer-1) and the apoptosis inhibitor (Z-VAD-FMK) produced a more pronounced rescue effect (Fig. S4A). Annexin V/PI staining revealed a marked increase in apoptosis in Dox-treated shKAT8 cells, which was reversed by WT CDK1 but not by the acetylation-deficient mutant CDK1-K33R (Fig. 7B, C). Consistently, KAT8 depletion upregulated pro-apoptotic proteins BAX and BAD while reducing the anti-ferroptotic factors xCT and GPX4. WT CDK1 restored xCT and GPX4 expression and suppressed BAX and BAD, whereas CDK1-K33R failed to do so (Fig. 7D). Colony formation and spheroid assays further demonstrated that WT CDK1 rescued chemoresistance in KAT8-depleted cells, whereas CDK1-K33R did not (Fig. 7E, F). JC-1 staining showed that WT CDK1 preserved mitochondrial membrane potential, while CDK1-K33R phenocopied KAT8 depletion (Fig. 7G). Genetic rescue experiments corroborated these findings: modulation of GPX4 or ACSL4 altered lipid peroxidation accordingly, and BAX knockdown attenuated apoptosis induced by disruption of the KAT8–CDK1 axis (Fig. S4B–D).

Moreover, Dox-treated KAT8-knockdown cells displayed elevated ROS levels (Fig. S5A, B), reduced GSH (Fig. S5C), increased MDA accumulation (Fig. S5D), and enhanced lipid peroxidation (Fig. S5E). WT CDK1 restored redox balance, whereas CDK1-K33R further aggravated oxidative stress, underscoring the importance of CDK1 K33 acetylation in maintaining mitochondrial and redox homeostasis. Collectively, these data demonstrate that KAT8-mediated acetylation of CDK1 at K33 suppresses ferroptosis and apoptosis, thereby sustaining Dox resistance in breast cancer cells. Targeting this regulatory modification may provide a promising strategy to overcome chemoresistance.

### Disruption of the KAT8–CDK1 K33 acetylation axis sensitizes breast cancer xenografts to doxorubicin

**Fig. 8:**
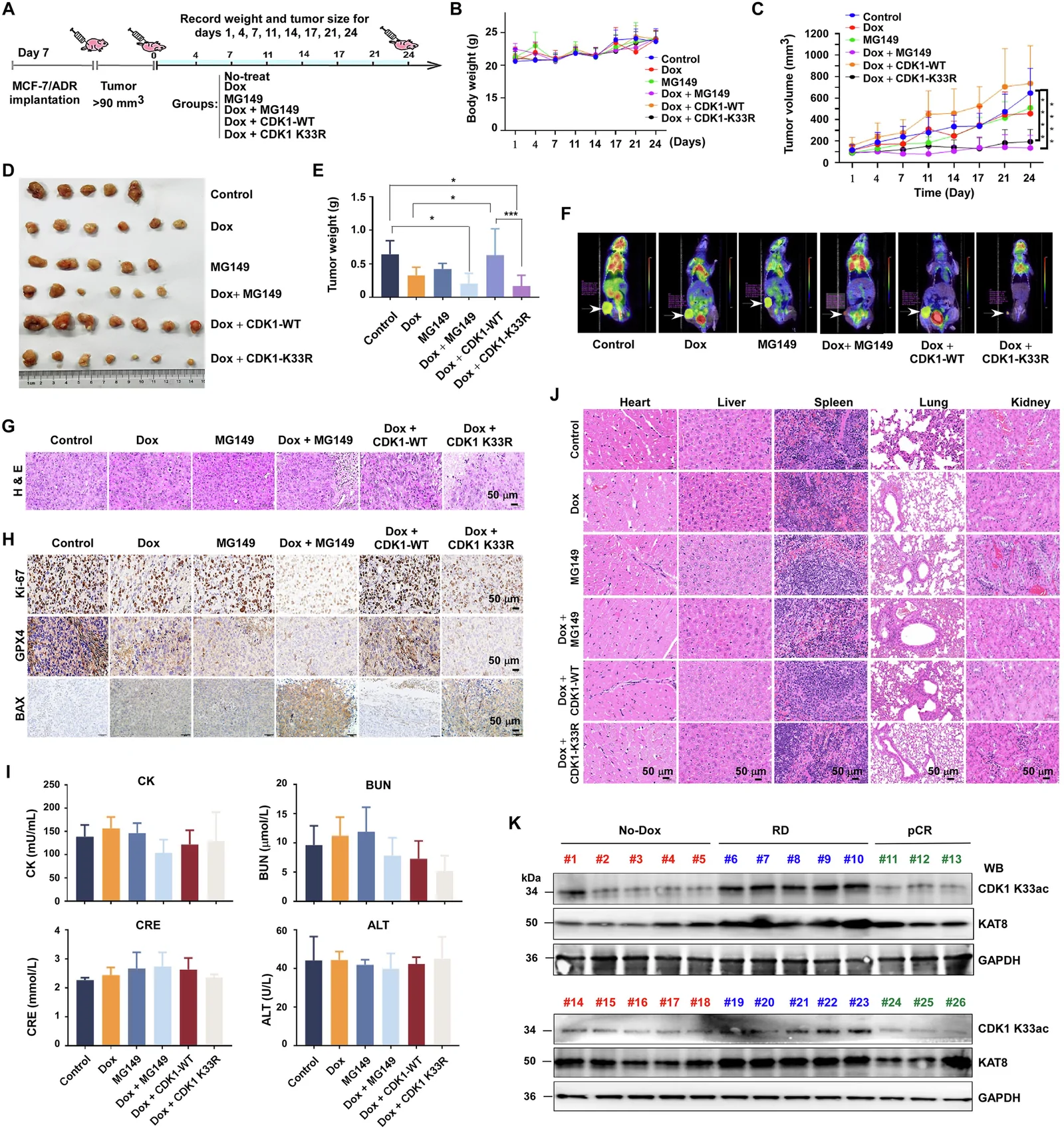
Disruption of the KAT8–CDK1 K33 acetylation axis sensitizes breast cancer xenografts to Dox. **A** Schematic overview of the xenograft experimental design. **B** Body weight monitoring during treatment. **C**–**E** Tumor growth curves and endpoint tumor weights following treatment with Dox, MG149, CDK1-WT + Dox, or CDK1-K33R + Dox. **F** PET-CT imaging showing tumor burden and metabolic activity. **G** Representative H&E staining of tumor sections. **H** IHC analysis of Ki-67, BAX, and GPX4 expression. **I** Serum biochemical analyses of CK, CRE, BUN, ALT. **J** H&E staining of major organs (heart, liver, spleen, lung, and kidney) for toxicity assessment. **K** Immunoblot analysis of CDK1 K33 acetylation and KAT8 expression in clinical No-Dox, RD, and pCR breast cancer samples.

To validate the functional relevance of the KAT8–CDK1 axis in vivo, STR-authenticated MCF-7/ADR cells were subcutaneously implanted into nude mice without estrogen supplementation. Once tumors reached 80–100 mm³, mice were randomized to receive saline, Dox, MG149, Dox + MG149, CDK1-WT + Dox, or CDK1-K33R + Dox (Fig. 8A). Body weights remained stable across groups (Fig. 8B), indicating no overt acute toxicity under the applied dosing regimen. Combined Dox + MG149 treatment and CDK1-K33R + Dox markedly reduced tumor growth and final tumor weight compared with Dox alone, whereas CDK1-WT attenuated the antitumor efficacy of Dox (Fig. 8C–E). PET-CT imaging corroborated these findings, demonstrating reduced tumor size and metabolic activity in the combination and K33R groups (Fig. 8F). Histopathological analysis by H&E staining revealed pronounced structural disruption in tumors from the Dox + MG149 and CDK1-K33R + Dox groups (Fig. 8G). Immunohistochemistry analysis further showed decreased Ki-67 and GPX4 expression, alongside increased BAX, consistent with reduced proliferation and enhanced ferroptotic and apoptotic signaling (Fig. 8H). Serum biochemical markers (CK, CRE, BUN, ALT) were not significantly altered, and H&E staining of major organs showed no overt pathological abnormalities (Fig. 8I, J). Although these findings suggest preliminary tolerability, comprehensive pharmacokinetic and toxicological assessments will be required for further translational evaluation. Importantly, analysis of clinical tumor lysates using a CDK1 K33ac-specific antibody revealed elevated CDK1 K33 acetylation and KAT8 expression in RD tumors compared with No-Dox or pCR tumors (Fig. 8K), supporting activation of the KAT8–CDK1 K33ac axis in human Dox-resistant breast cancer. Collectively, these in vivo and clinical data demonstrate that disruption of KAT8-mediated CDK1 K33 acetylation sensitizes Dox-resistant breast cancer to chemotherapy by promoting ferroptosis and apoptosis, highlighting this axis as a potential therapeutic target for overcoming chemoresistance.

## Discussion

Doxorubicin (Dox) resistance remains a major obstacle in the treatment of breast cancer. Transcriptomic analyses of patients receiving neoadjuvant chemotherapy revealed significantly elevated KAT8 expression in tumors with RD, which correlated with unfavorable clinical outcomes. These findings were validated in independent clinical samples, establishing a strong association between high KAT8 levels and Dox resistance. Mechanistically, our study identifies the KAT8–CDK1 axis as a key determinant of chemoresistance in MCF-7/ADR and MDA-MB-231/ADR cells. KAT8 is upregulated in resistant cells, and both pharmacological inhibition and genetic depletion of KAT8 restore Dox sensitivity. At the molecular level, KAT8 directly acetylates CDK1 at K33, a modification that stabilizes phosphorylation at T14, Y15, and T161 and preserves CDK1 kinase activity. This acetylation-dependent regulation sustains cell-cycle progression and suppresses ferroptosis and apoptosis under chemotherapeutic stress. Inhibition of KAT8 disrupts CDK1 K33 acetylation, weakens CDK1 interactions with WEE1, MYT1, and CDK7, reduces CDK1 activity, and enhances Dox-induced mitochondrial depolarization. These molecular events culminate in impaired tumor growth and increased drug responsiveness in vitro and in xenograft models.

Importantly, KAT8 exerts pleiotropic functions beyond CDK1 regulation. Previous studies have demonstrated that KAT8 modifies or functionally regulates multiple non-histone substrates, including p53, YY1, PKM2, NSUN2-related RNA modification programs, and SEPP1, thereby influencing metabolic reprogramming, RNA modification, immune regulation, and drug response [24–28]. Thus, CDK1 K33 acetylation should be viewed as a central node within a broader KAT8 substrate network rather than the sole downstream effector. A more integrated interpretation is that KAT8 upregulation may simultaneously reinforce H4K16ac-dependent chromatin adaptation, PKM2-driven glycolytic metabolism, p53/YY1-mediated transcriptional programs, and CDK1-dependent anti-ferroptotic signaling. These coordinated pathways may collectively diminish the cytotoxic efficacy of Dox. While the identification of CDK1 K33 acetylation as an actionable regulatory hub represents a major strength of this study, future acetylome profiling, CUT&Tag/ChIP-seq, and transcriptomic analyses will be necessary to disentangle CDK1-dependent effects from contributions of additional KAT8 substrates.

Similarly, CDK1 should not be conceptualized simply as a kinase that is “activated” by K33 acetylation. CDK1 activity is governed by multilayered regulatory mechanisms, including Cyclin B1 association, inhibitory phosphorylation at T14/Y15, activating phosphorylation at T161, ubiquitination, subcellular localization, and potentially additional acetylation events [14, 29–31]. Our data suggest that K33 acetylation acts upstream of key phosphorylation events and modulates CDK1 interactions with WEE1, MYT1, and CDK7, thereby introducing a previously unrecognized layer of acetylation–phosphorylation crosstalk in CDK1 regulation. This mechanistic insight not only explains sustained CDK1 activity in resistant cells but also implies that therapeutic responsiveness to CDK1-targeted strategies may depend on the combinatorial post-translational modification (PTM) state rather than total CDK1 abundance alone.

Dox-based combination regimens remain a cornerstone of therapy for invasive and metastatic breast cancer [32–35]. We demonstrate that co-treatment with the KAT8 inhibitor MG149 markedly enhances chemosensitivity and suppresses proliferation in resistant models. Although KAT8 inhibitors have not yet advanced to clinical application [36–39], prior studies reported that MG149 sensitizes tumor cells to Dox and other chemotherapies, including paclitaxel and cyclophosphamide, albeit without defined molecular mechanisms. Our findings provide mechanistic clarification by establishing the KAT8–CDK1 axis as a key mediator of this sensitization effect. These data offer preclinical rationale for targeting KAT8 to potentiate anthracycline efficacy. Future studies should focus on optimizing KAT8 inhibitors, evaluating pharmacokinetics and safety, and defining downstream signaling networks to facilitate translational development.

CDK1 is a master regulator of cell-cycle progression, and its hyperactivation is associated with aggressive tumor behavior and poor prognosis [40–42]. Its activity is tightly controlled by phosphorylation at T14, Y15, and T161 [43–45]. We show that CDK1 is hyperactivated in Dox-resistant cells and that K33 acetylation is essential for maintaining phosphorylation at these sites, revealing a previously unrecognized regulatory layer.

A notable conceptual advance of this work is the demonstration that the KAT8–CDK1 axis simultaneously restrains apoptosis and ferroptosis, two principal modes of Dox-induced cell death. CDK1 activity appears indispensable for suppressing both pathways, positioning CDK1 as a central integrator of chemoresistance. Although ACSL4 has been implicated as a CDK1 substrate involved in ferroptosis regulation, additional downstream effectors likely contribute and warrant further investigation. Elucidating these components may uncover new therapeutic vulnerabilities. In summary, our study establishes the KAT8–CDK1 axis as a pivotal regulator of Dox resistance in breast cancer. Through acetylation-dependent maintenance of CDK1 activity, this pathway preserves cell-cycle progression and suppresses ferroptotic and apoptotic cell death. Targeting KAT8-mediated CDK1 K33 acetylation therefore represents a promising strategy to overcome anthracycline resistance and improve therapeutic outcomes in refractory breast cancer.

## Supplementary information

Supplemental_Methods_Figures and Table S1

Original Western blots

## Data Availability

The GSE25065 and GSE34138 datasets are available in the NCBI database https://www.ncbi.nlm.nih.gov/). All experimental data in this work are available from the corresponding author upon reasonable request.
