# Supplementary material for: The KAT8-CDK1 K33 acetylation axis drives doxorubicin resistance by suppressing ferroptosis and apoptosis in breast cancer
Source: Cell Death Dis. 2026 Aug 3;17(1):825. doi: 10.1038/s41419-026-09162-6 (PMC13601616; doi:10.1038/s41419-026-09162-6)
Supplement: Supplementary file 1 [file 41419_2026_9162_MOESM2_ESM.pdf]

Figure 1E

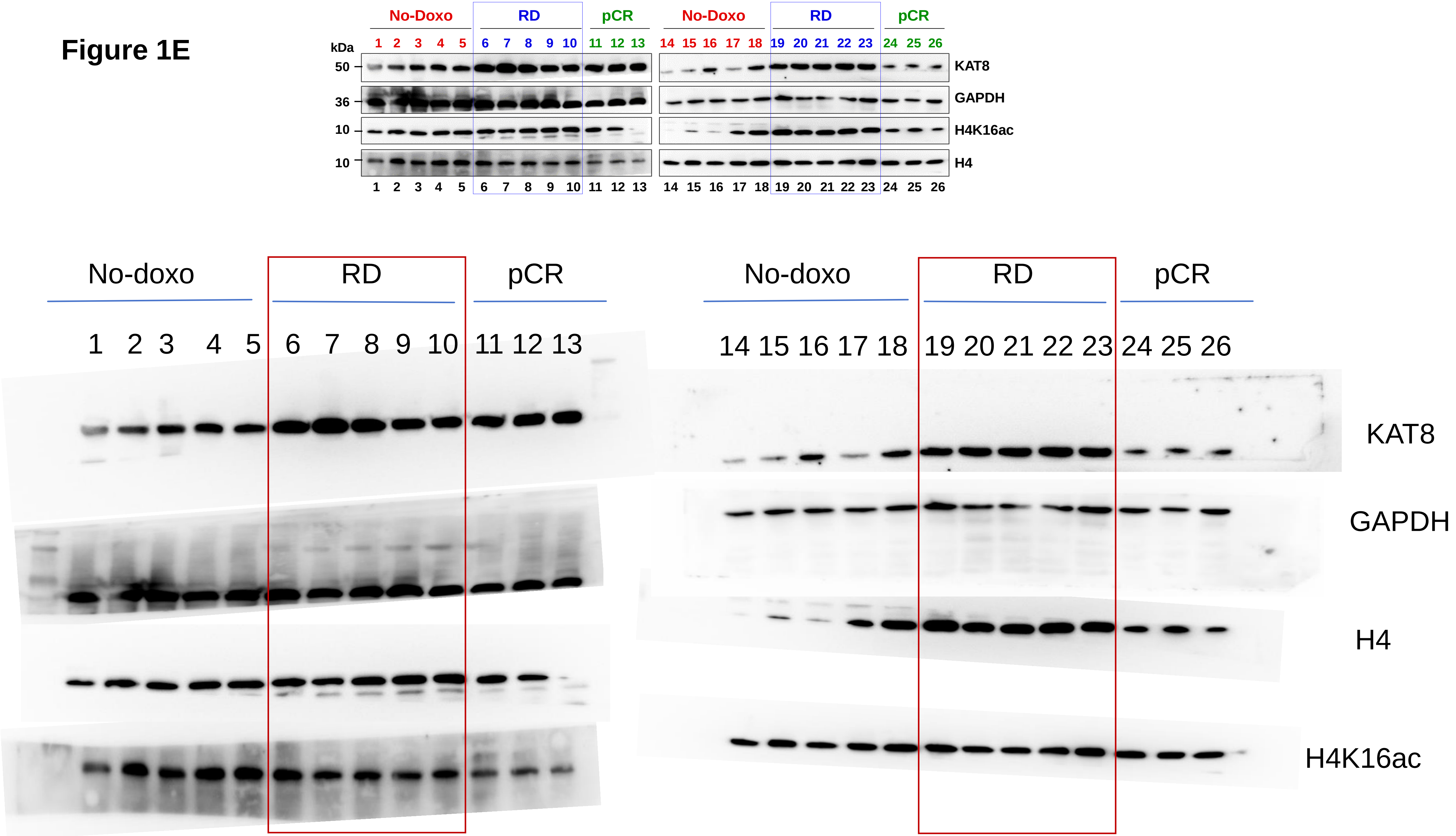
No-Doxo
RD
pCR
No-Doxo
RD
pCR
kDa
1 2 3 4 5 6 7 8 9 10 11 12 13 14 15 16 17 18 19 20 21 22 23 24 25 26
50
36
10
10
KAT8
GAPDH
H4K16ac
H4
No-doxo
RD
pCR
No-doxo
RD
pCR
1 2 3 4 5 6 7 8 9 10 11 12 13
14 15 16 17 18 19 20 21 22 23 24 25 26
KAT8
GAPDH
H4
H4K16ac

Figure 2B

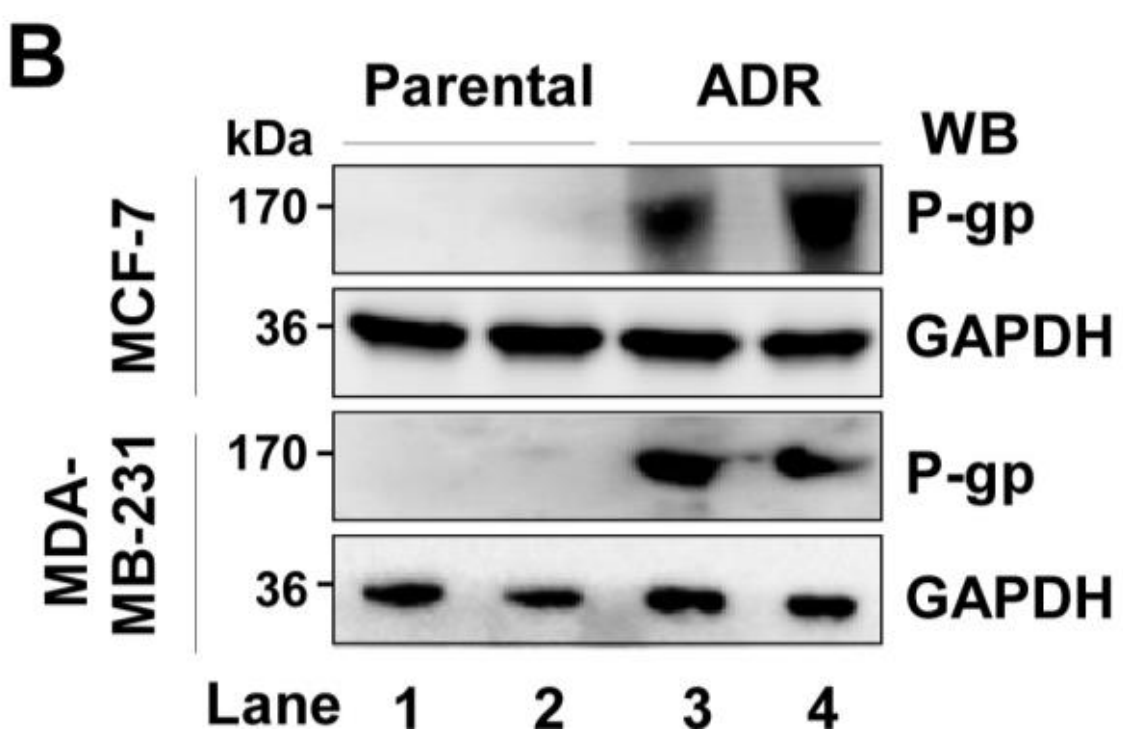
B
Parental
ADR
kDa
WB
MCF-7
170
P-gp
36
GAPDH
MDA-MB-231
170
P-gp
36
GAPDH
Lane 1 2 3 4

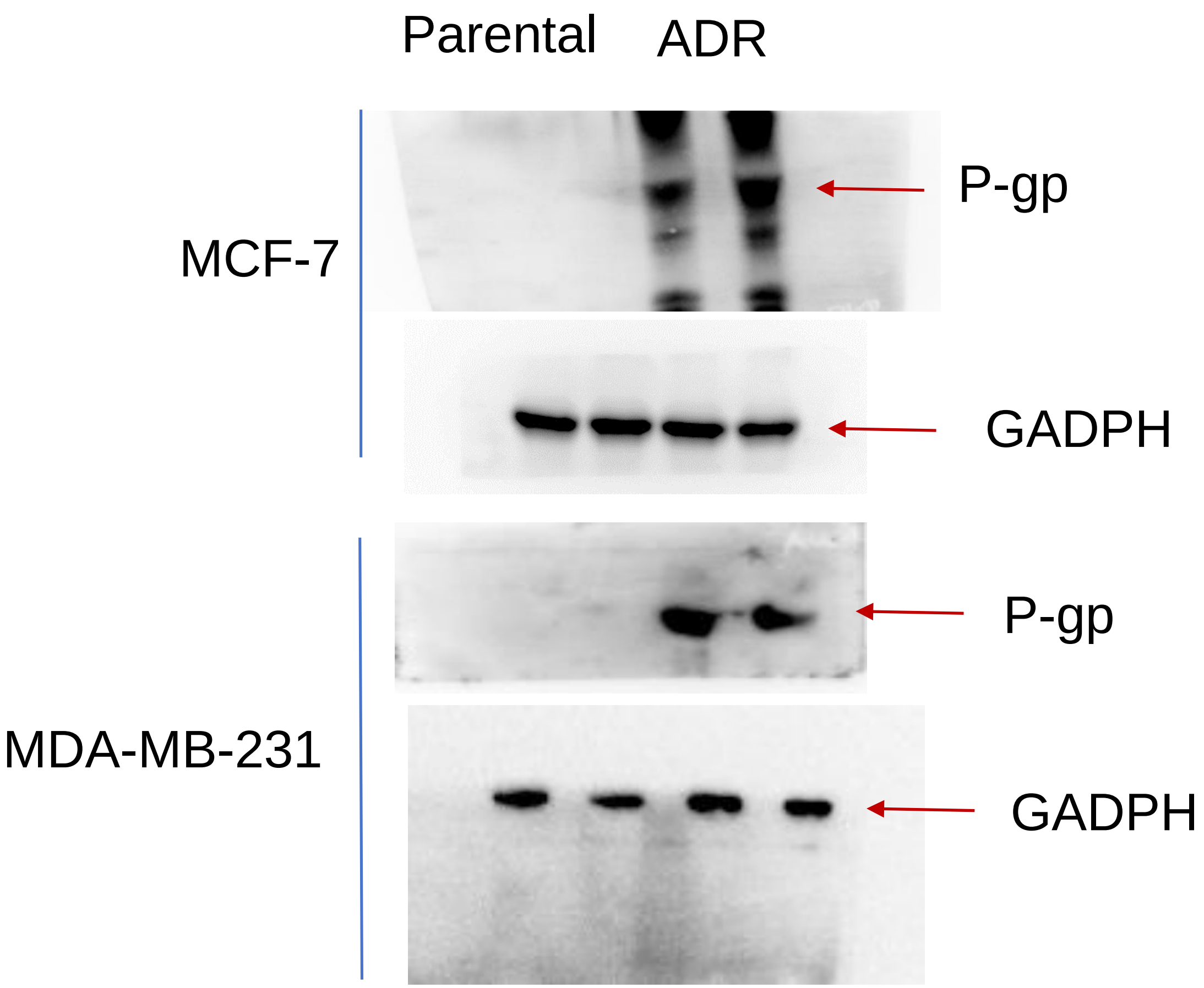
Parental
ADR
MCF-7
P-gp
GADPH
MDA-MB-231
P-gp
GADPH

Figure 2F

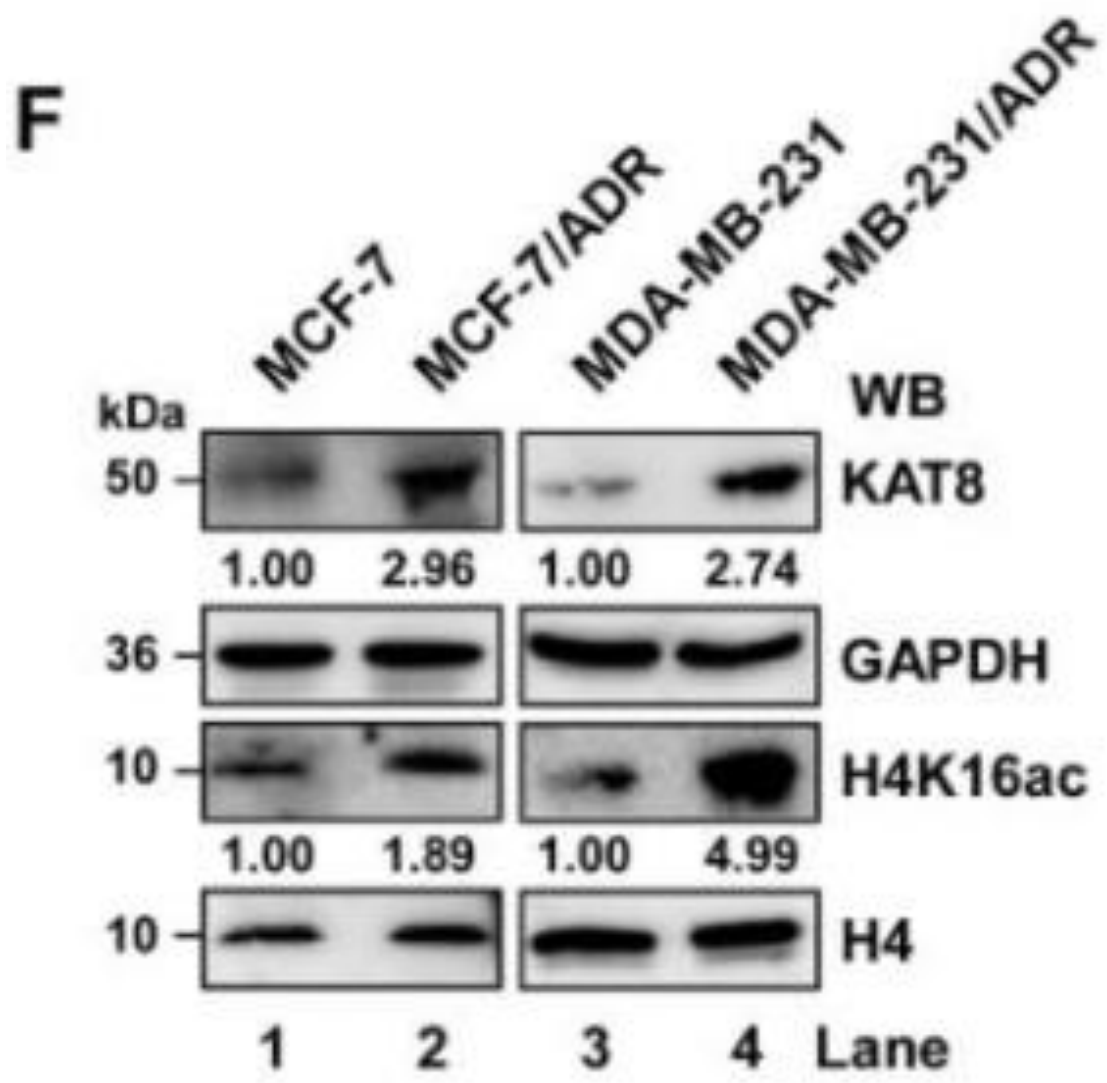
F
MCF-7
MCF-7/ADR
MDA-MB-231
MDA-MB-231/ADR
kDa
WB
50
KAT8
1.00 2.96 1.00 2.74
36
GAPDH
10
H4K16ac
1.00 1.89 1.00 4.99
10
H4
1 2 3 4 Lane

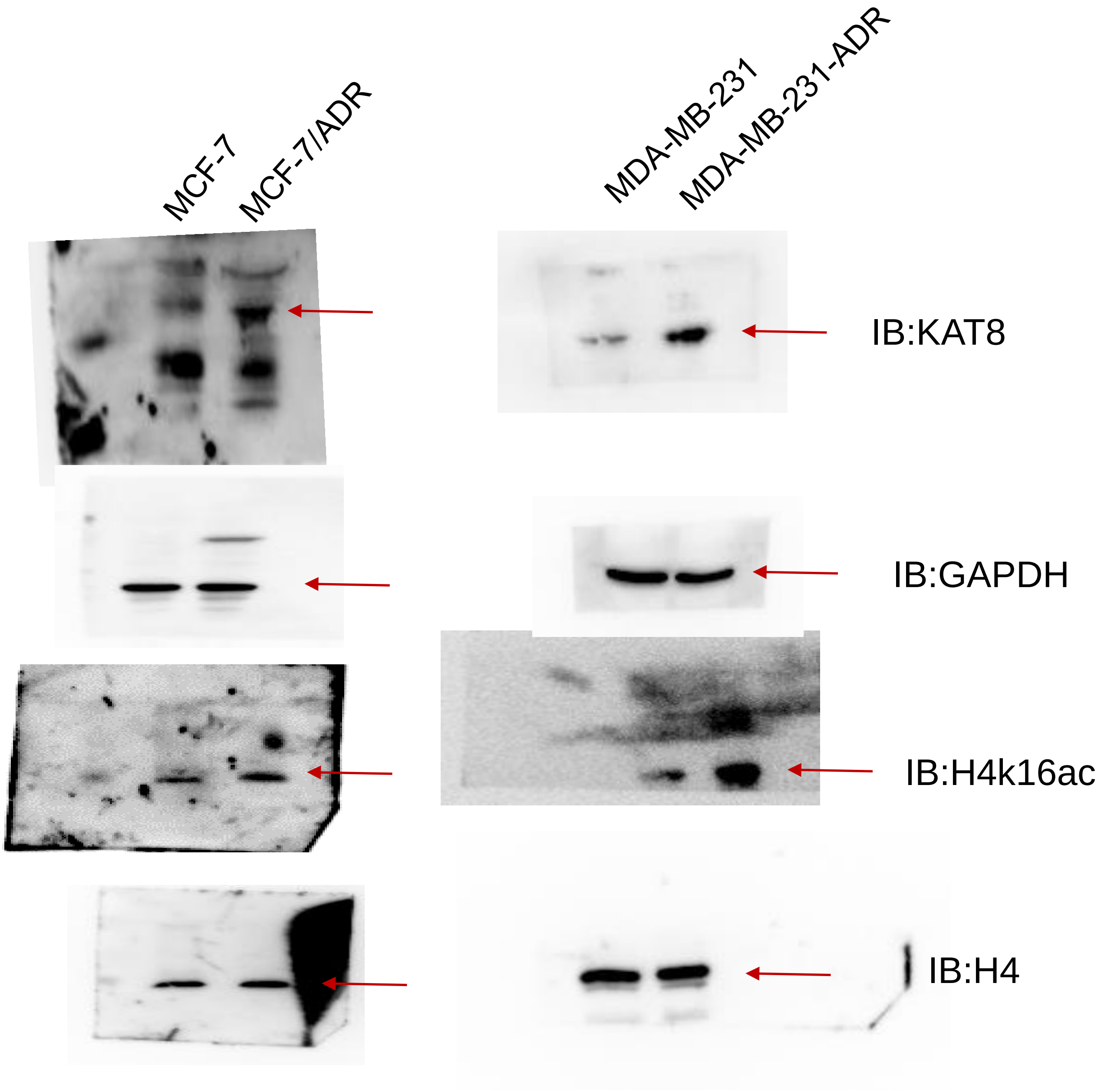
MCF-7
MCF-7/ADR
MDA-MB-231
MDA-MB-231-ADR
IB:KAT8
IB:GAPDH
IB:H4k16ac
IB:H4

Figure 2G

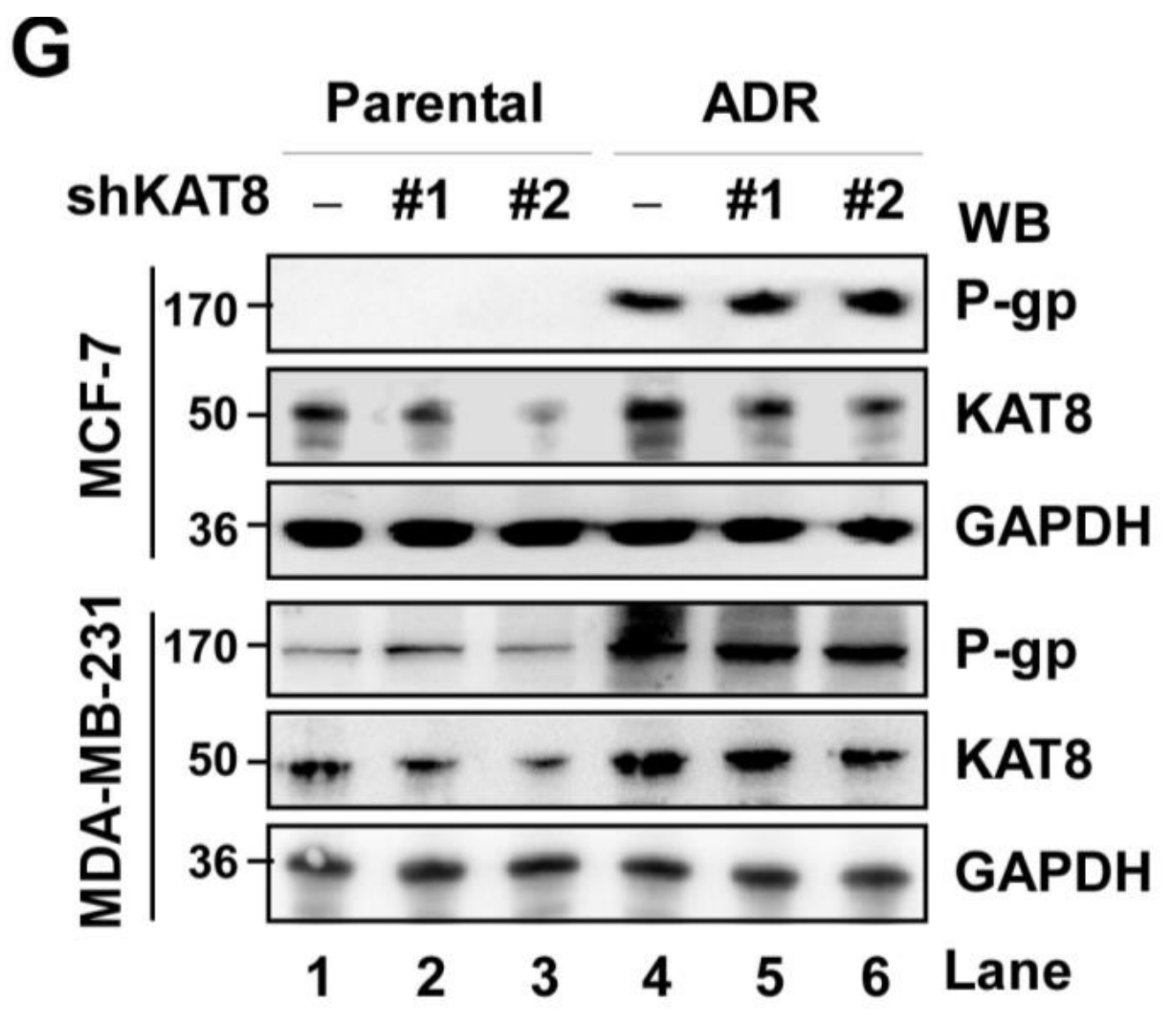
G
Parental
ADR
shKAT8
–
#1
#2
–
#1
#2
WB
MCF-7
MDA-MB-231
170
50
36
P-gp
KAT8
GAPDH
1
2
3
4
5
6
Lane

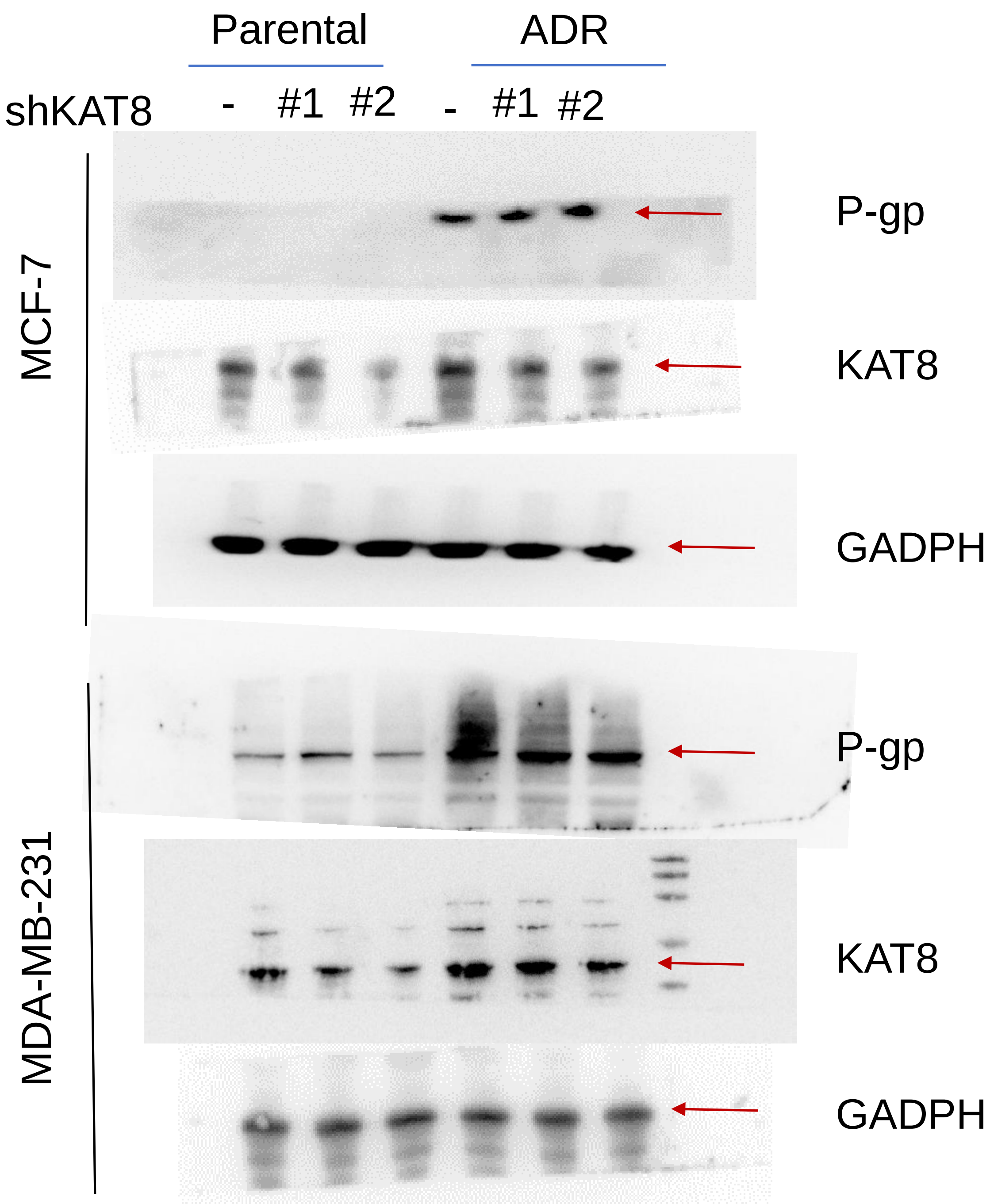
Parental
ADR
shKAT8
-
#1
#2
-
#1
#2
MCF-7
MDA-MB-231
P-gp
KAT8
GADPH

Figure 2H

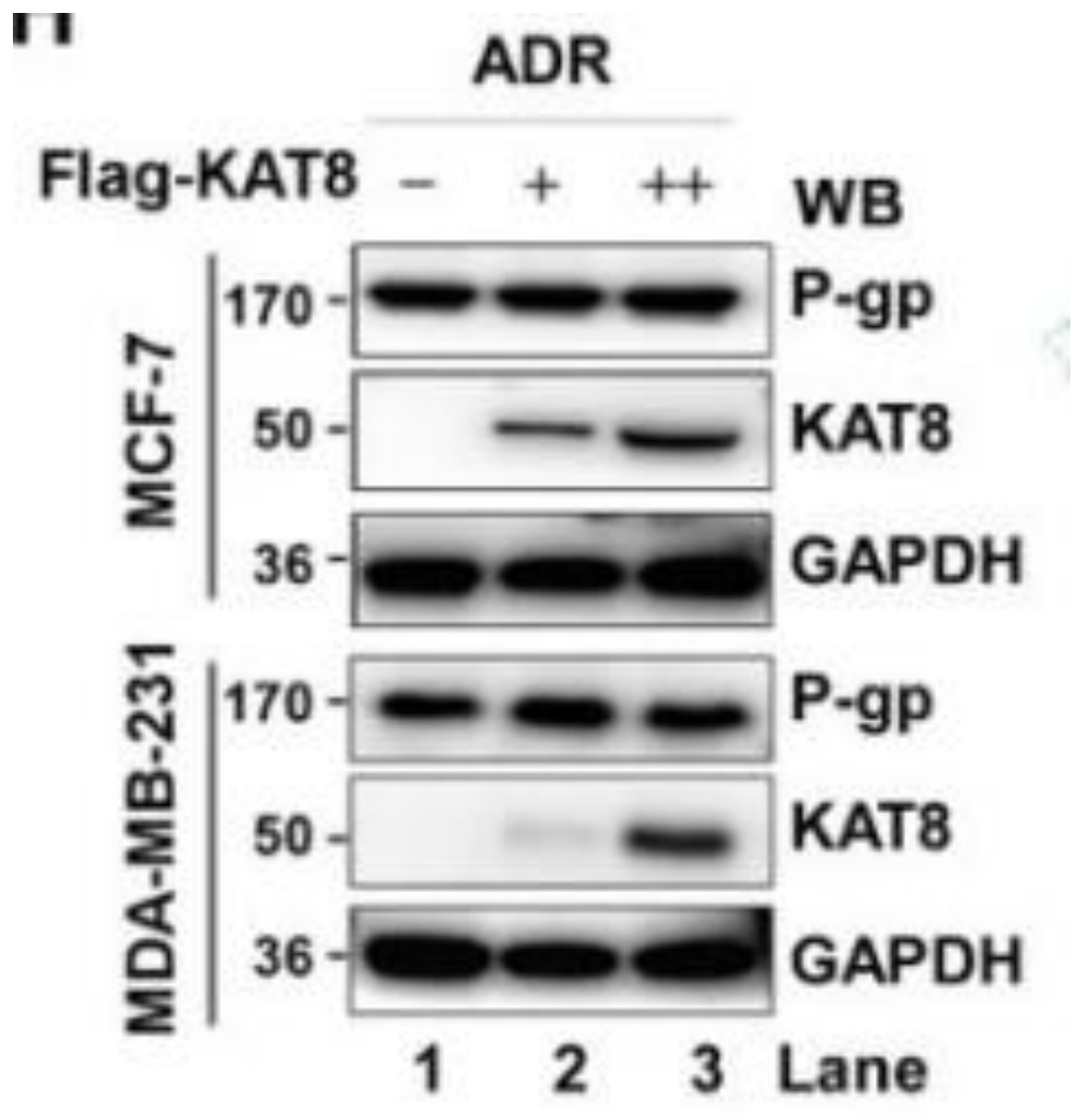

ADR
Flag-KAT8 − + ++
WB
MCF-7
170
50
36
P-gp
KAT8
GAPDH
MDA-MB-231
170
50
36
P-gp
KAT8
GAPDH
1 2 3 Lane

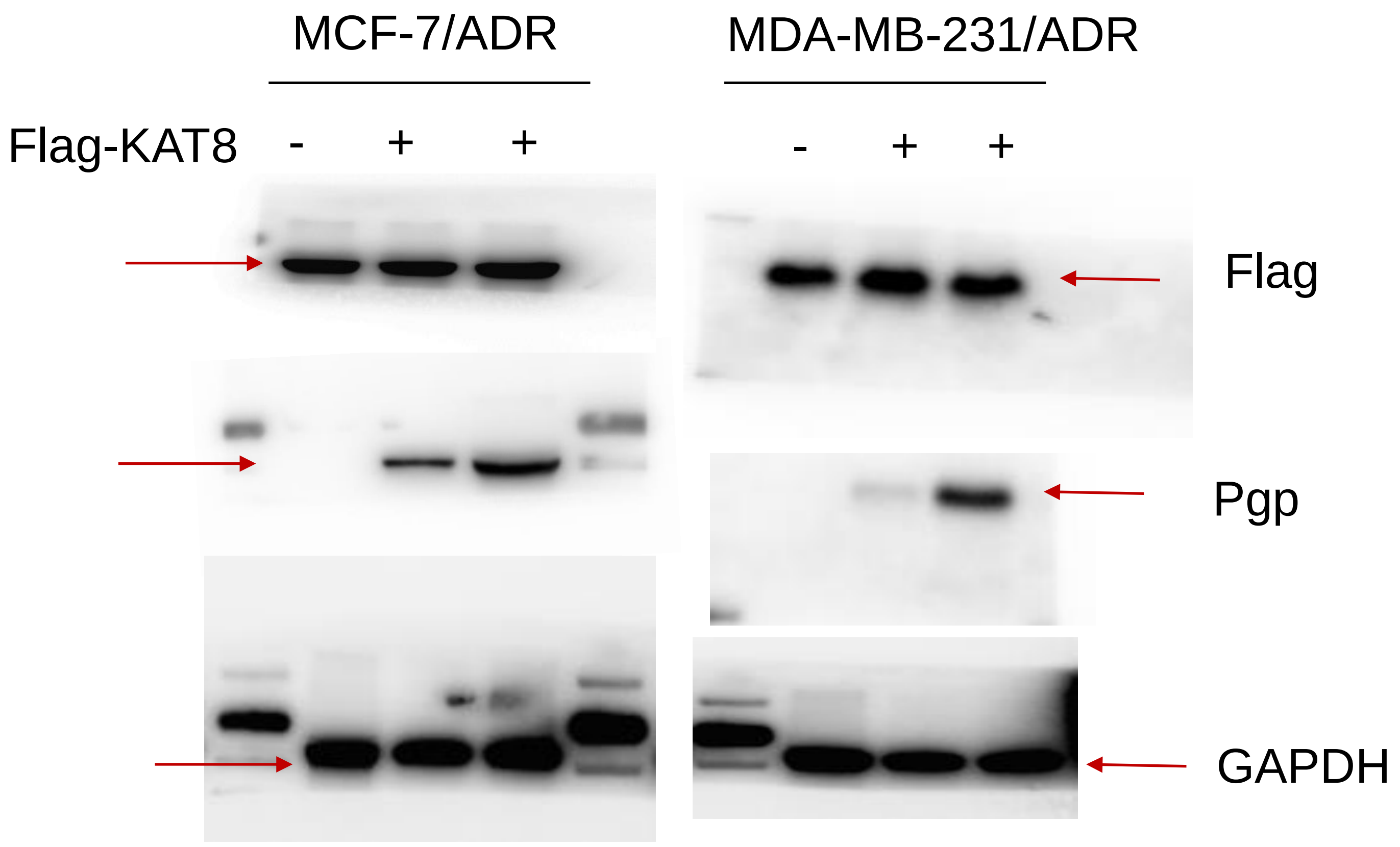

MCF-7/ADR
MDA-MB-231/ADR
Flag-KAT8 - + + - + +
Flag
Pgp
GAPDH

Figure 2I

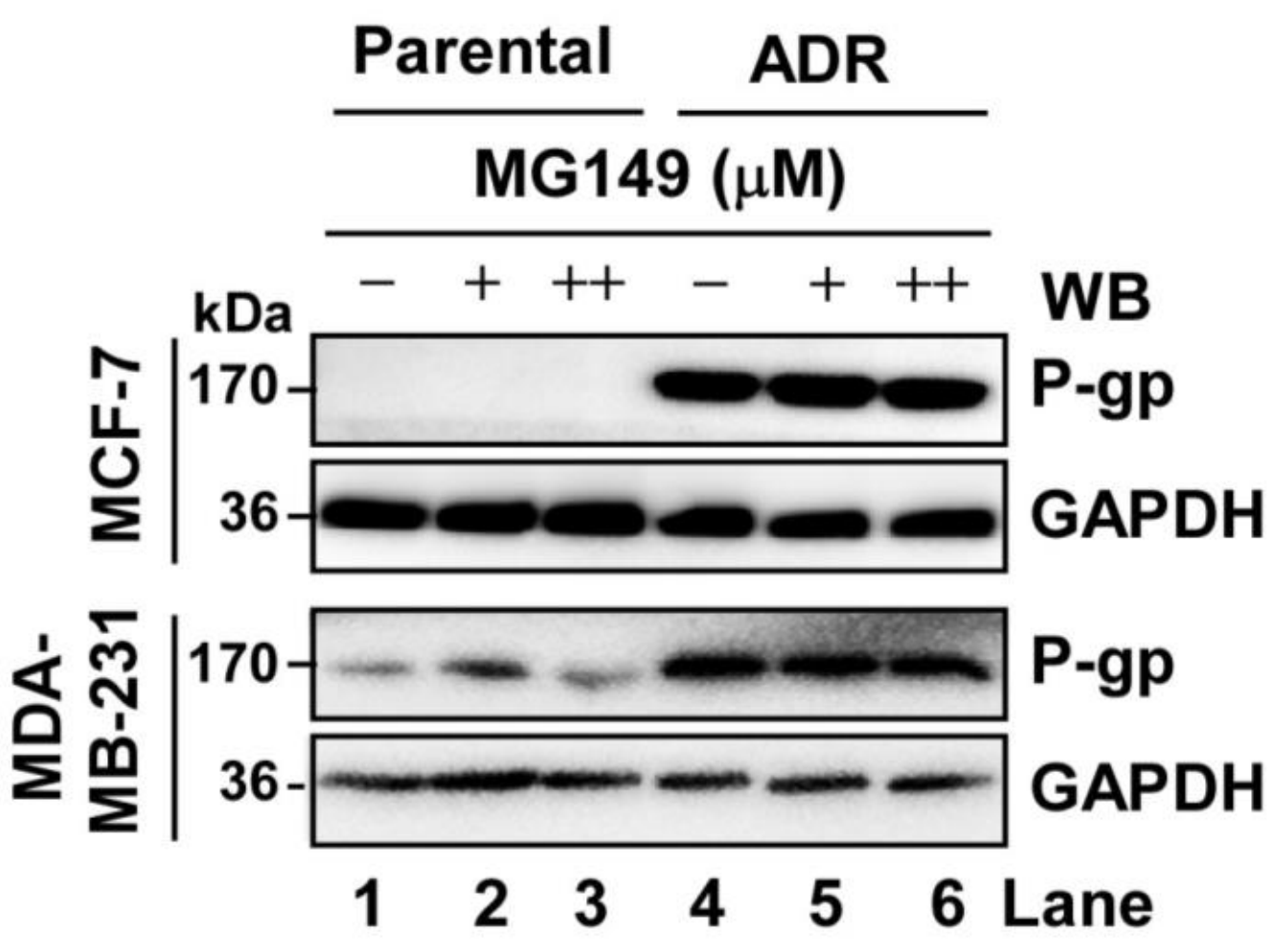

Parental
ADR
MG149 (μM)
– + ++ – + ++
WB
kDa
MCF-7
170
P-gp
36
GAPDH
MDA-MB-231
170
P-gp
36
GAPDH
1 2 3 4 5 6 Lane

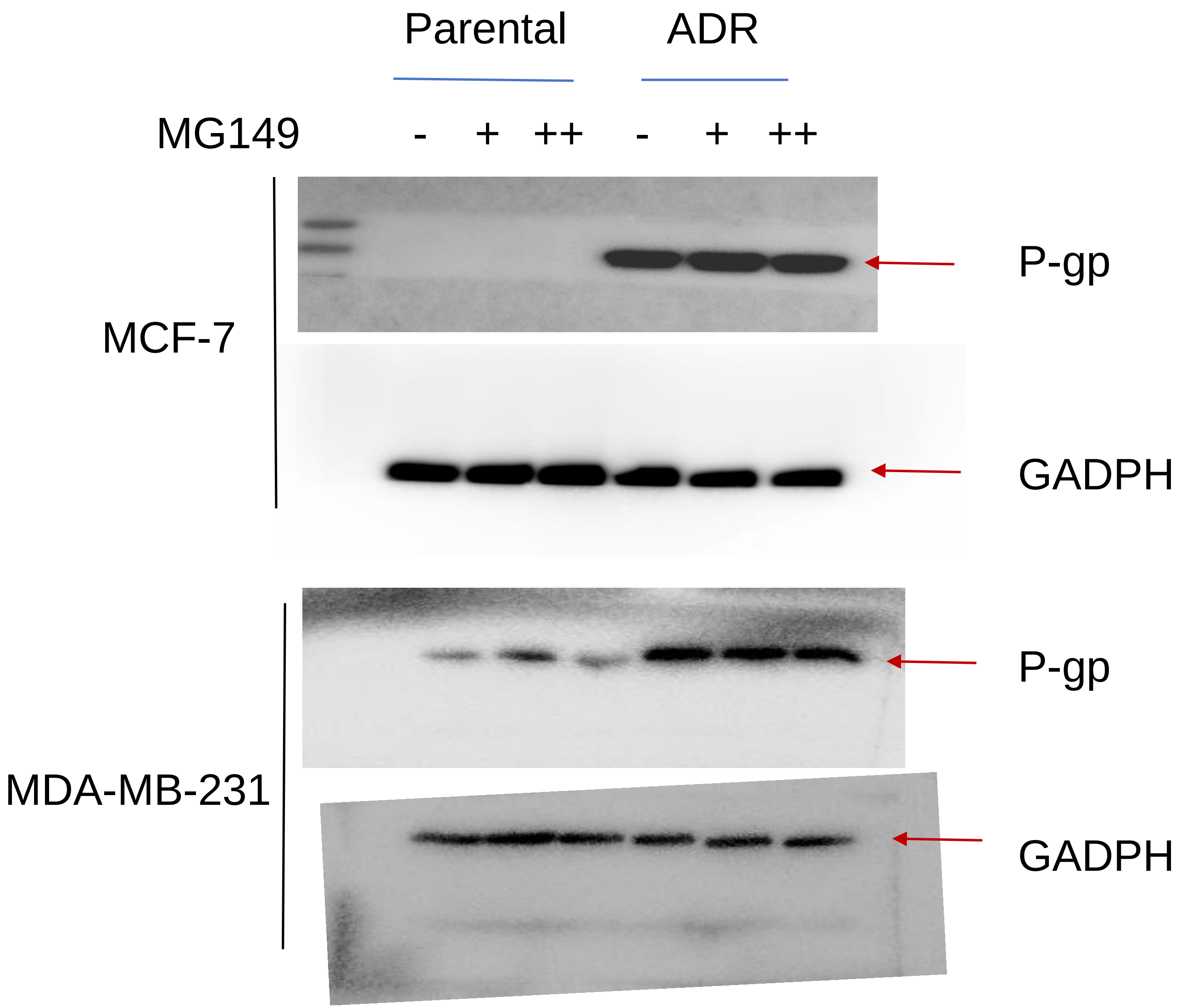

Parental
ADR
MG149
- + ++ - + ++
MCF-7
P-gp
GADPH
MDA-MB-231
P-gp
GADPH

Figure 2K

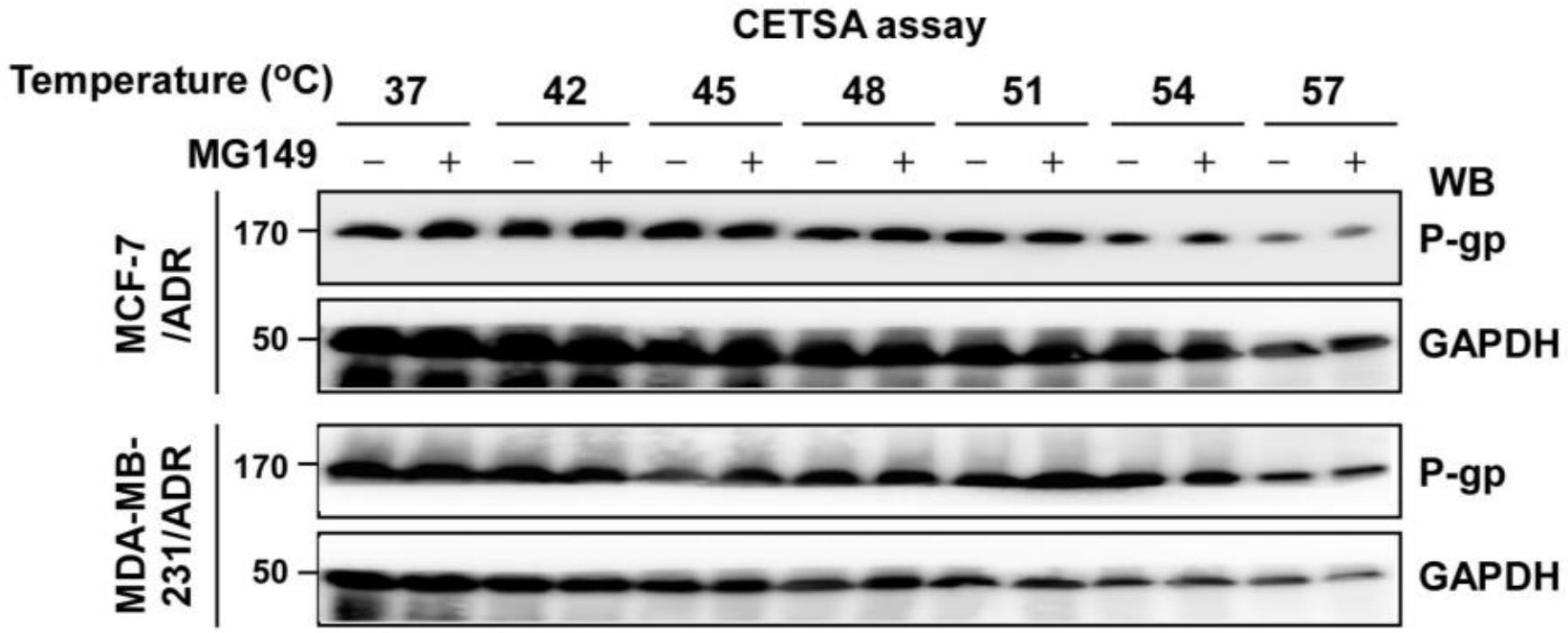
CETSA assay
Temperature (°C)
37
42
45
48
51
54
57
MG149
– + – + – + – + – + – + – +
WB
MCF-7
/ADR
170
50
P-gp
GAPDH
MDA-MB-
231/ADR
170
50
P-gp
GAPDH

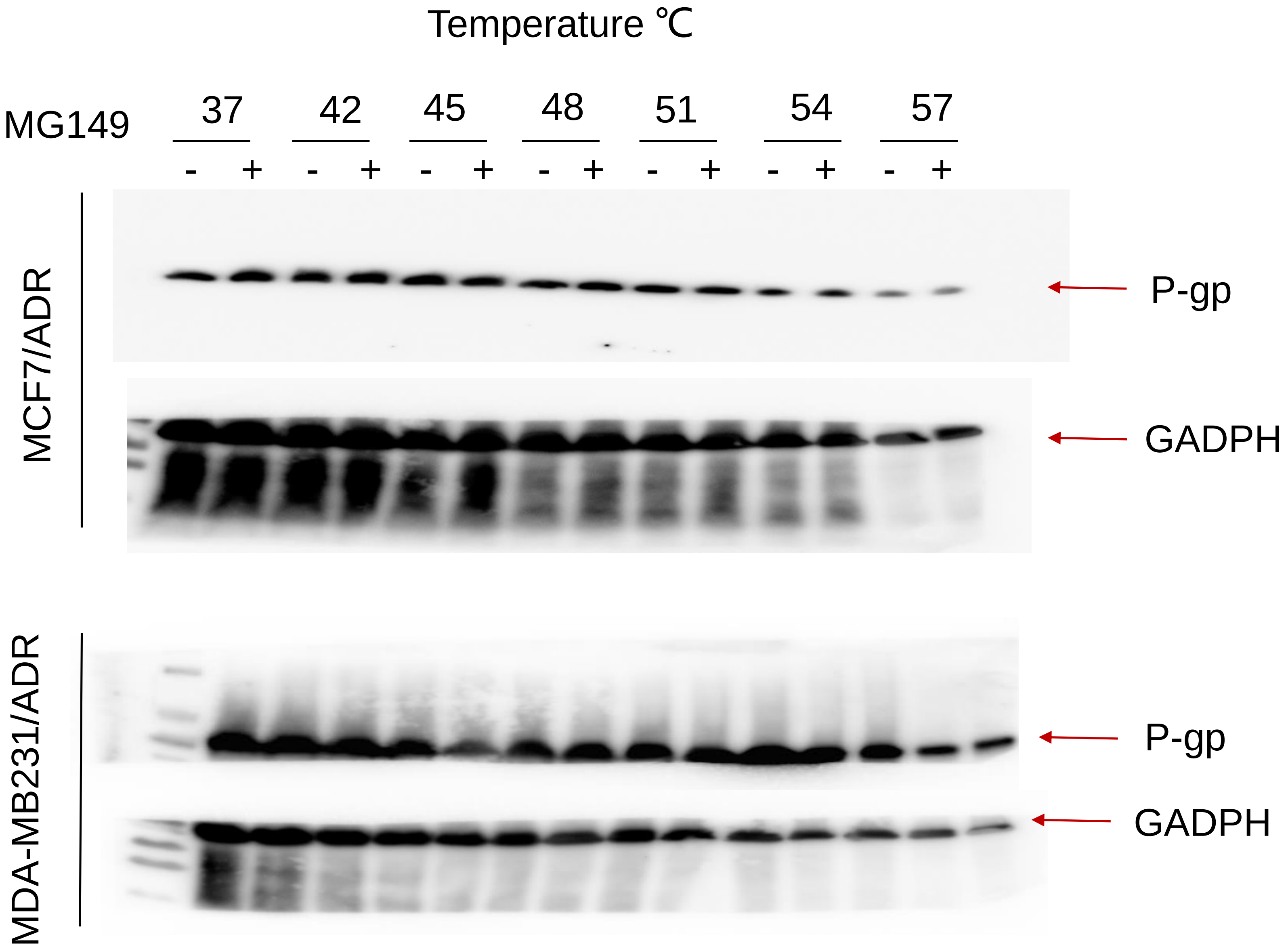
Temperature ℃
MG149
37
42
45
48
51
54
57
- + - + - + - + - + - + - +
MCF7/ADR
P-gp
GADPH
MDA-MB231/ADR
P-gp
GADPH

Figure 4A

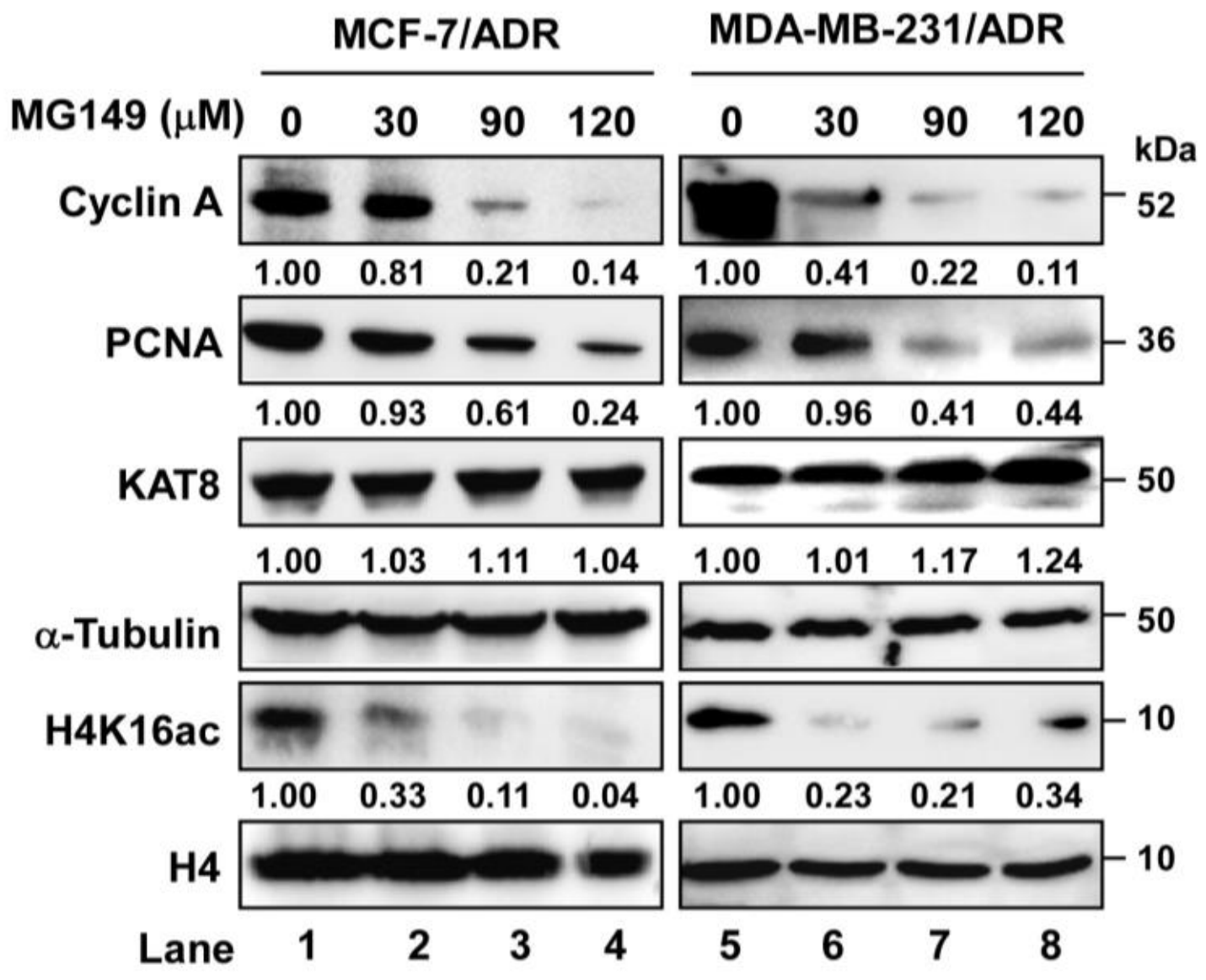
MCF-7/ADR
MDA-MB-231/ADR
MG149 (μM) 0 30 90 120 0 30 90 120
kDa
Cyclin A
52
1.00 0.81 0.21 0.14 1.00 0.41 0.22 0.11
PCNA
36
1.00 0.93 0.61 0.24 1.00 0.96 0.41 0.44
KAT8
50
1.00 1.03 1.11 1.04 1.00 1.01 1.17 1.24
α-Tubulin
50
H4K16ac
10
1.00 0.33 0.11 0.04 1.00 0.23 0.21 0.34
H4
10
Lane 1 2 3 4 5 6 7 8

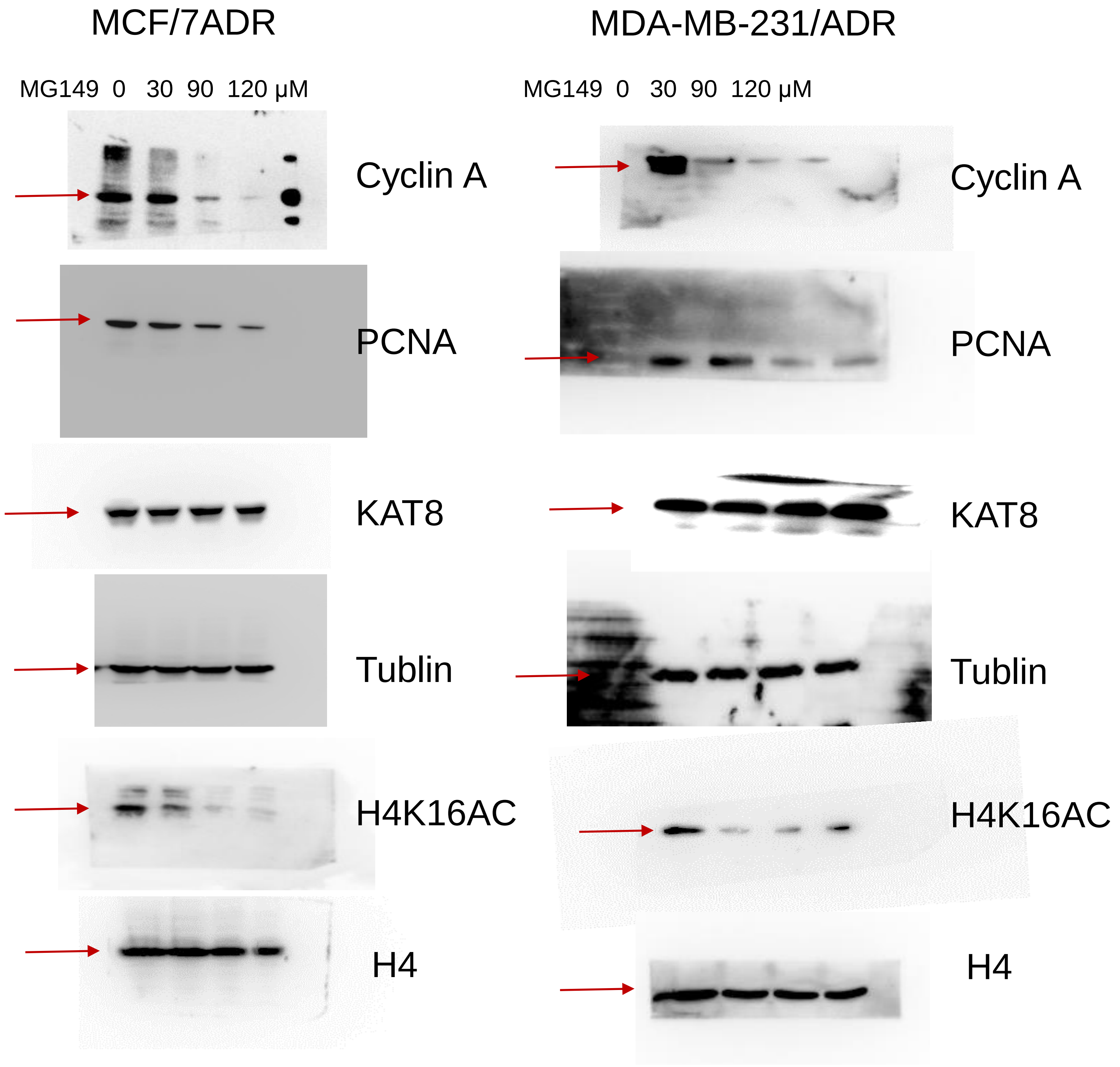
MCF/7ADR
MDA-MB-231/ADR
MG149 0 30 90 120 μM
MG149 0 30 90 120 μM
Cyclin A
Cyclin A
PCNA
PCNA
KAT8
KAT8
Tublin
Tublin
H4K16AC
H4K16AC
H4
H4

Figure 4E

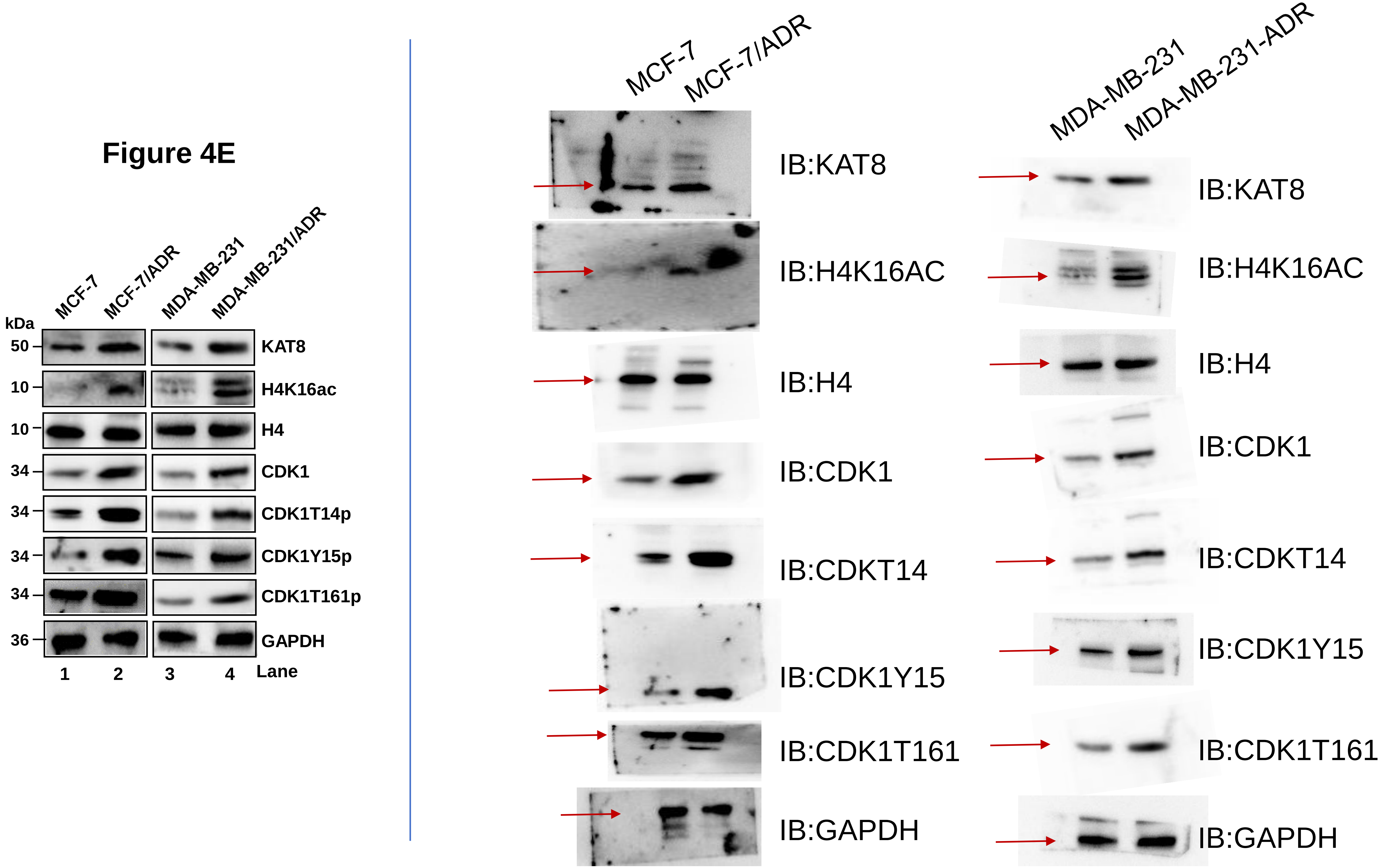

MCF-7
MCF-7/ADR
MDA-MB-231
MDA-MB-231/ADR
kDa
50
10
10
34
34
34
34
36
KAT8
H4K16ac
H4
CDK1
CDK1T14p
CDK1Y15p
CDK1T161p
GAPDH
1
2
3
4
Lane
MCF-7
MCF-7/ADR
IB:KAT8
IB:H4K16AC
IB:H4
IB:CDK1
IB:CDKT14
IB:CDK1Y15
IB:CDK1T161
IB:GAPDH
MDA-MB-231
MDA-MB-231-ADR
IB:KAT8
IB:H4K16AC
IB:H4
IB:CDK1
IB:CDKT14
IB:CDK1Y15
IB:CDK1T161
IB:GAPDH

Figure 4F, left panel

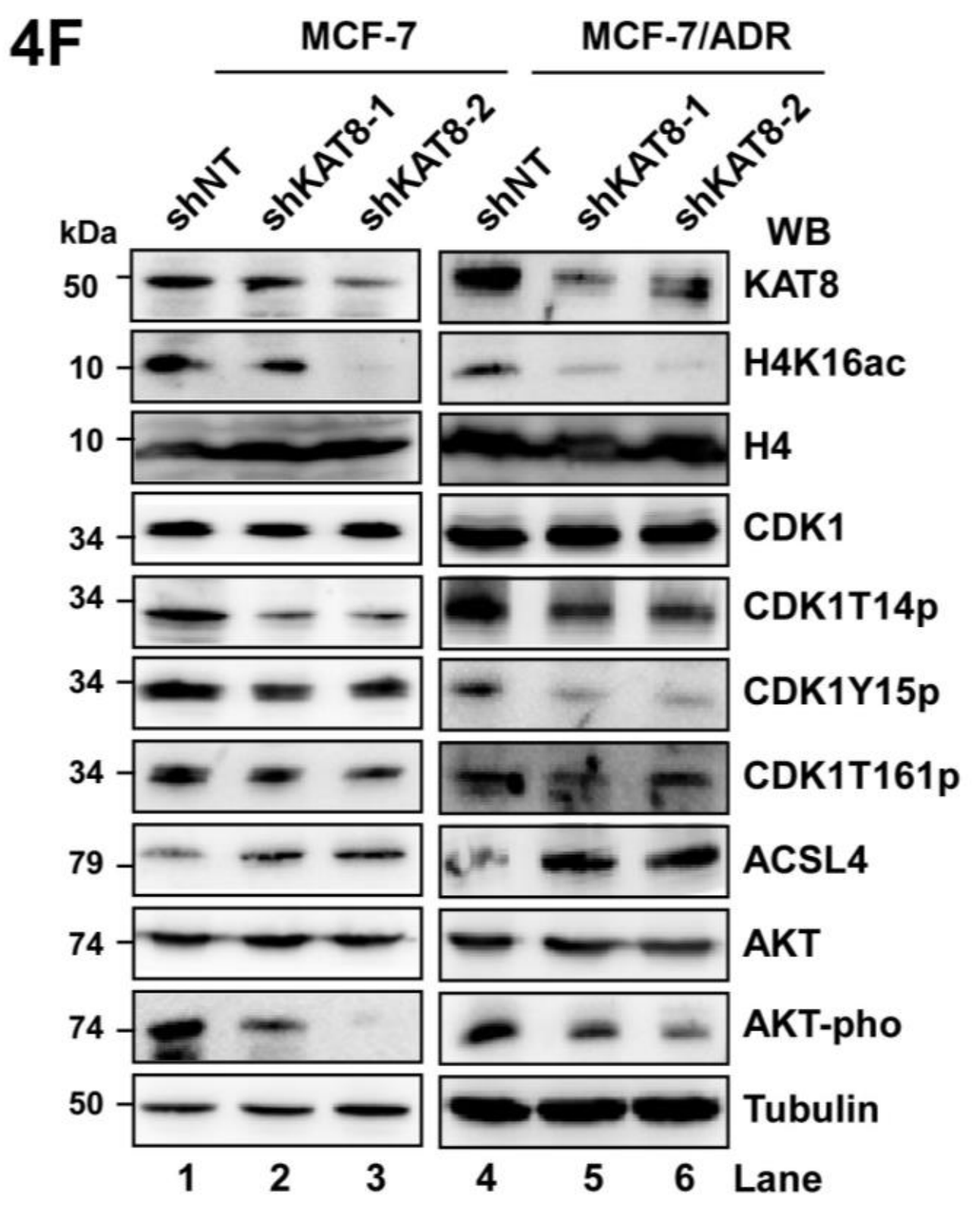

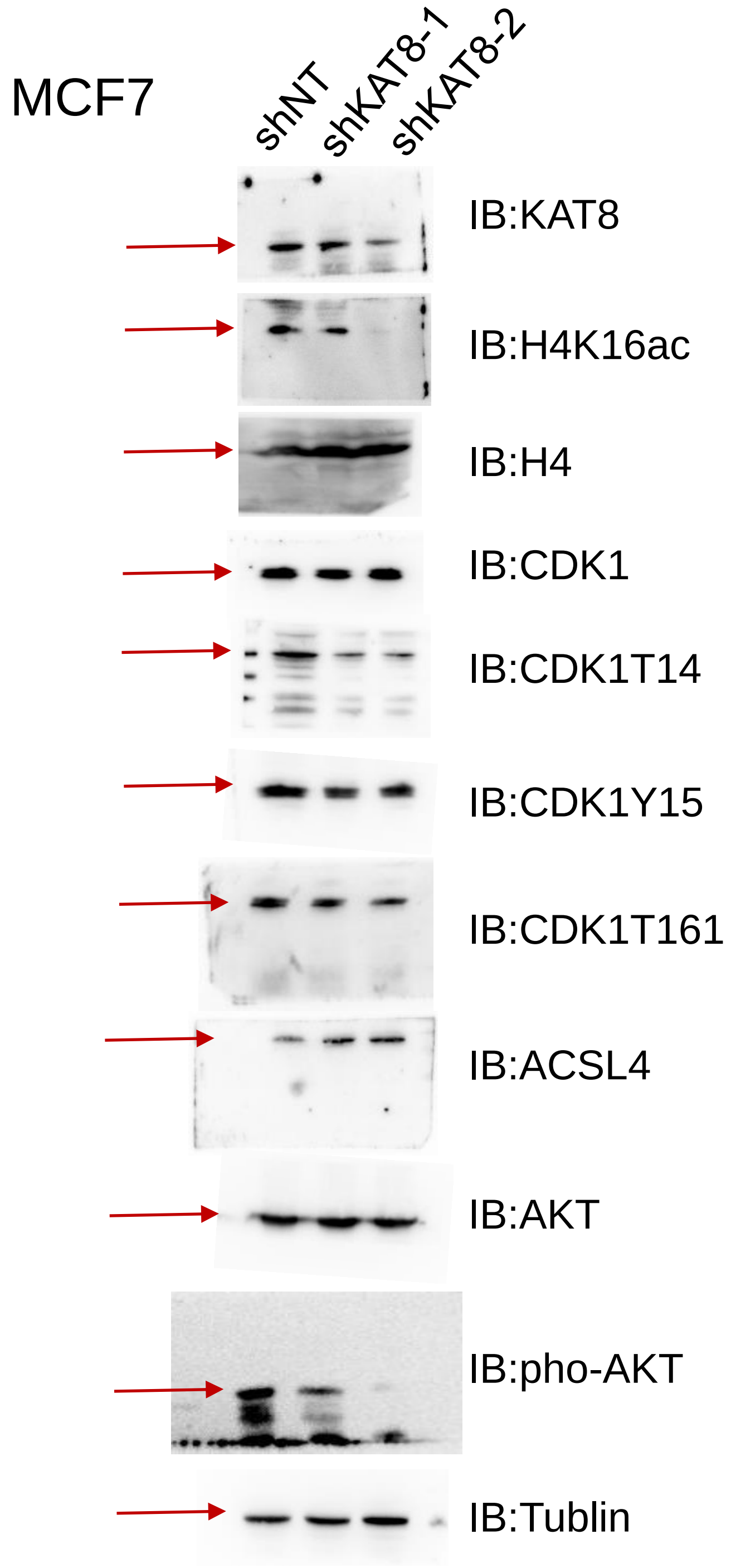

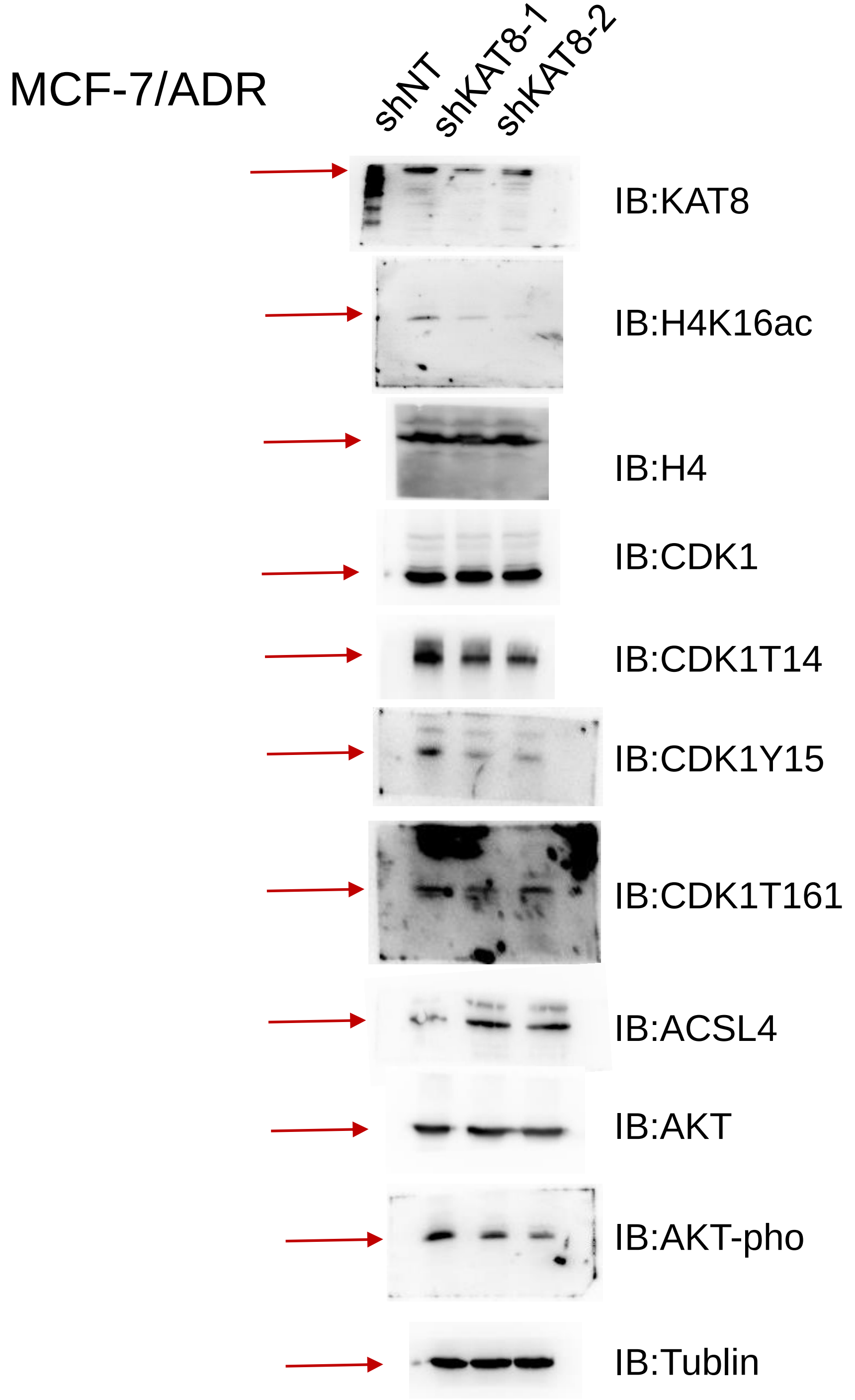

Figure 4F, right panel

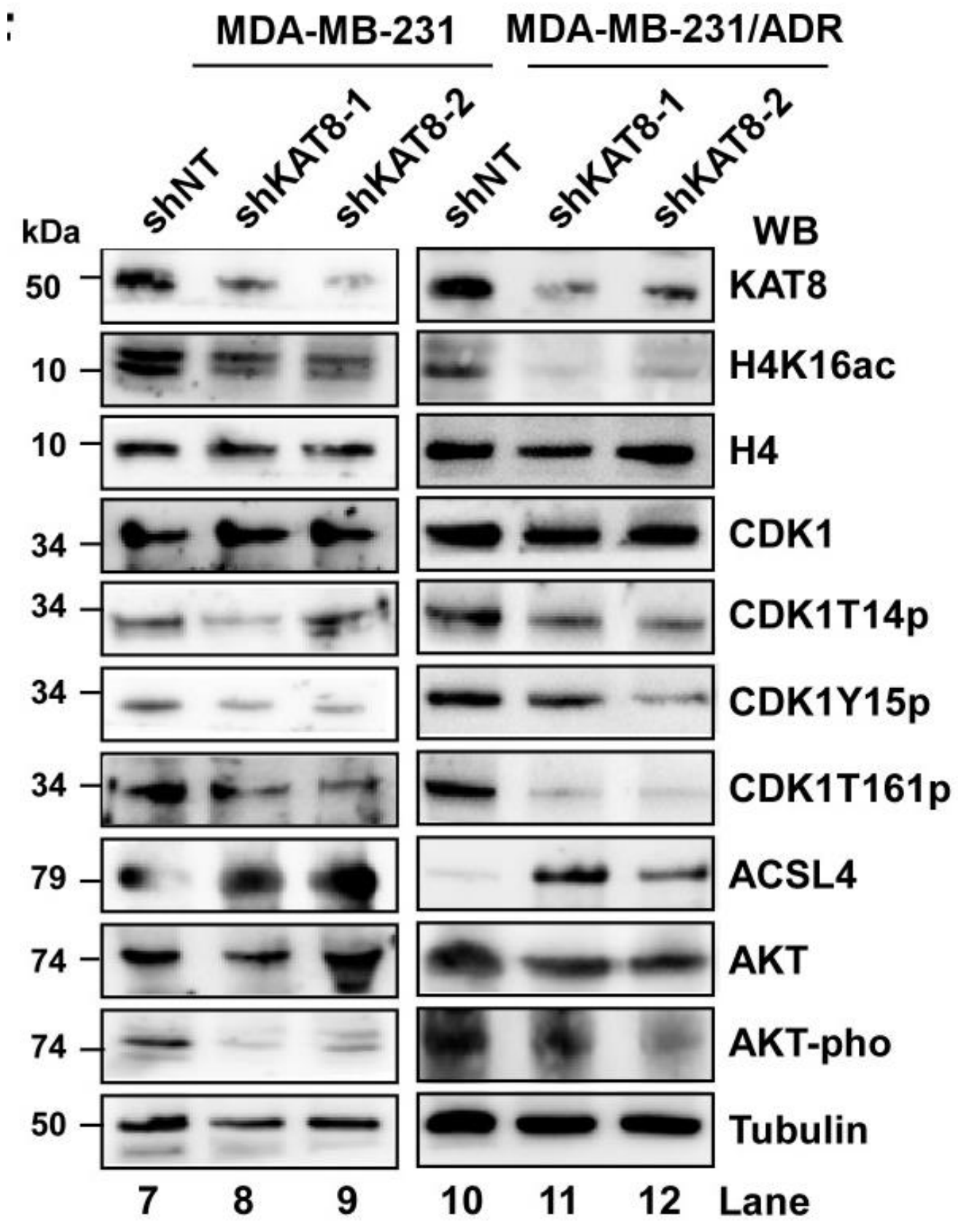

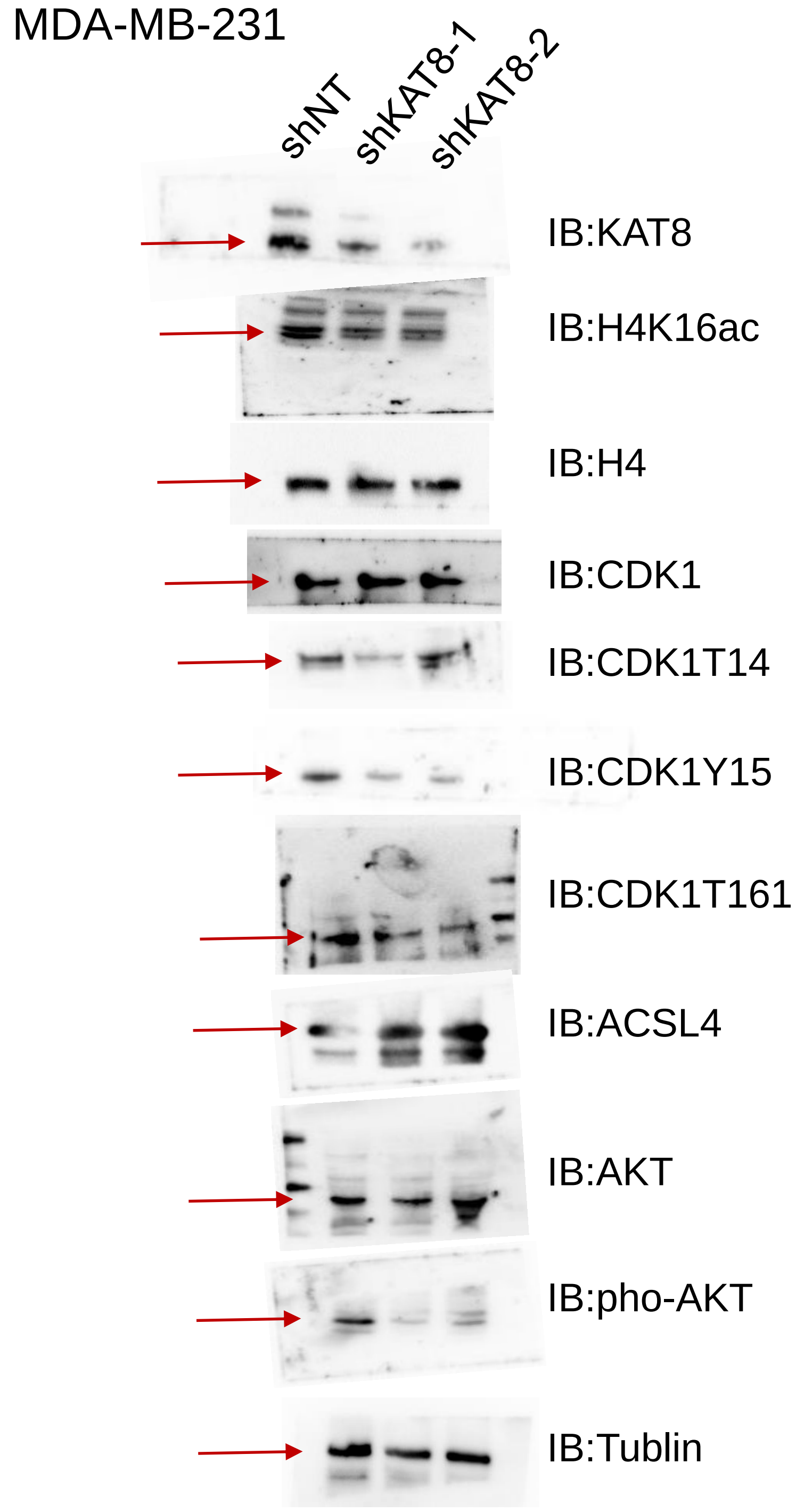

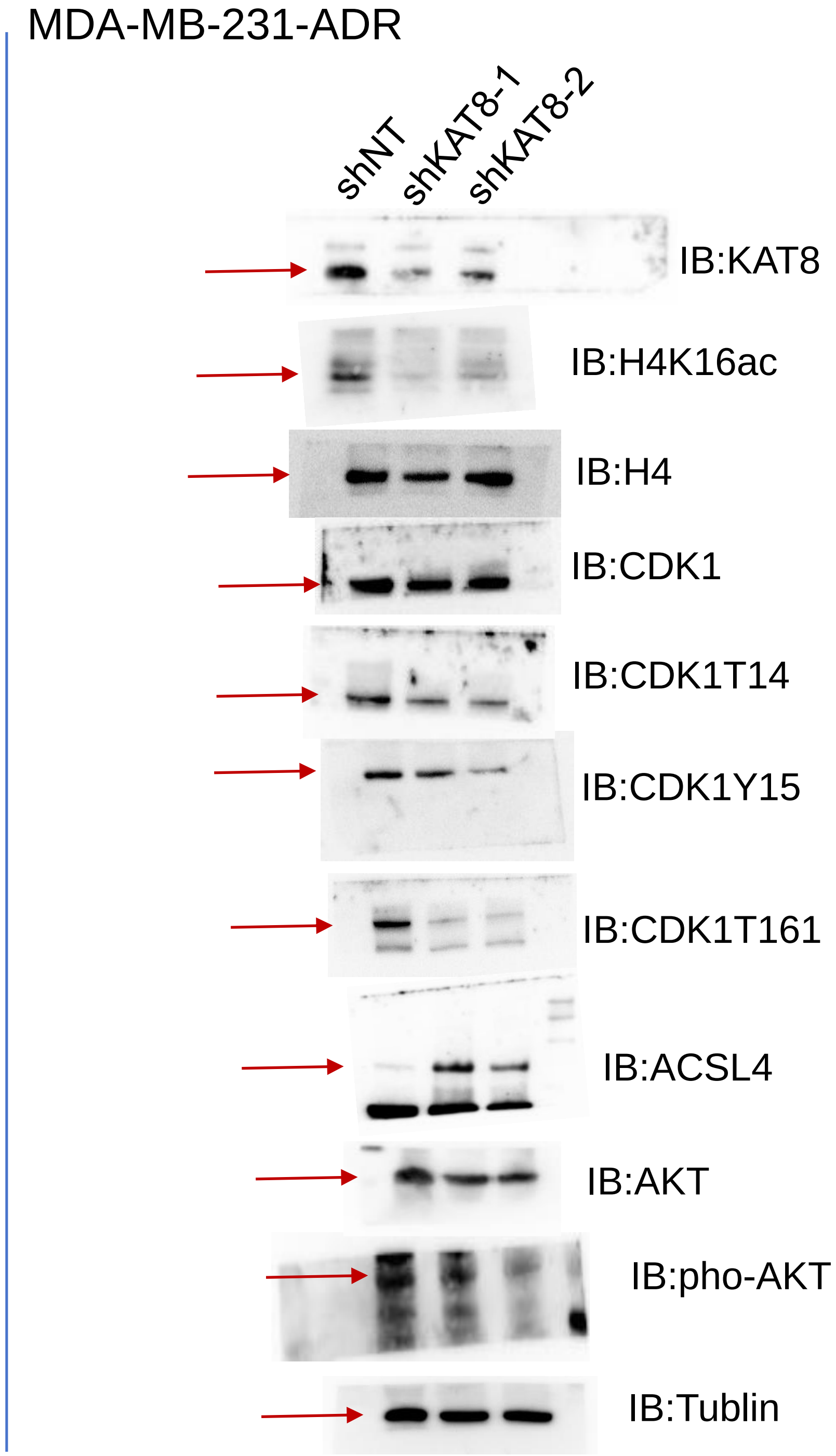

Figure 4G

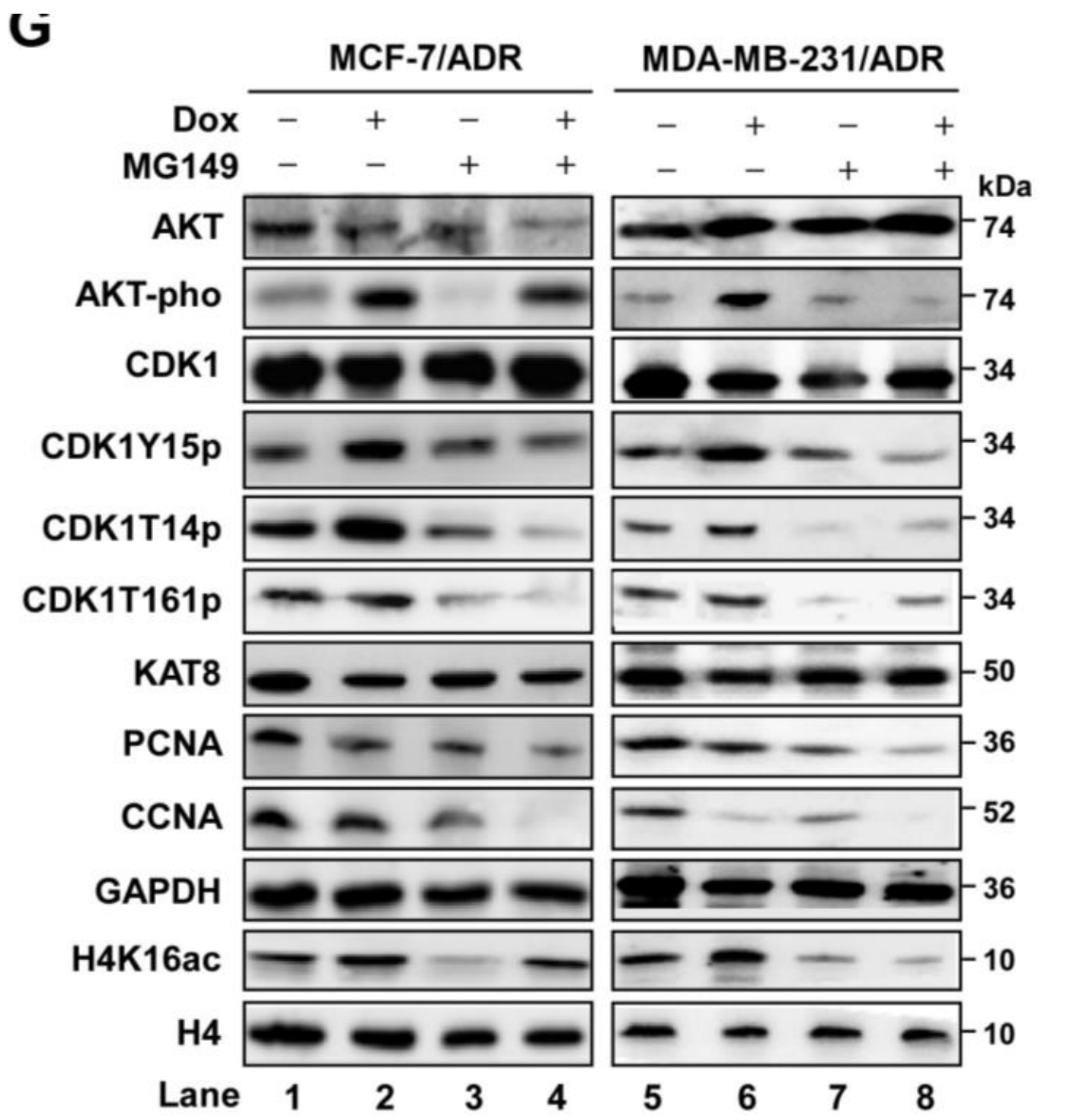

MCF-7/ADR

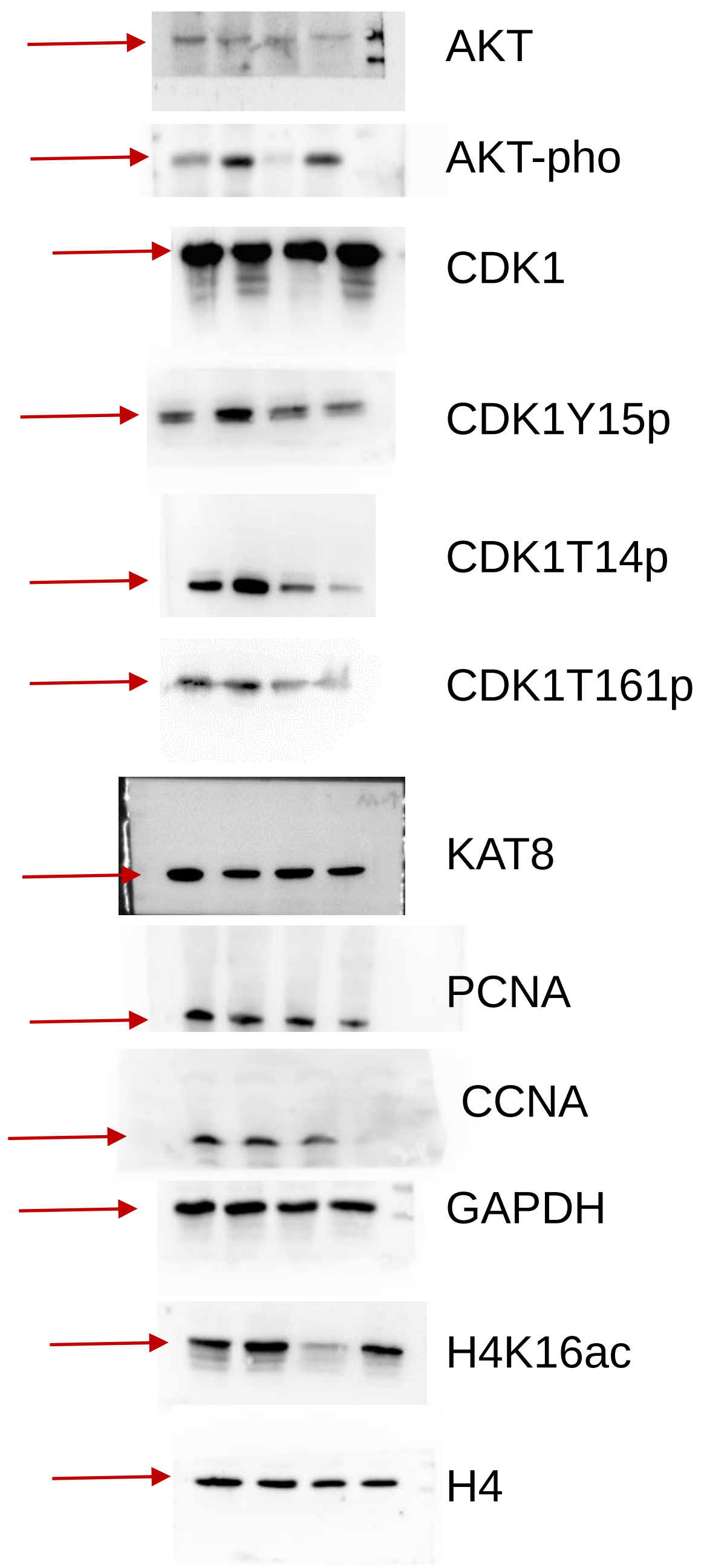

MDA-MB-231/ADR

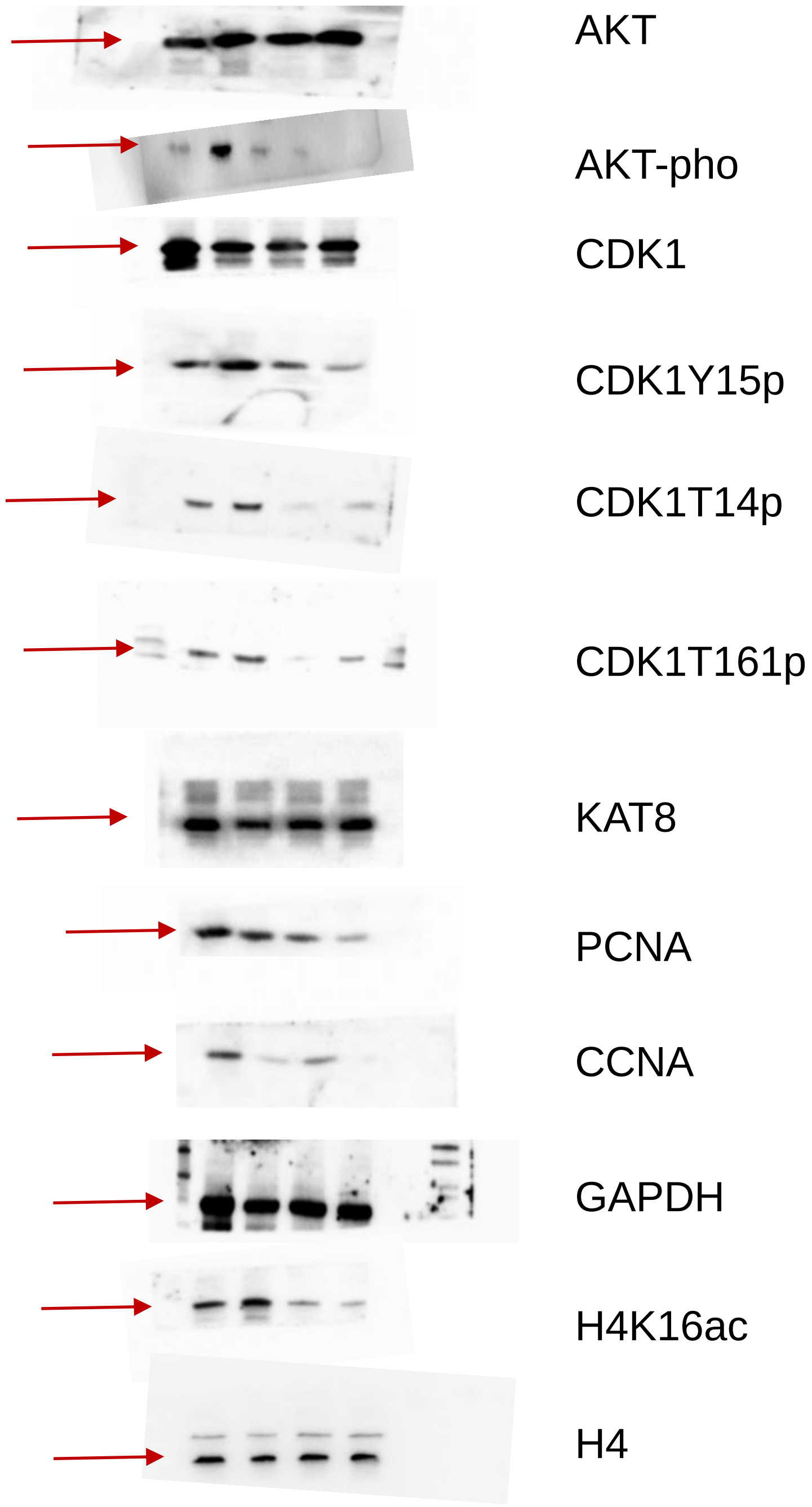

Figure 5C

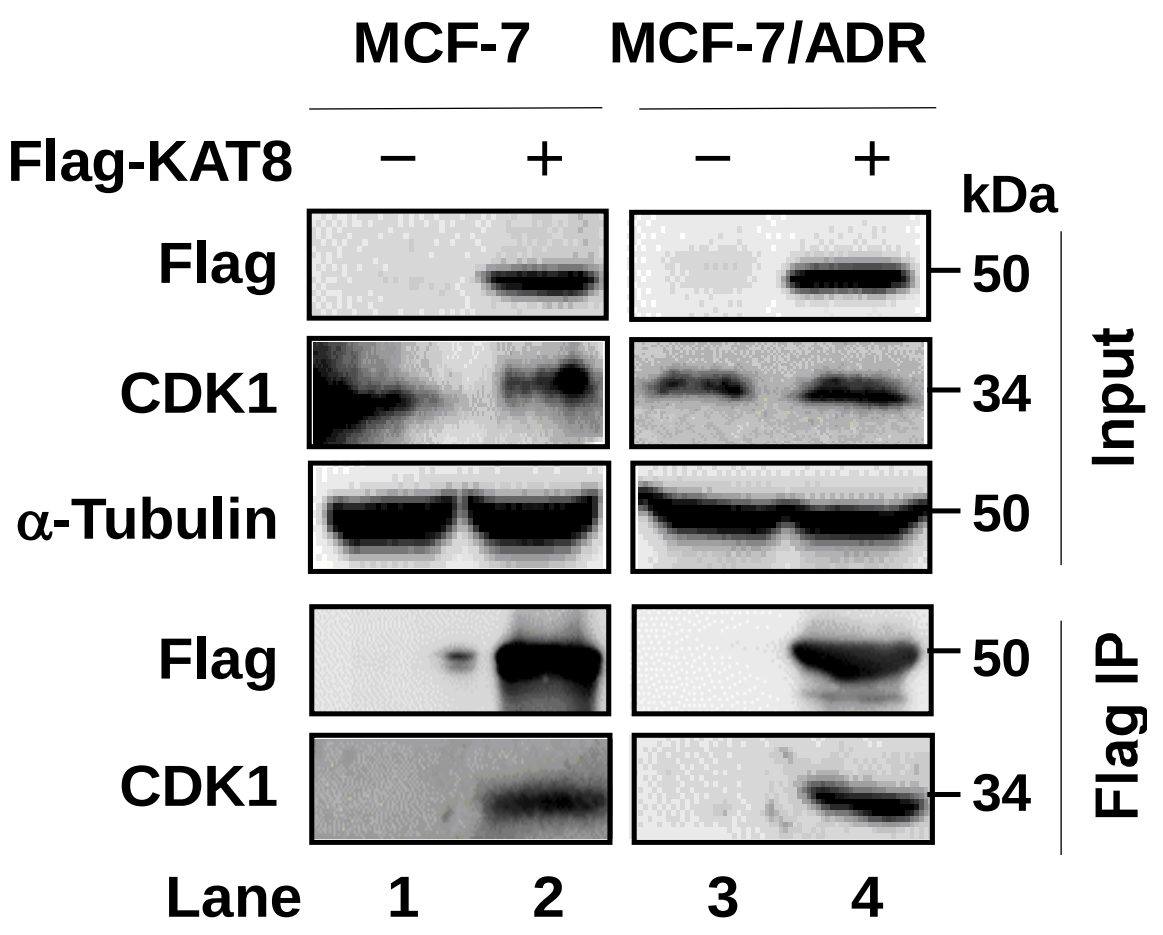

MCF-7
MCF-7/ADR
Flag-KAT8
−
+
−
+
kDa
Flag
CDK1
α-Tubulin
Flag
CDK1
50
34
50
50
34
Input
Flag IP
Lane
1
2
3
4

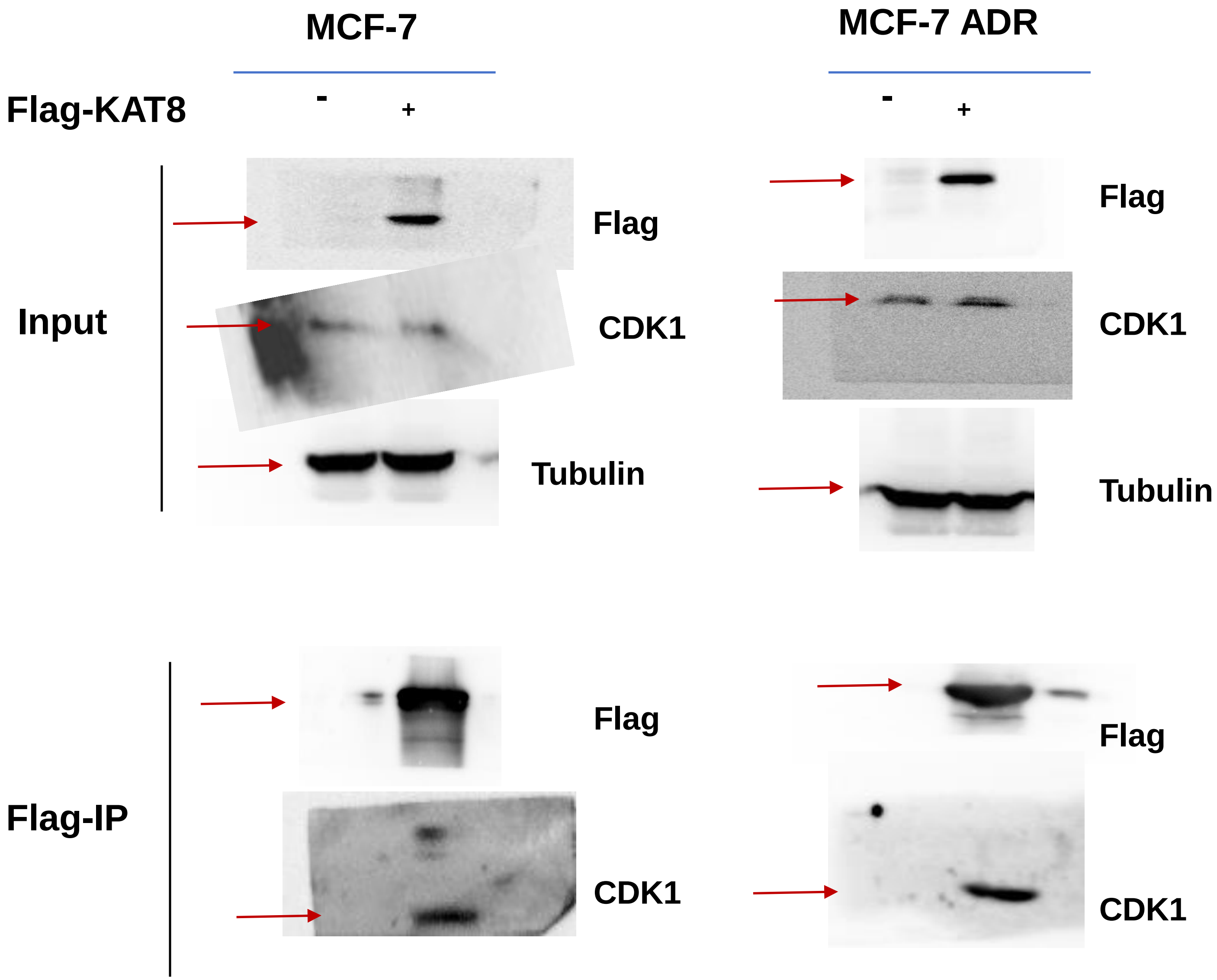

MCF-7
MCF-7 ADR
Flag-KAT8
-
+
-
+
Input
Flag
CDK1
Tubulin
Flag
CDK1
Tubulin
Flag-IP
Flag
CDK1
Flag
CDK1

Figure 5D

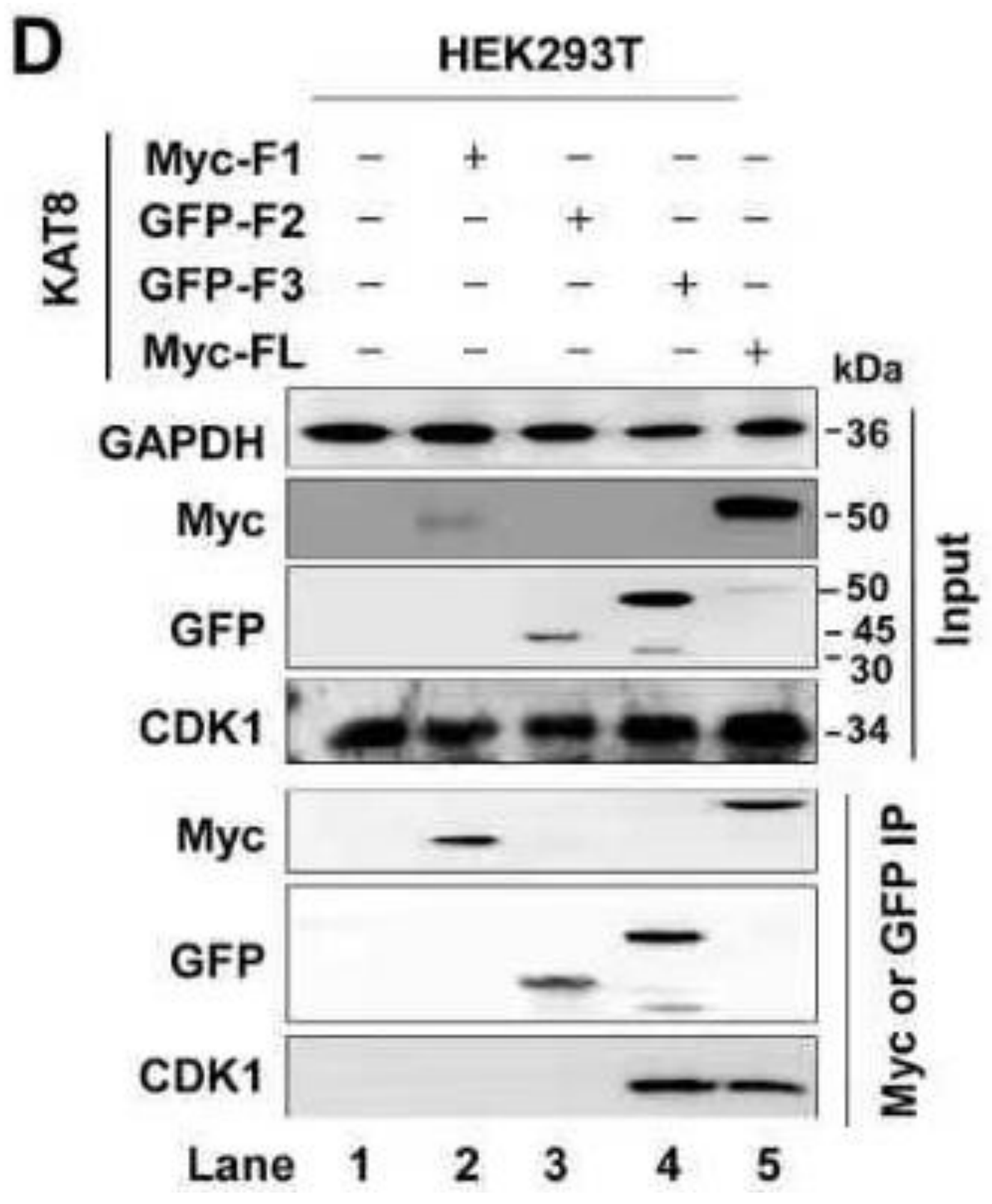
D
HEK293T
KAT8
Myc-F1 − + − − −
GFP-F2 − − + − −
GFP-F3 − − − + −
Myc-FL − − − − +
kDa
GAPDH
Myc
GFP
CDK1
-36
-50
-50
-45
-30
-34
Input
Myc
GFP
CDK1
Myc or GFP IP
Lane 1 2 3 4 5

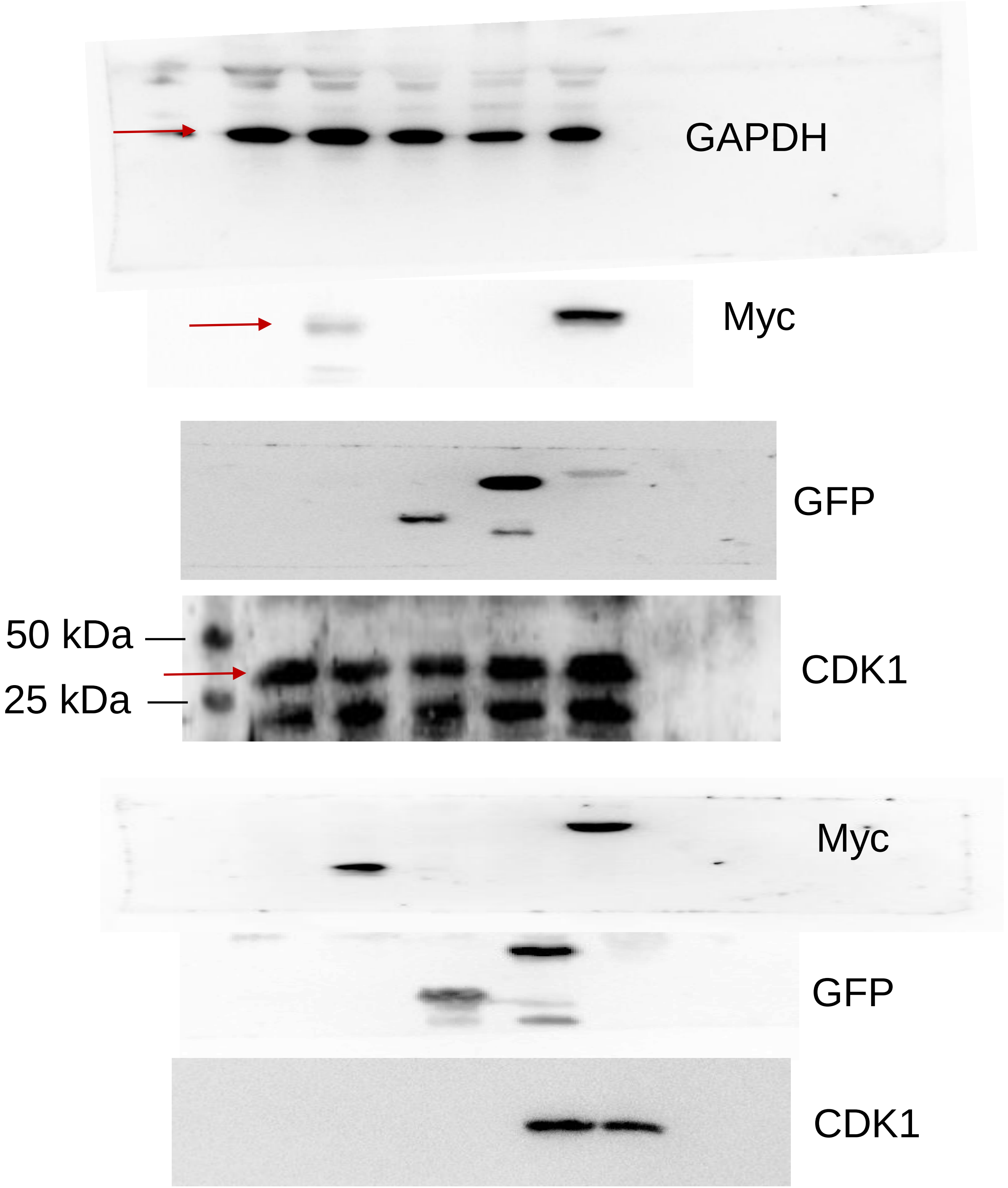
GAPDH
Myc
GFP
50 kDa
25 kDa
CDK1
Myc
GFP
CDK1

Figure 5E

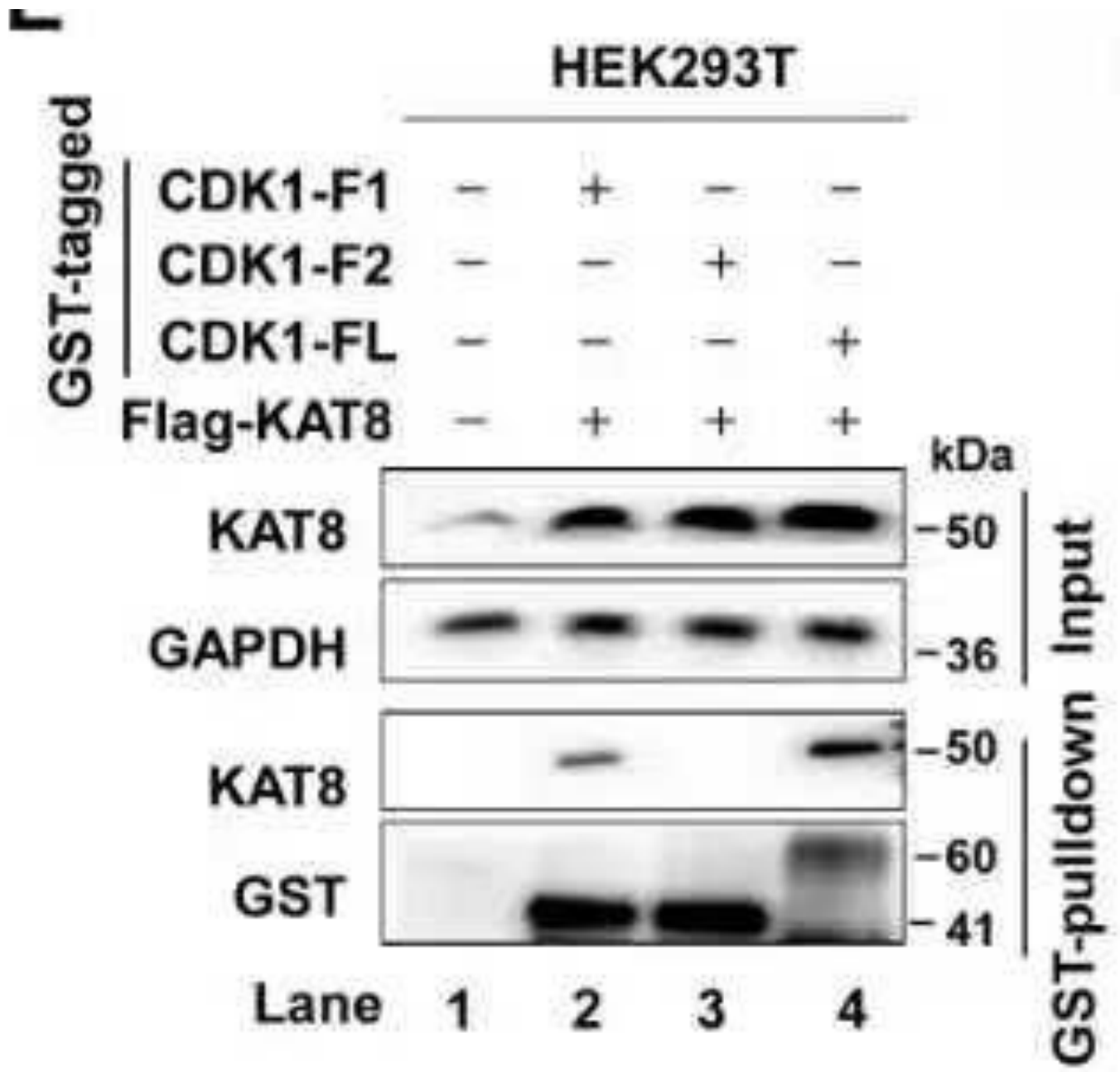
HEK293T
GST-tagged
CDK1-F1 − + − −
CDK1-F2 − − + −
CDK1-FL − − − +
Flag-KAT8 − + + +
kDa
KAT8
−50
GAPDH
−36
Input
KAT8
−50
GST
−60
−41
GST-pulldown
Lane 1 2 3 4

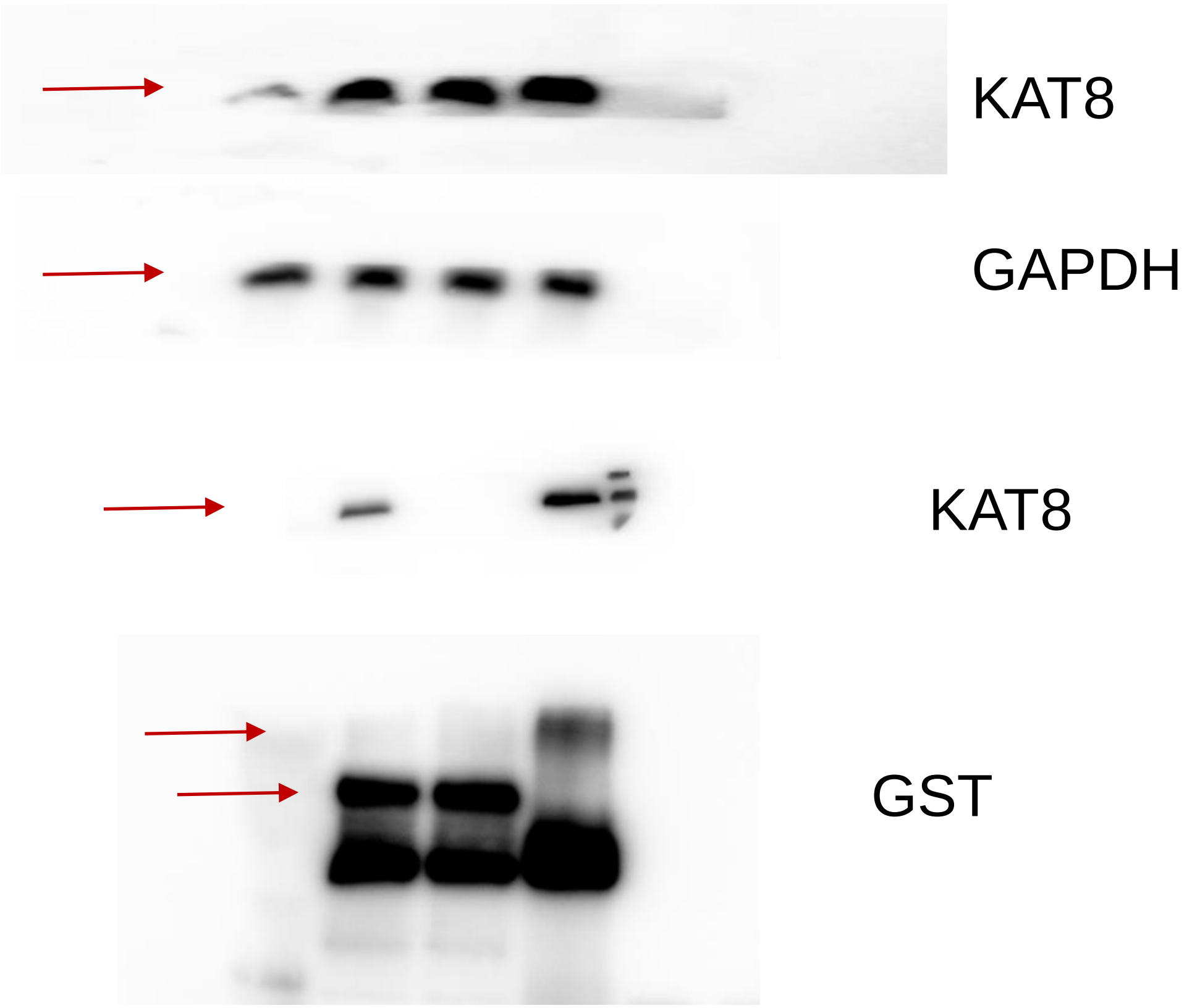
KAT8
GAPDH
KAT8
GST

Figure 5F

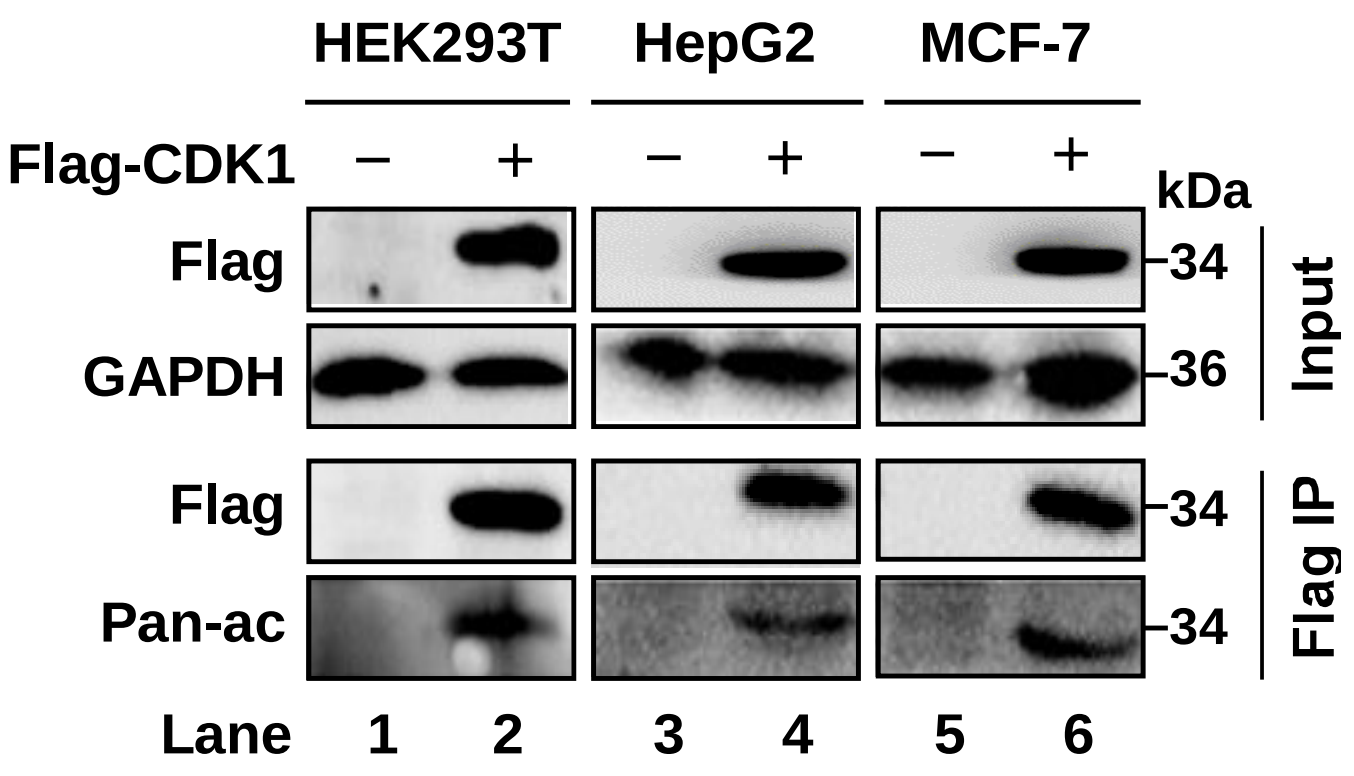

HEK293T
HepG2
MCF-7
Flag-CDK1 − + − + − +
kDa
Flag
34
GAPDH
36
Input
Flag
34
Pan-ac
34
Flag IP
Lane 1 2 3 4 5 6

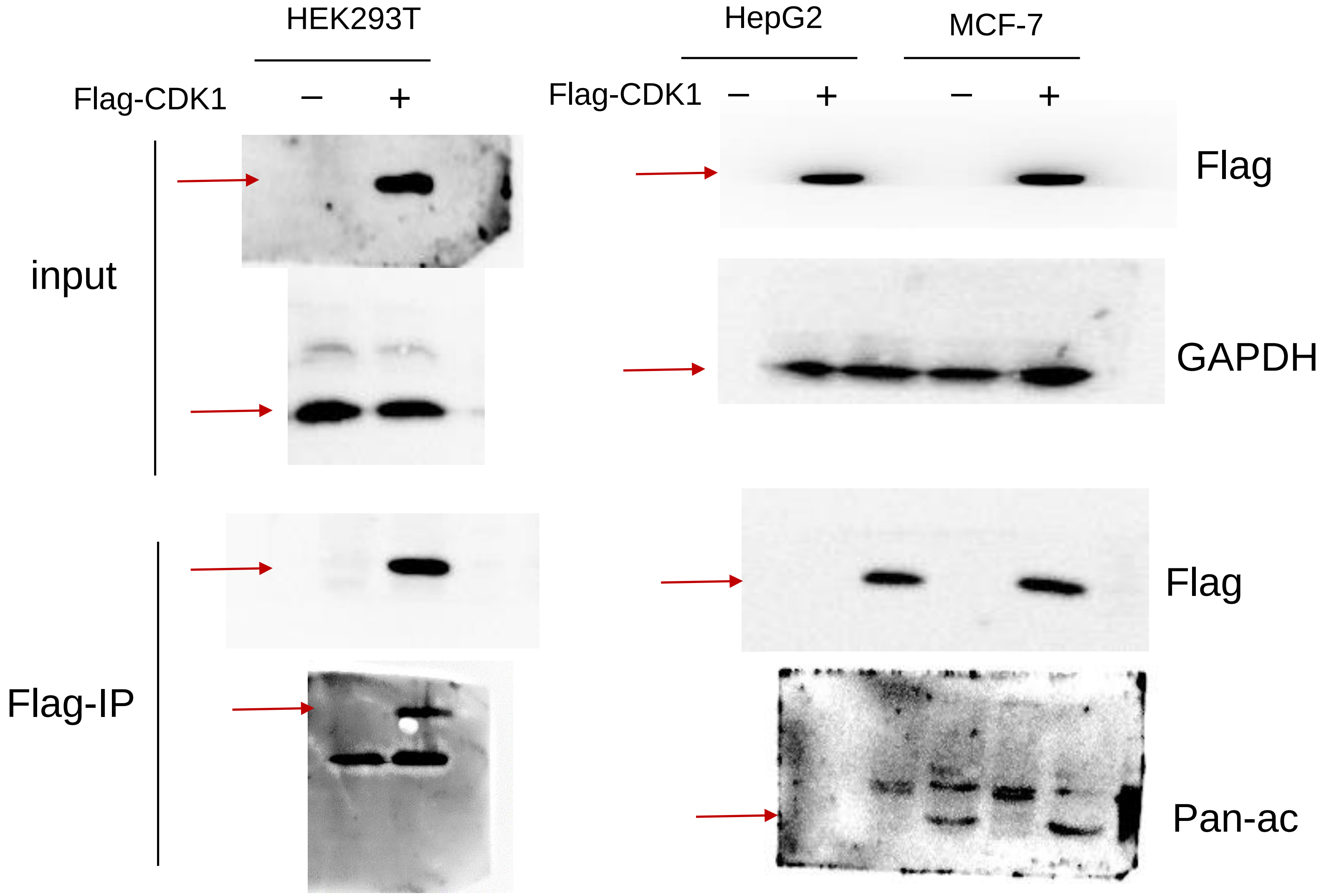

HEK293T
HepG2
MCF-7
Flag-CDK1 − +
Flag-CDK1 − + − +
input
Flag
GAPDH
Flag-IP
Flag
Pan-ac

Figure 5G

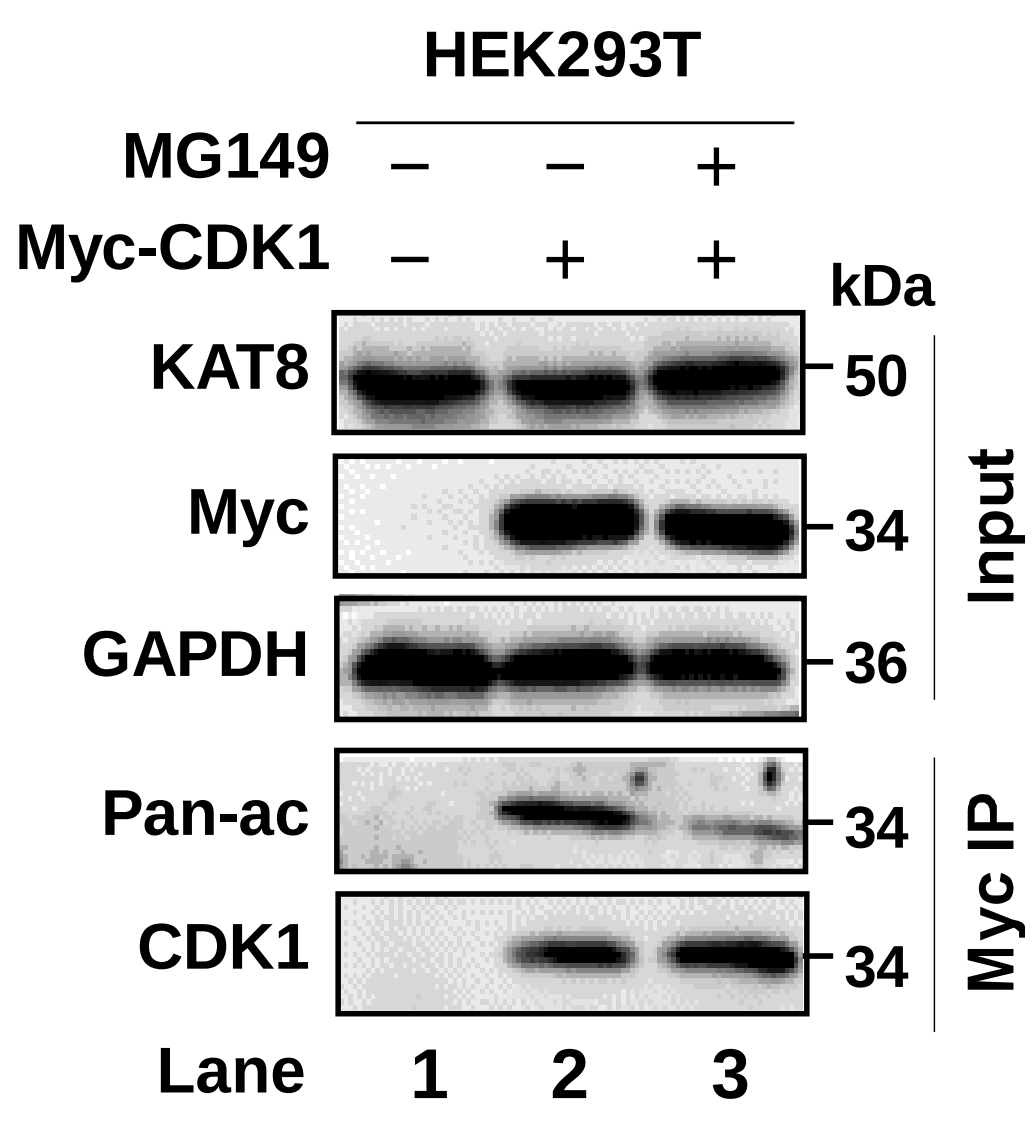
HEK293T
MG149 – – +
Myc-CDK1 – + +
kDa
KAT8
50
Myc
34
GAPDH
36
Input
Pan-ac
34
CDK1
34
Myc IP
Lane 1 2 3

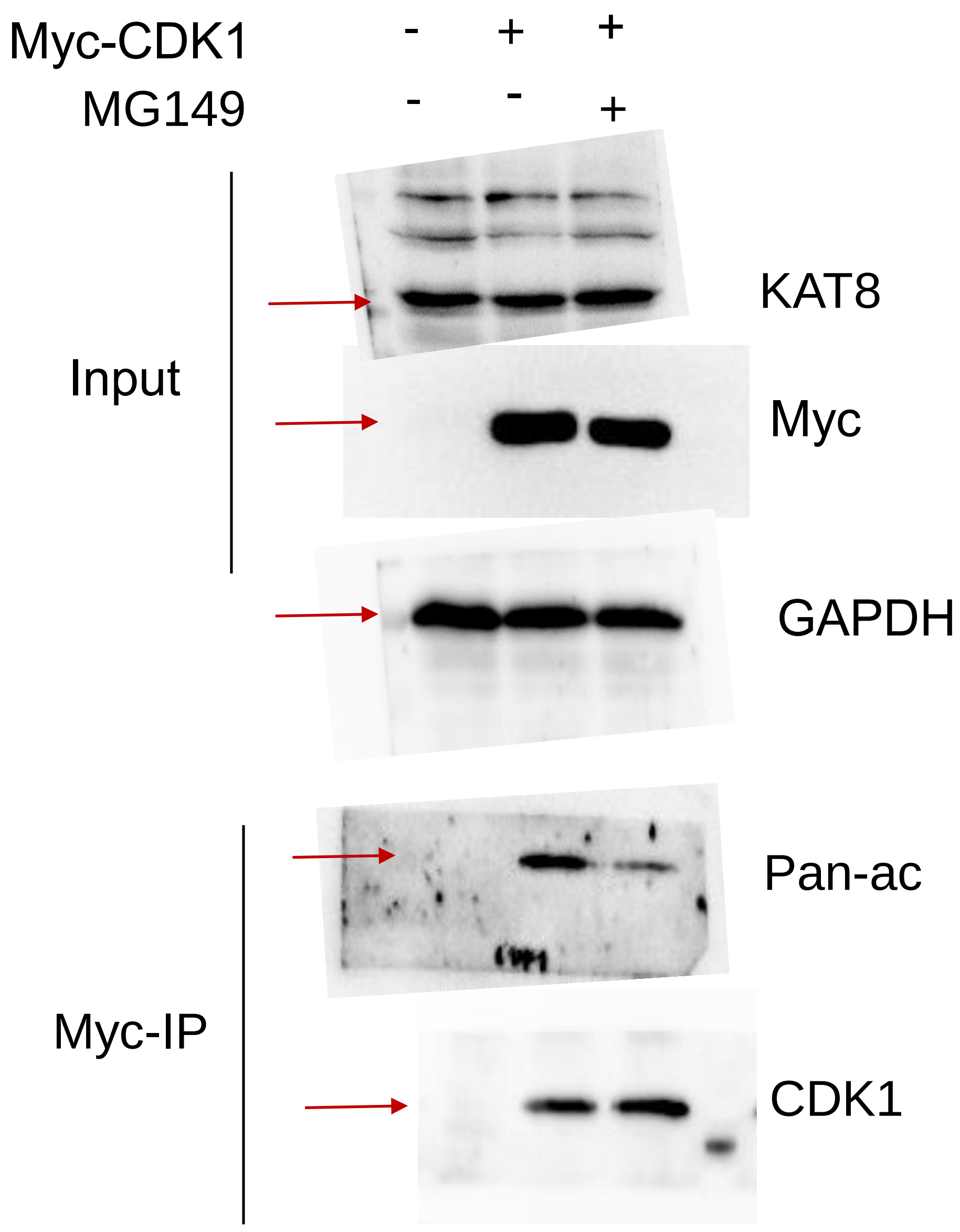
Myc-CDK1 - + +
MG149 - - +
Input
KAT8
Myc
GAPDH
Myc-IP
Pan-ac
CDK1

Figure 5H

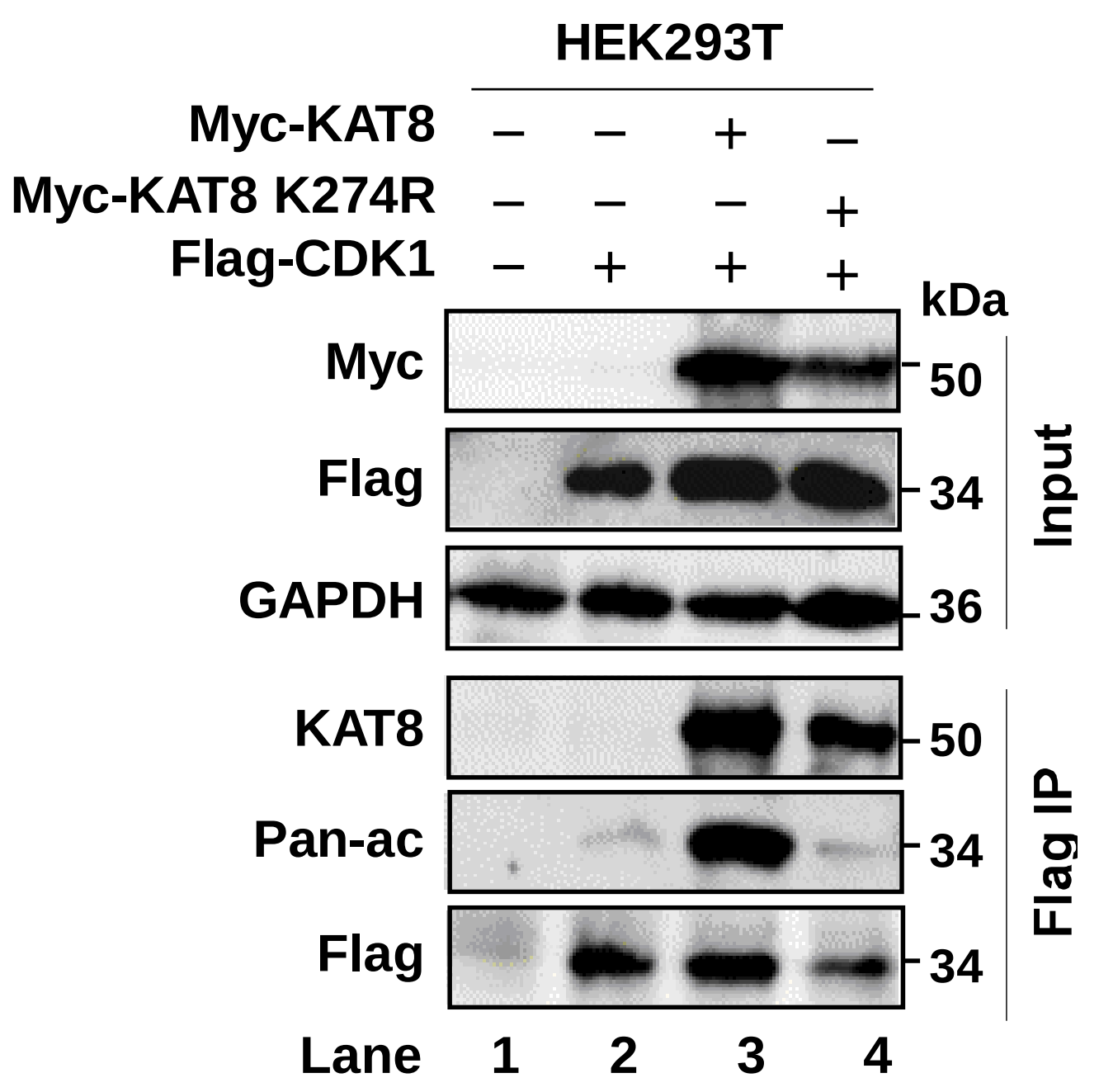
HEK293T
Myc-KAT8 – – + –
Myc-KAT8 K274R – – – +
Flag-CDK1 – + + +
kDa
Myc
50
Flag
34
GAPDH
36
Input
KAT8
50
Pan-ac
34
Flag
34
Flag IP
Lane 1 2 3 4

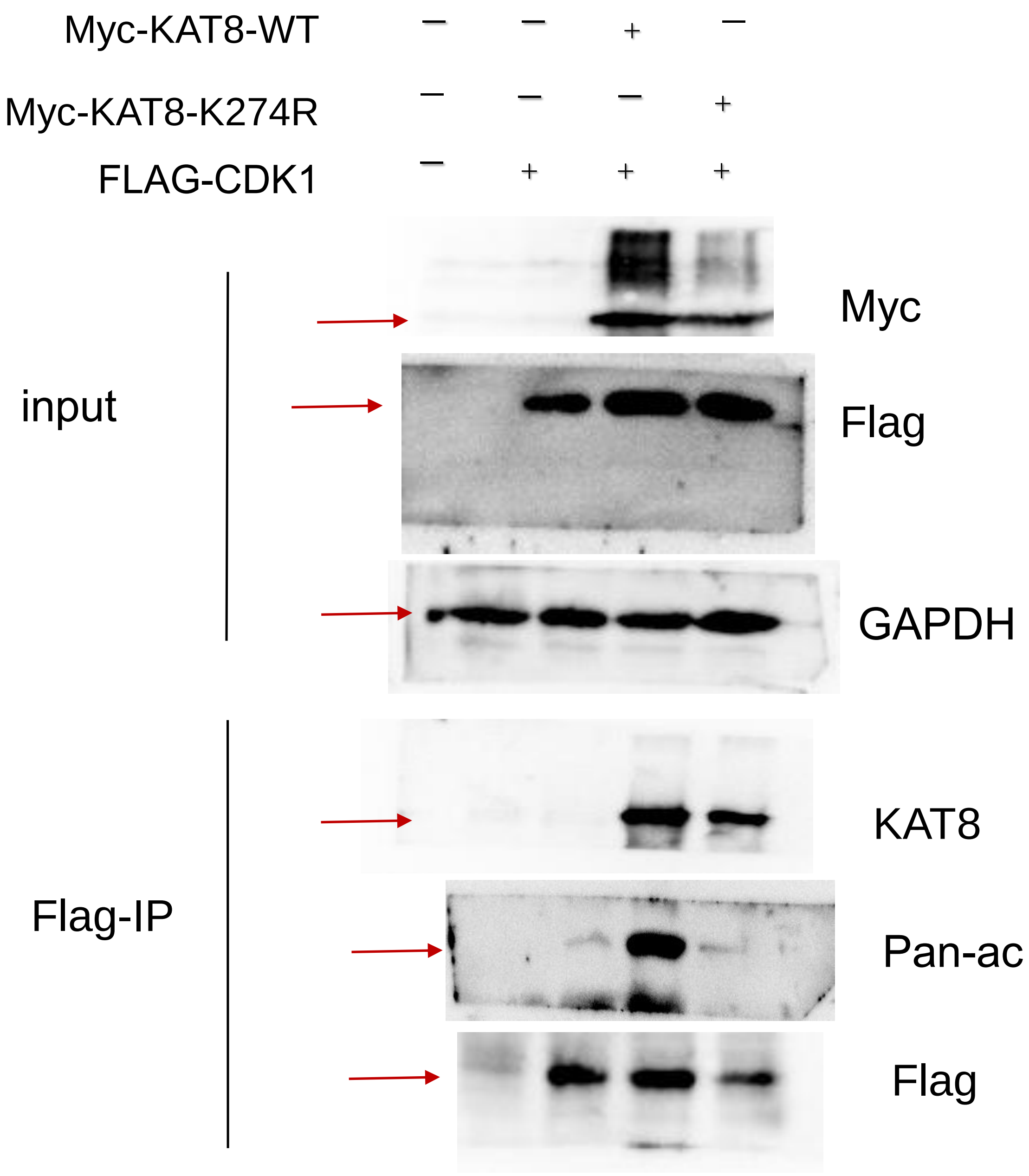
Myc-KAT8-WT – – + –
Myc-KAT8-K274R – – – +
FLAG-CDK1 – + + +
input
Myc
Flag
GAPDH
Flag-IP
KAT8
Pan-ac
Flag

Figure 5I

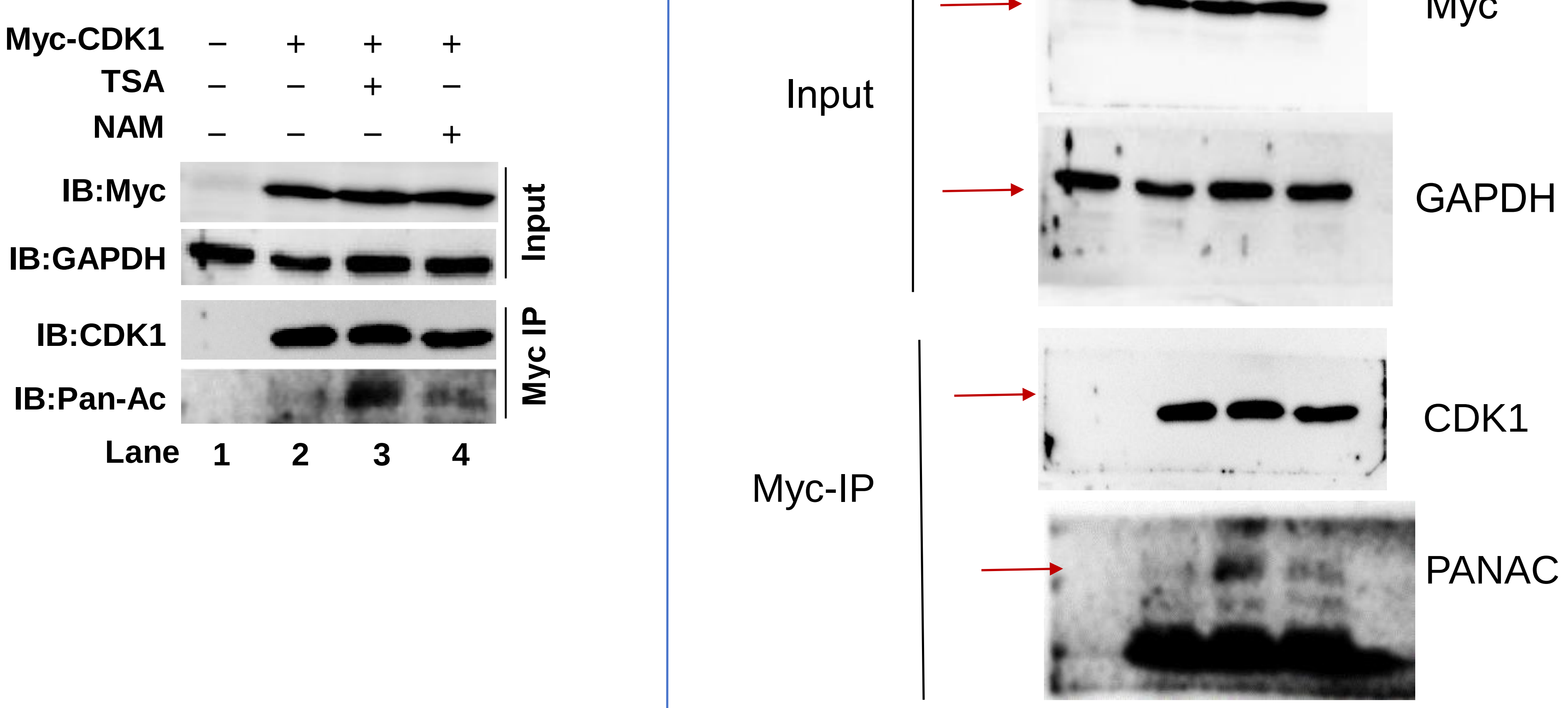

Myc-CDK1 – + + +
TSA – – + –
NAM – – – +
IB:Myc
IB:GAPDH
IB:CDK1
IB:Pan-Ac
Input
Myc IP
Lane 1 2 3 4
TSA
NAM
Myc-CDK1 - + + +
Input
Myc-IP
Myc
GAPDH
CDK1
PANAC

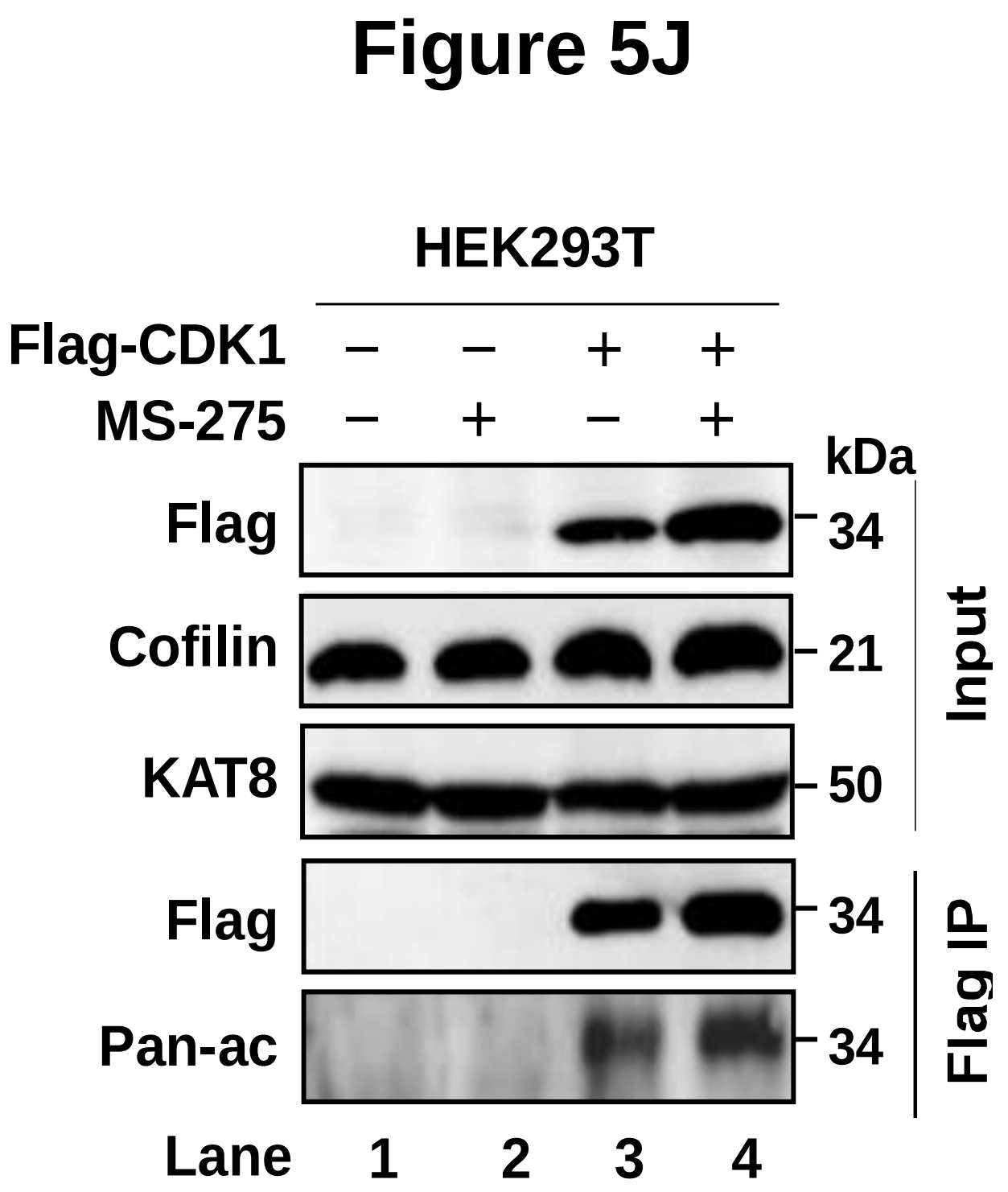
Figure 5J
HEK293T
Flag-CDK1 – – + +
MS-275 – + – +
kDa
Flag
Cofilin
KAT8
Flag
Pan-ac
34
21
50
34
34
Input
Flag IP
Lane 1 2 3 4

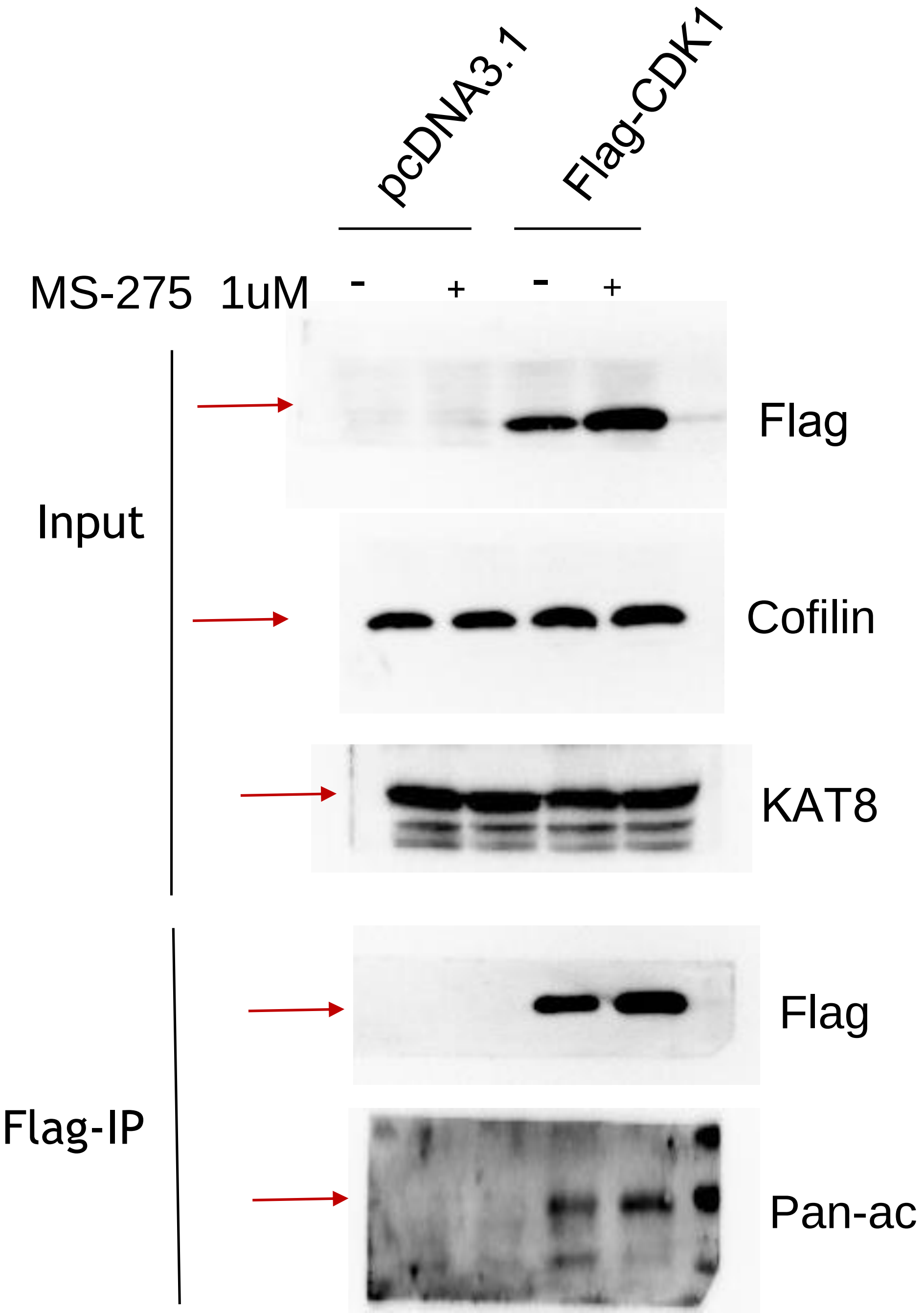
pcDNA3.1
Flag-CDK1
MS-275 1uM - + - +
Input
Flag
Cofilin
KAT8
Flag-IP
Flag
Pan-ac

Figure 5K

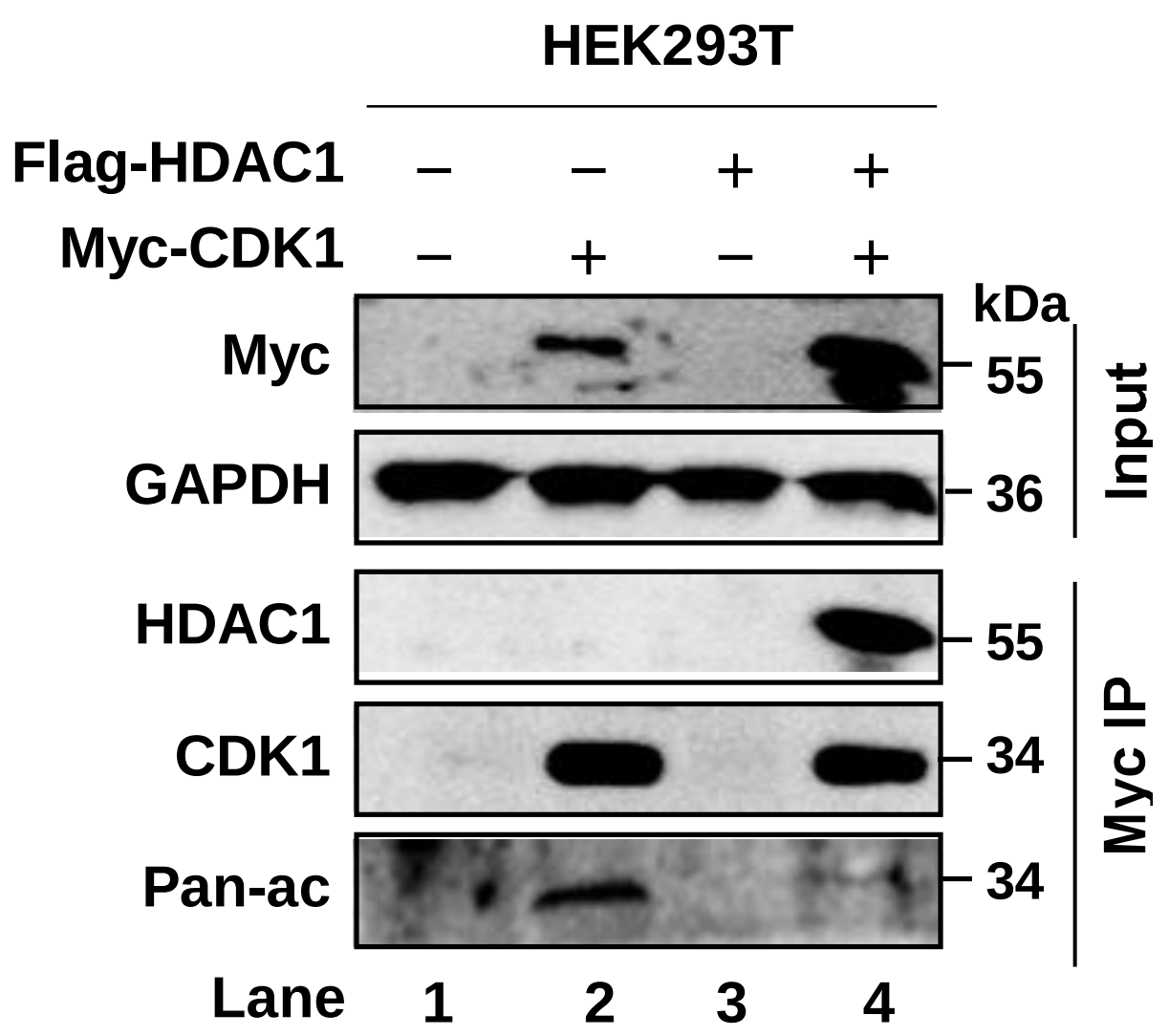
HEK293T
Flag-HDAC1 – – + +
Myc-CDK1 – + – +
kDa
Myc
55
GAPDH
36
Input
HDAC1
55
CDK1
34
Pan-ac
34
Myc IP
Lane 1 2 3 4

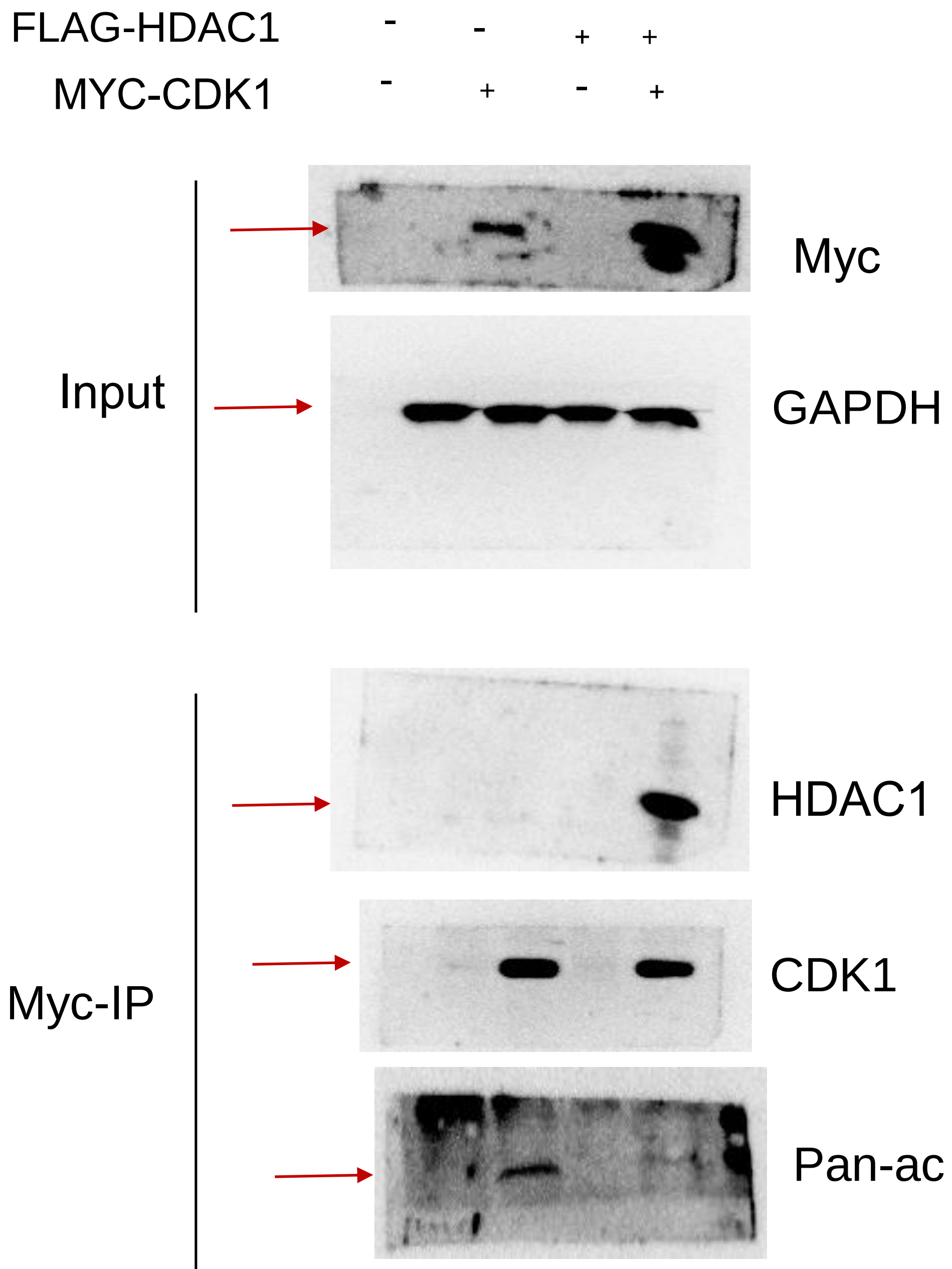
FLAG-HDAC1 - - + +
MYC-CDK1 - + - +
Input
Myc
GAPDH
Myc-IP
HDAC1
CDK1
Pan-ac

Figure 5M

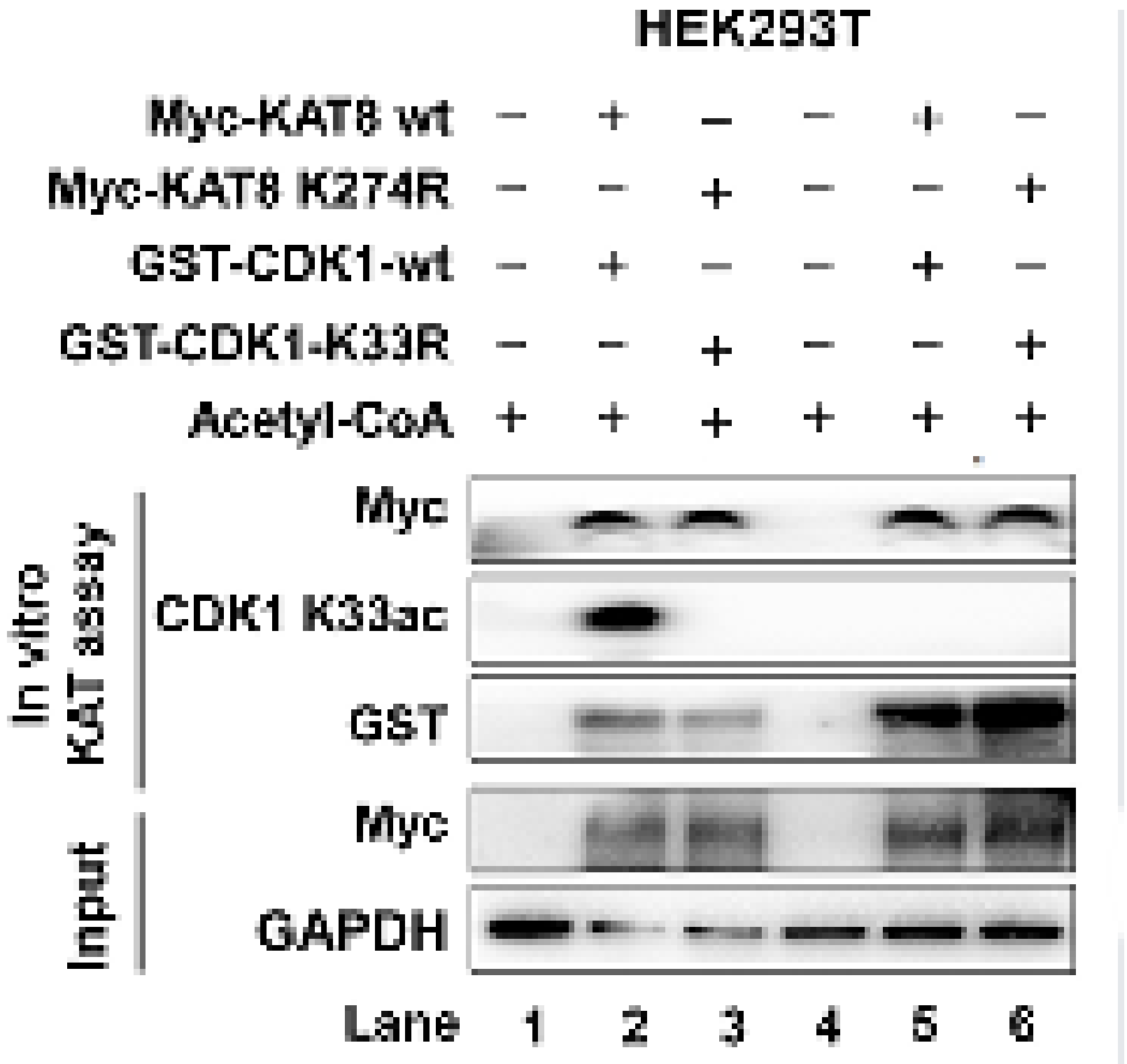
HEK293T
Myc-KAT8 wt
Myc-KAT8 K274R
GST-CDK1-wt
GST-CDK1-K33R
Acetyl-CoA
In vitro KAT assay
Input
Myc
CDK1 K33ac
GST
Myc
GAPDH
Lane 1 2 3 4 5 6

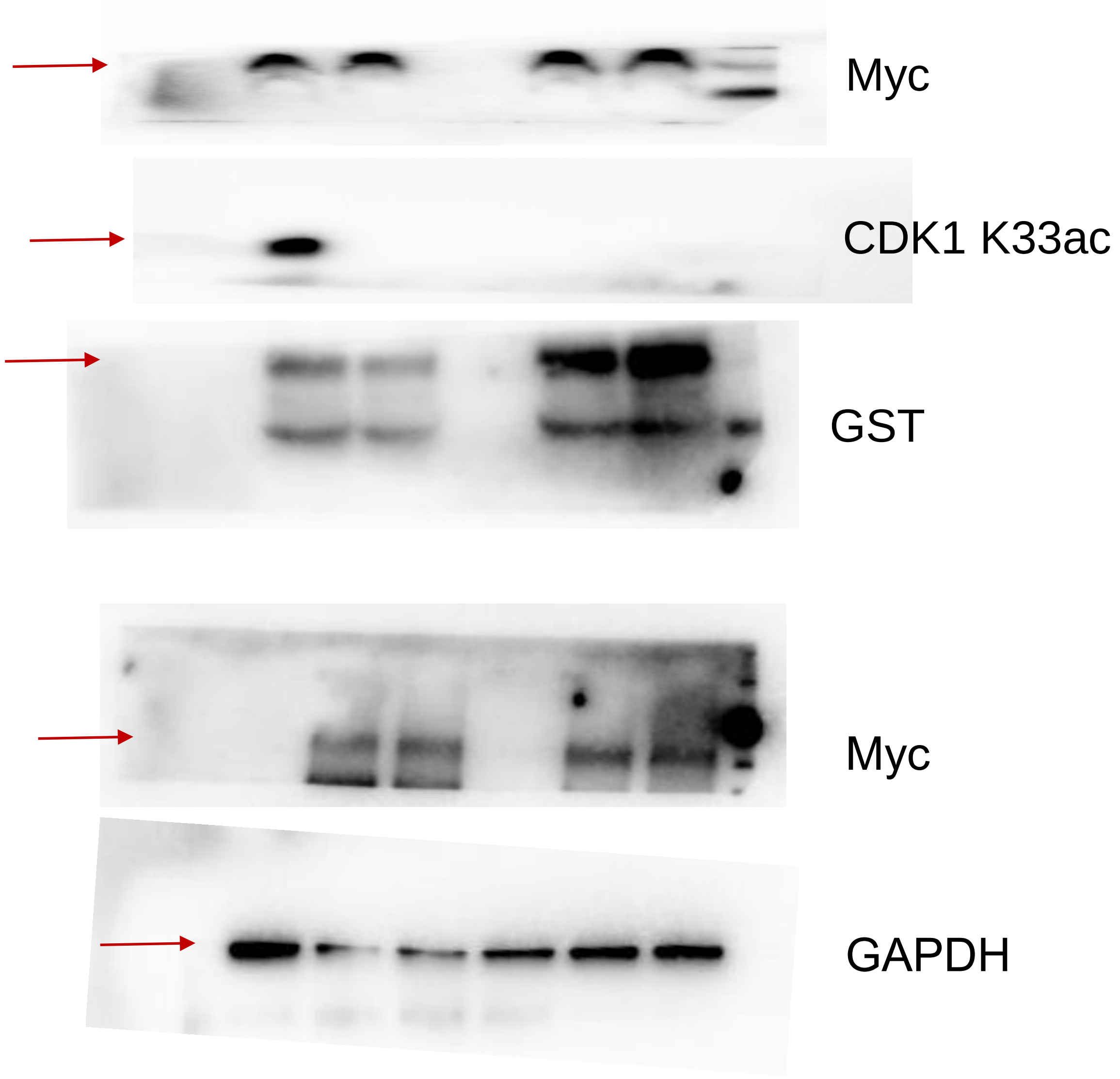
Myc
CDK1 K33ac
GST
Myc
GAPDH

Figure 5N

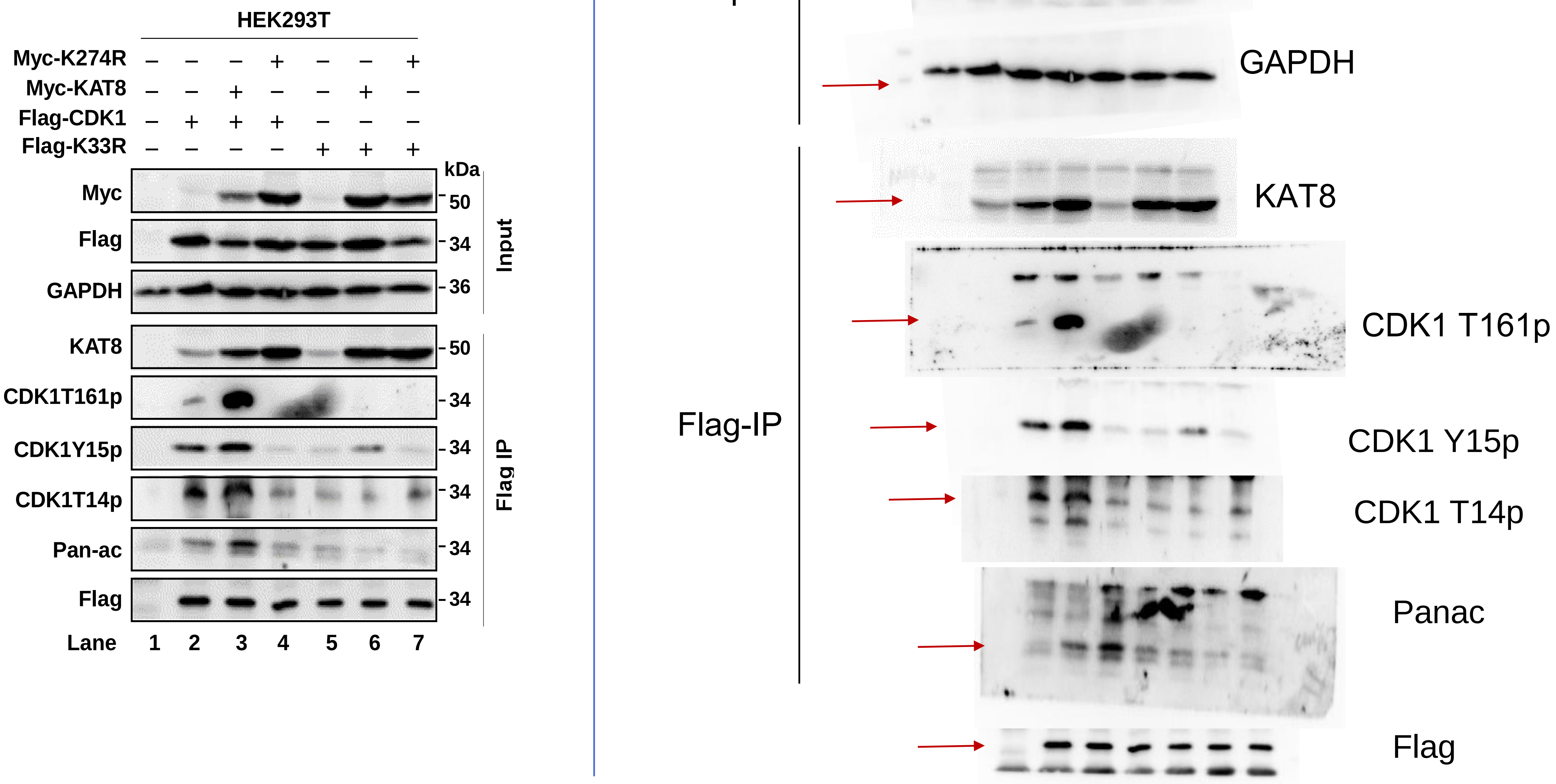
HEK293T
Myc-K274R – – – + – – +
Myc-KAT8 – – + – – + –
Flag-CDK1 – + + + – – –
Flag-K33R – – – – + + +
kDa
Myc
Flag
GAPDH
KAT8
CDK1T161p
CDK1Y15p
CDK1T14p
Pan-ac
Flag
50
34
36
50
34
34
34
34
34
Input
Flag IP
Lane 1 2 3 4 5 6 7
Input
Flag-IP
Myc
Flag
GAPDH
KAT8
CDK1 T161p
CDK1 Y15p
CDK1 T14p
Panac
Flag

Figure 5O

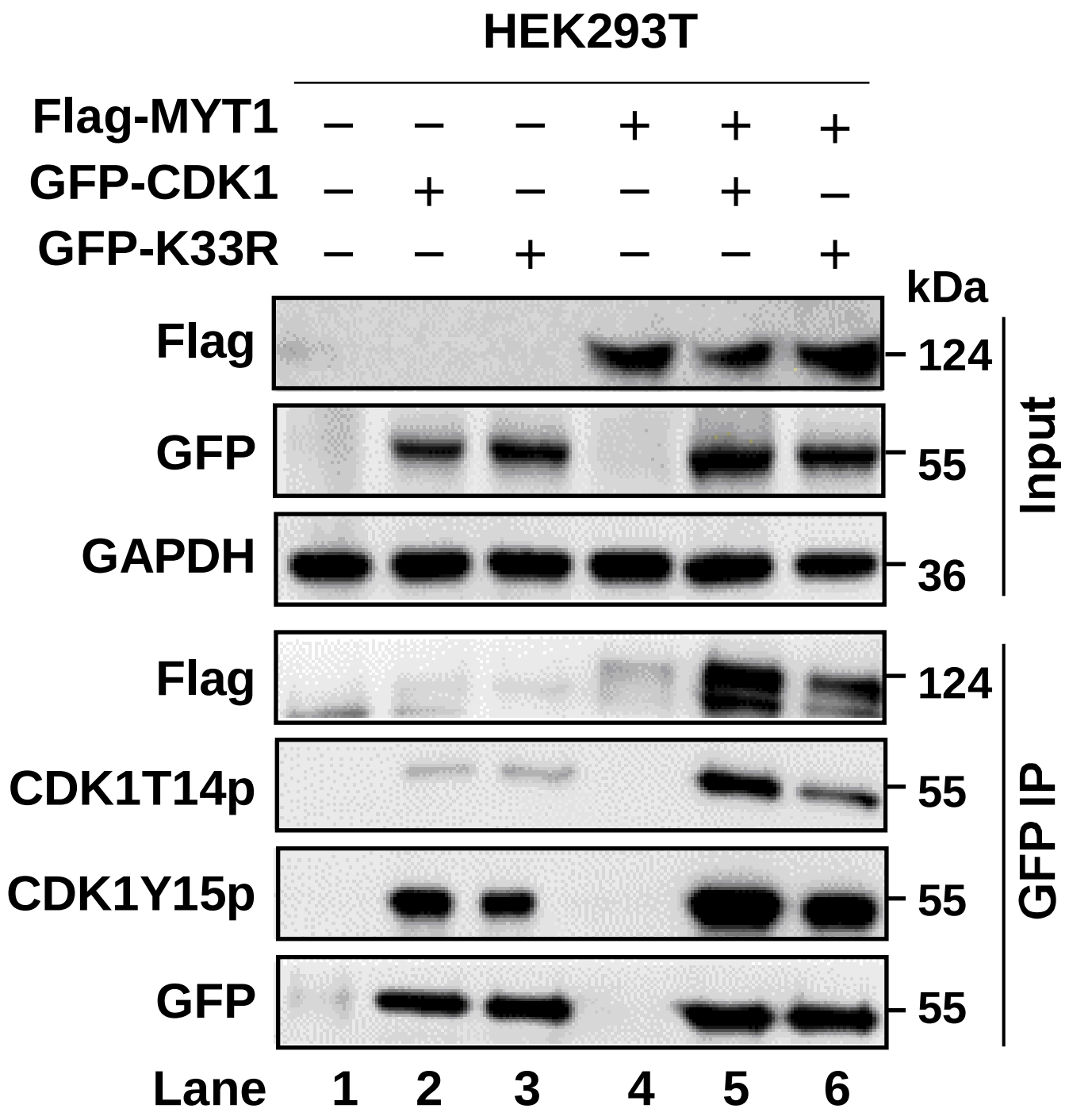
HEK293T
Flag-MYT1 – – – + + +
GFP-CDK1 – + – – + –
GFP-K33R – – + – – +
kDa
Flag
GFP
GAPDH
Flag
CDK1T14p
CDK1Y15p
GFP
124
55
36
124
55
55
55
Input
GFP IP
Lane 1 2 3 4 5 6

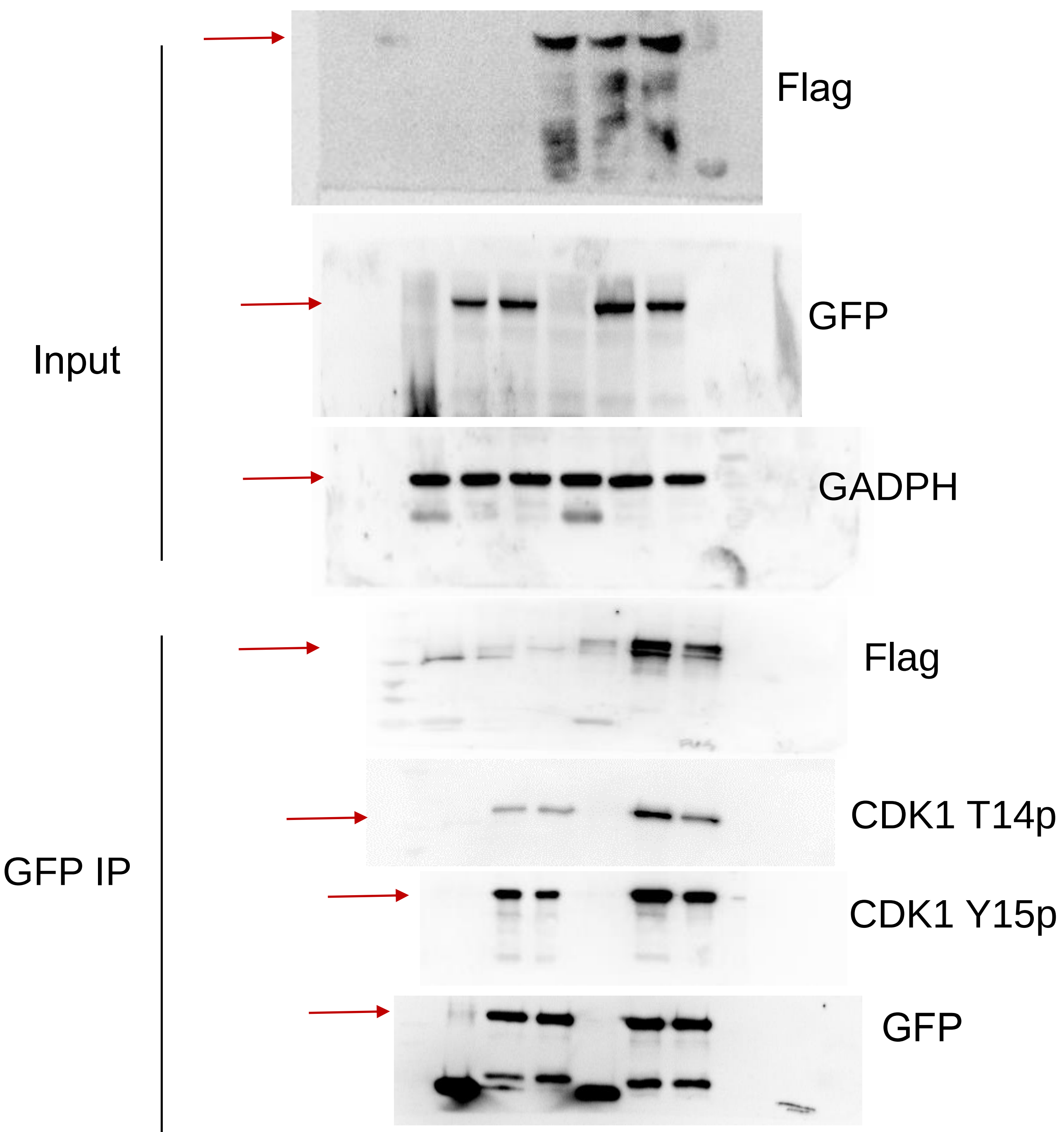
Input
GFP IP
Flag
GFP
GADPH
Flag
CDK1 T14p
CDK1 Y15p
GFP

Figure 5P

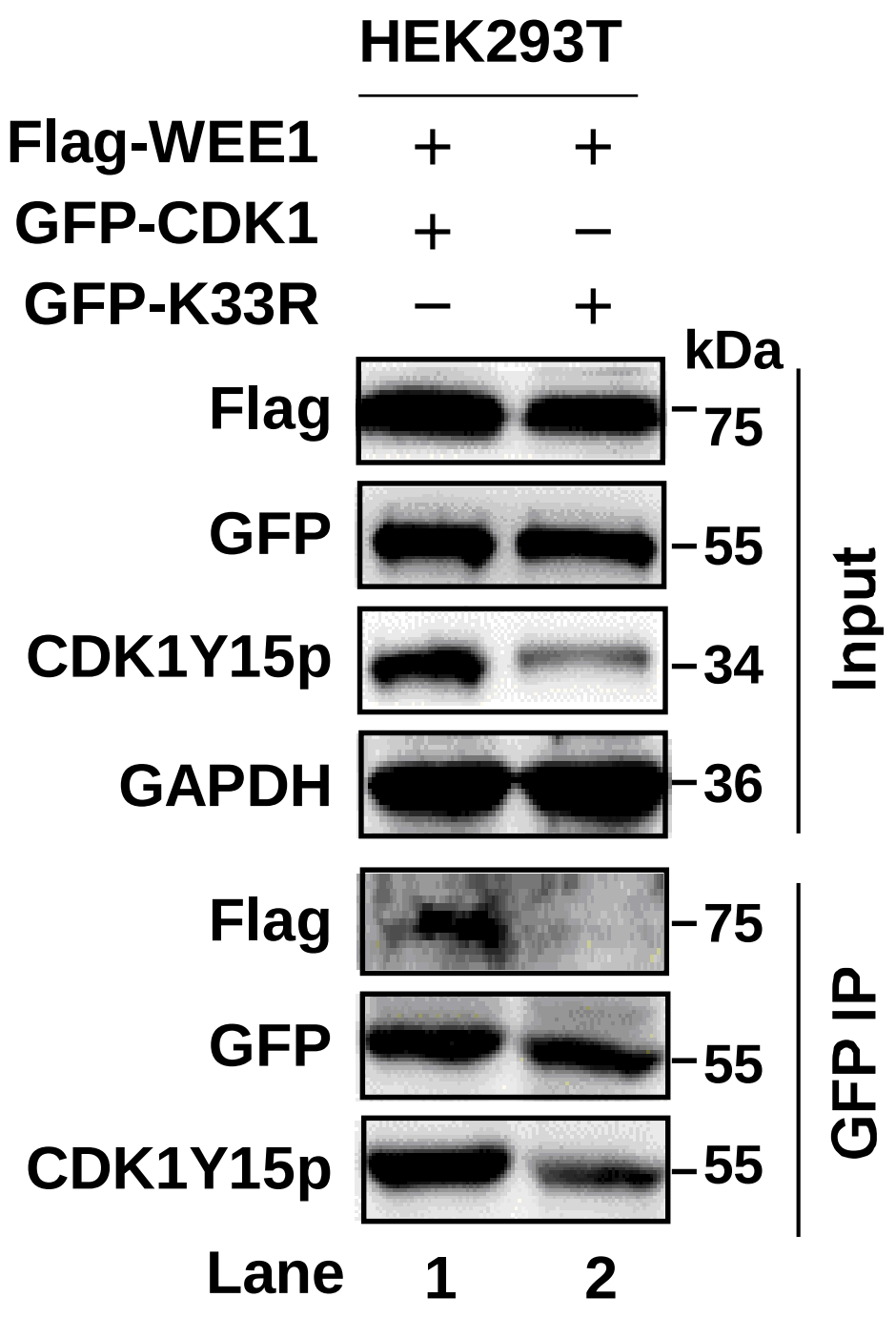
HEK293T
Flag-WEE1 + +
GFP-CDK1 + –
GFP-K33R – +
kDa
Flag 75
GFP 55
CDK1Y15p 34
GAPDH 36
Input
Flag 75
GFP 55
CDK1Y15p 55
GFP IP
Lane 1 2

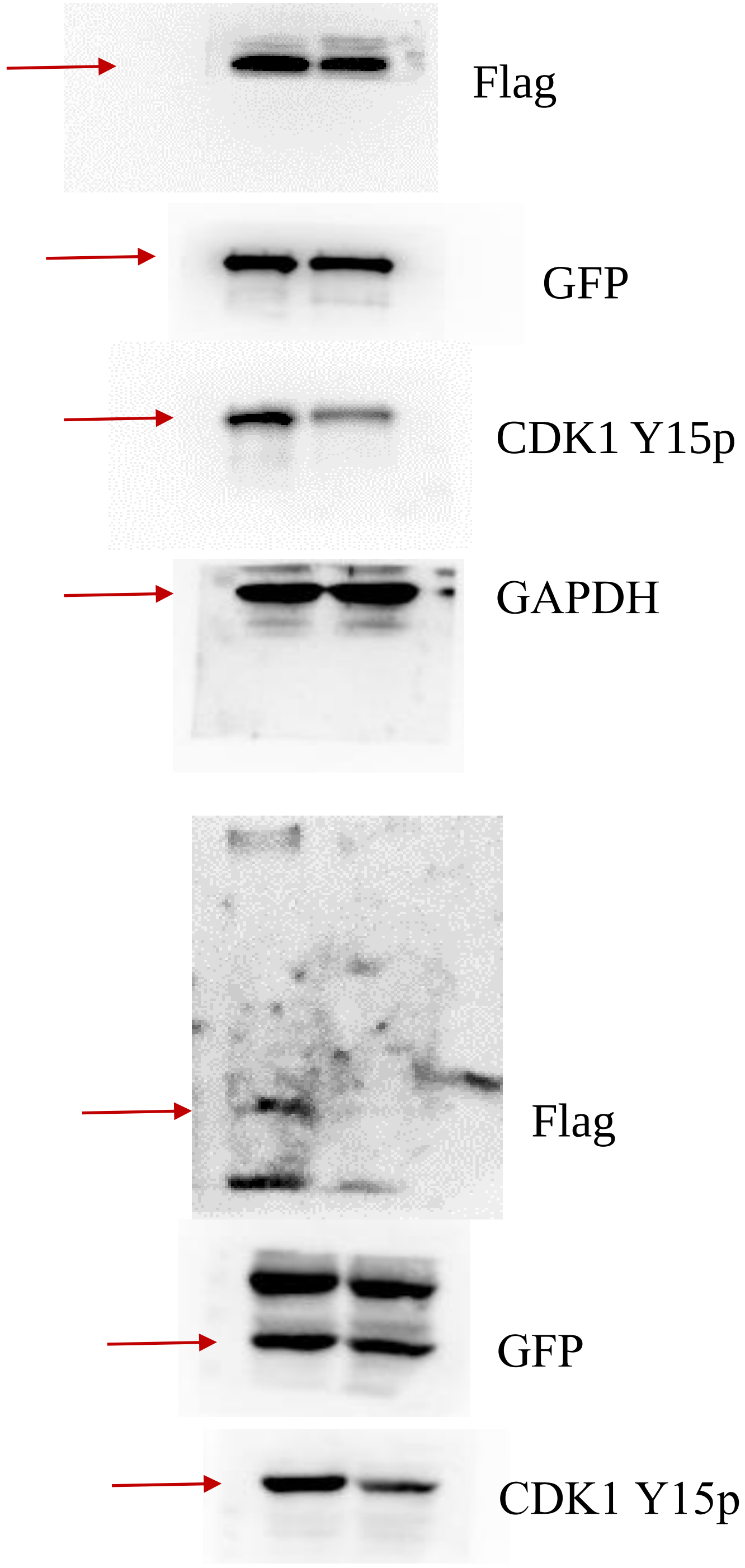
Flag
GFP
CDK1 Y15p
GAPDH
Flag
GFP
CDK1 Y15p

Figure 5Q

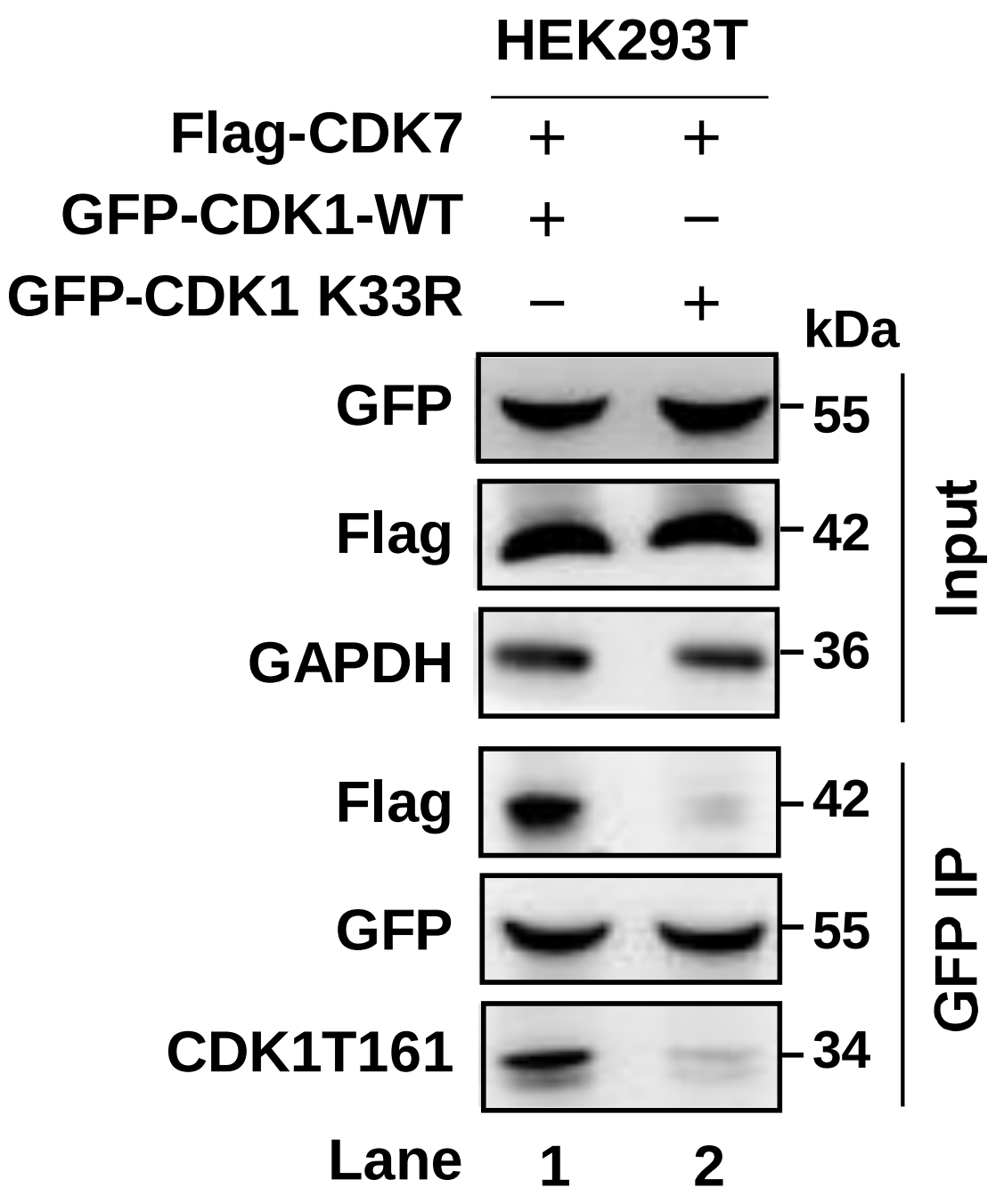
HEK293T
Flag-CDK7 + +
GFP-CDK1-WT + –
GFP-CDK1 K33R – +
kDa
GFP
Flag
GAPDH
Flag
GFP
CDK1T161
55
42
36
42
55
34
Input
GFP IP
Lane 1 2

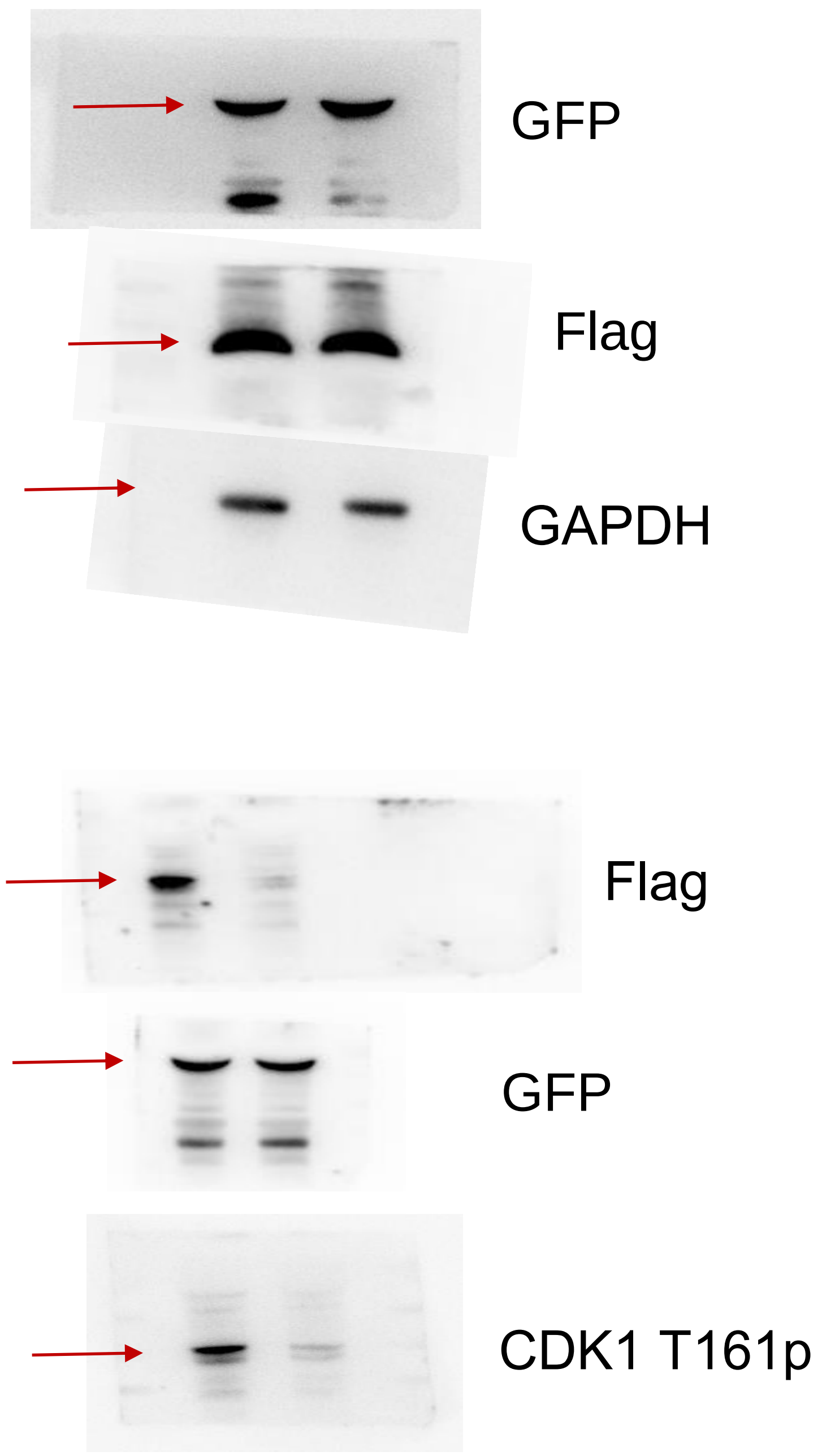
GFP
Flag
GAPDH
Flag
GFP
CDK1 T161p

Figure 6A

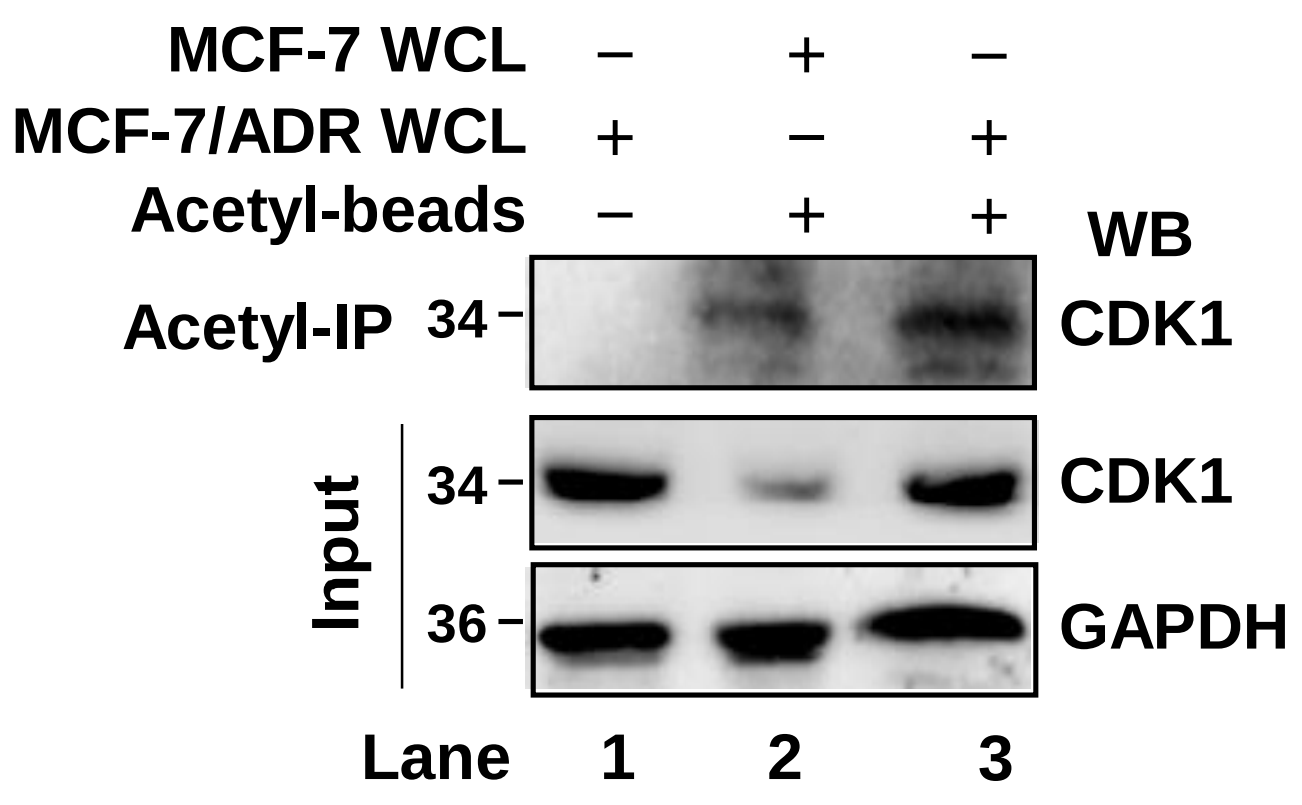

MCF-7 WCL − + −
MCF-7/ADR WCL + − +
Acetyl-beads − + +
WB
Acetyl-IP
34
CDK1
Input
34
CDK1
36
GAPDH
Lane 1 2 3

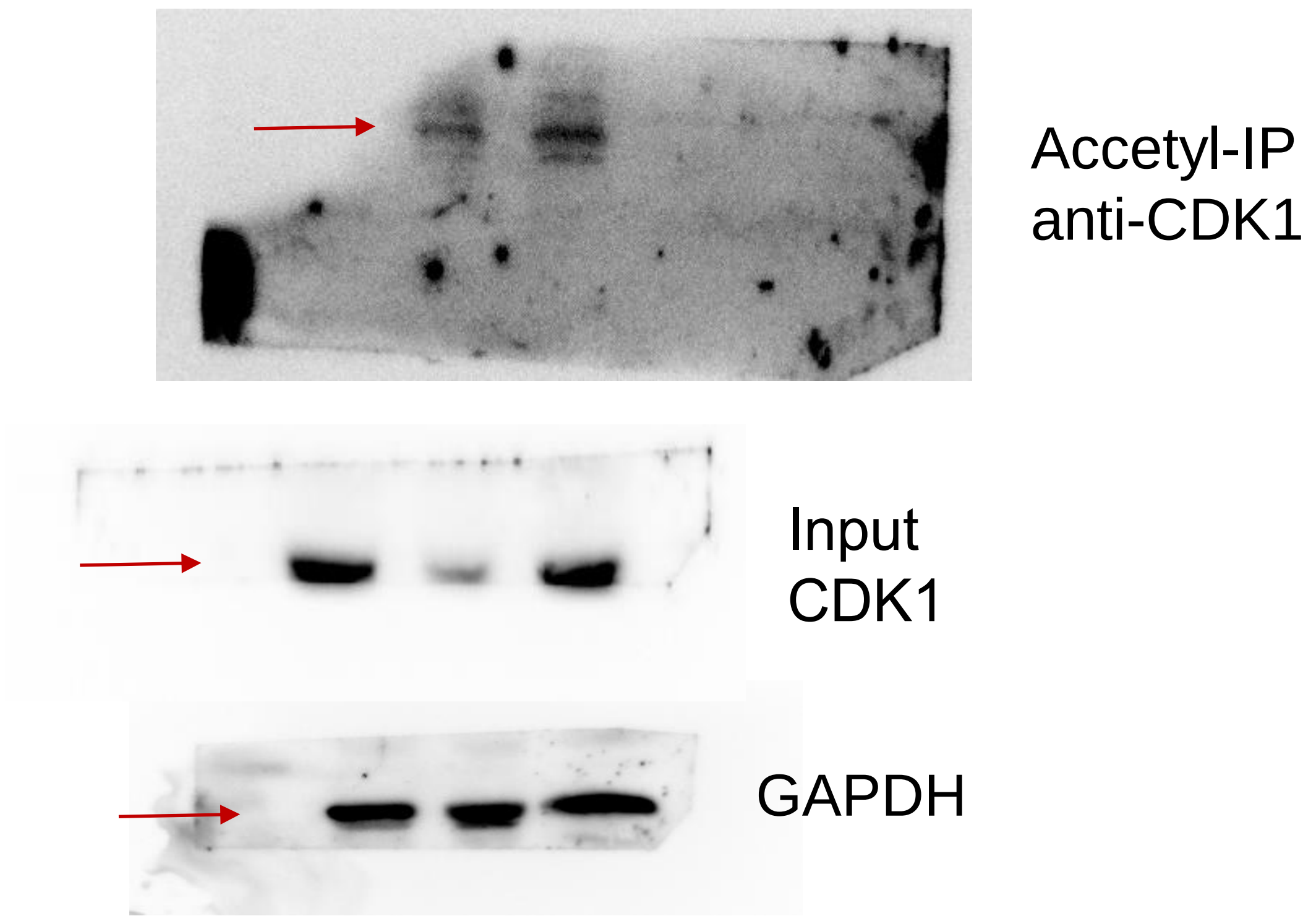

Accetyl-IP
anti-CDK1
Input
CDK1
GAPDH

Figure 6B

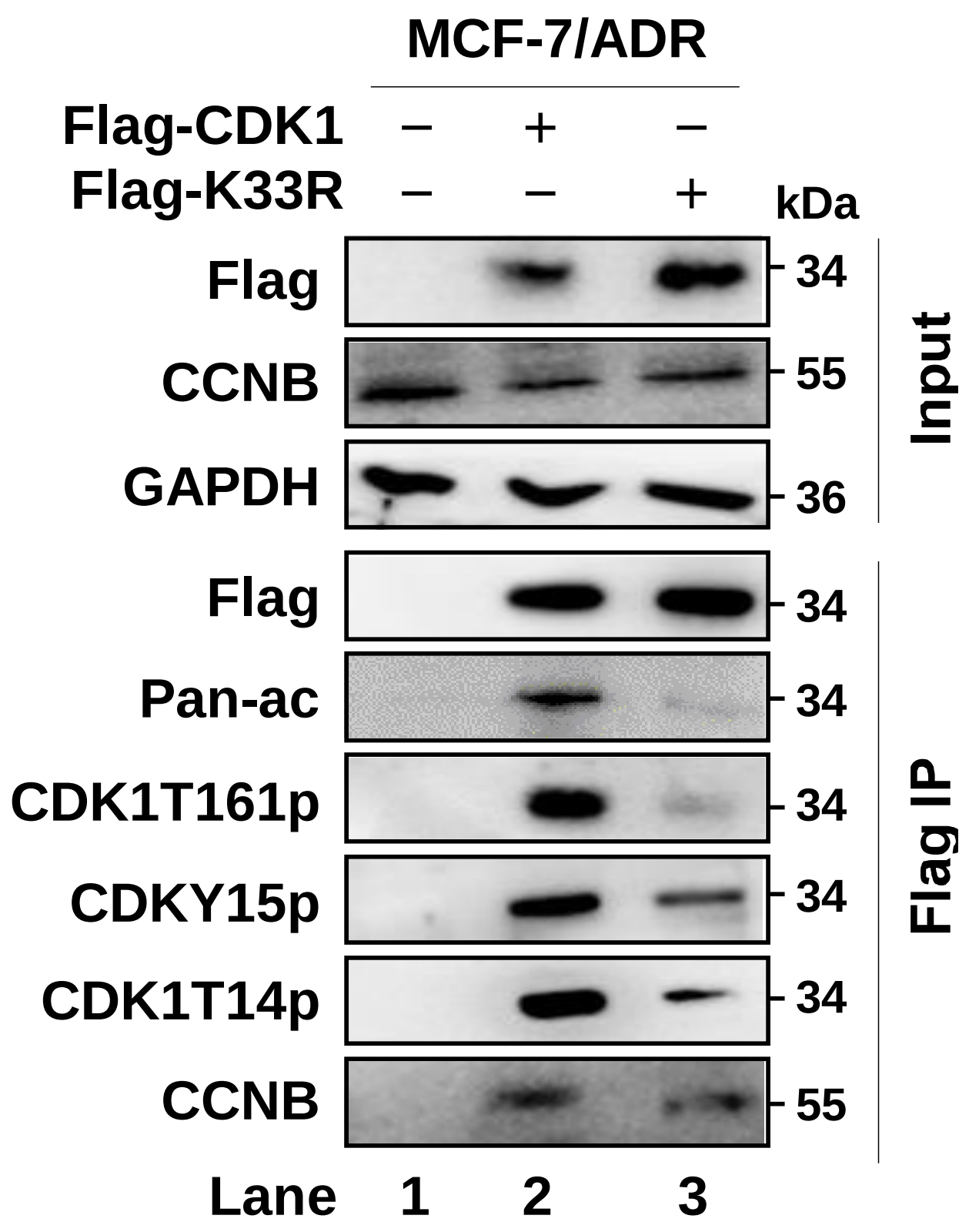

MCF-7/ADR
Flag-CDK1 – + –
Flag-K33R – – +
kDa
Flag 34
CCNB 55
GAPDH 36
Input
Flag 34
Pan-ac 34
CDK1T161p 34
CDKY15p 34
CDK1T14p 34
CCNB 55
Flag IP
Lane 1 2 3

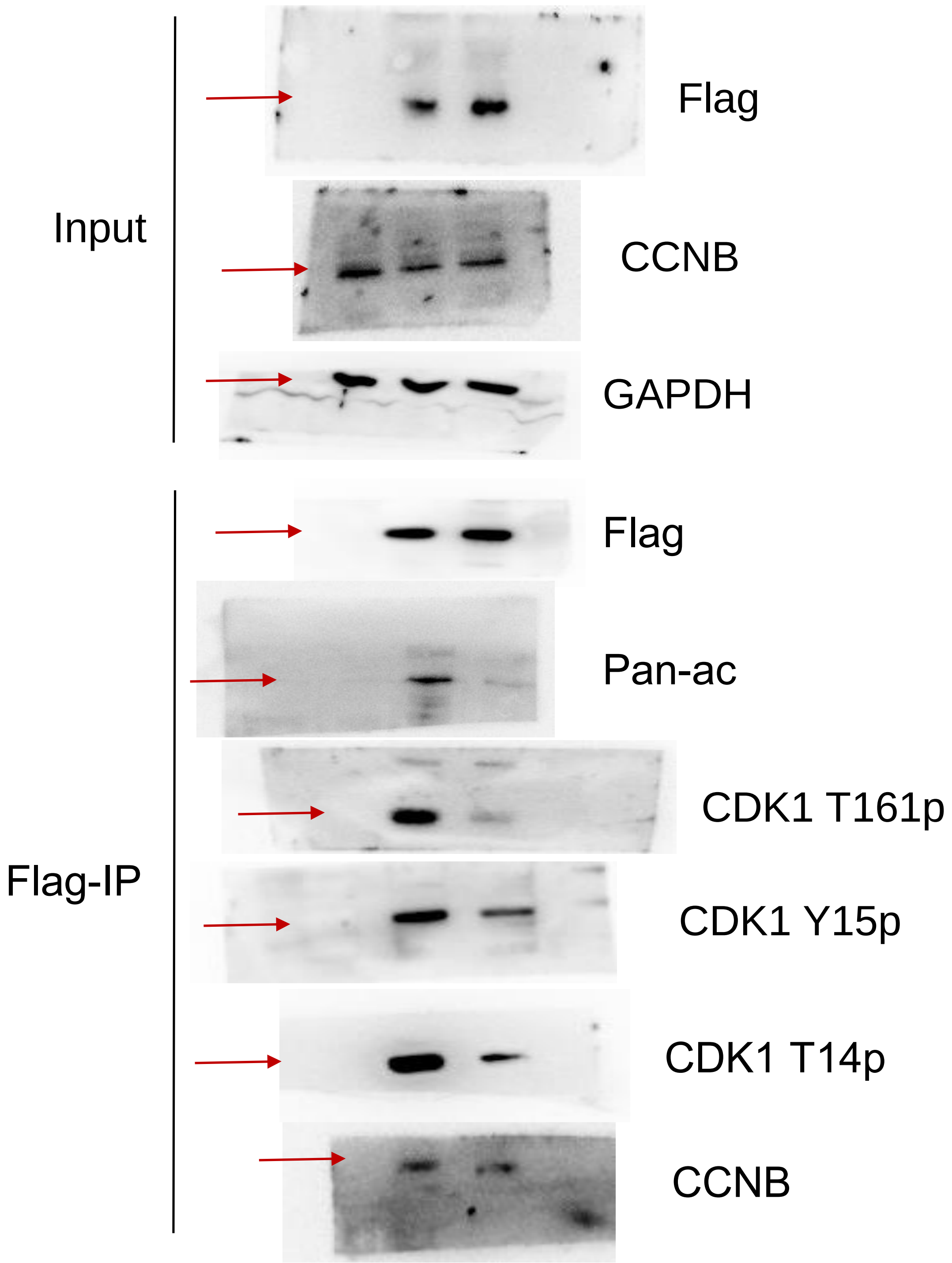

Input
Flag
CCNB
GAPDH
Flag-IP
Flag
Pan-ac
CDK1 T161p
CDK1 Y15p
CDK1 T14p
CCNB

Figure 6G

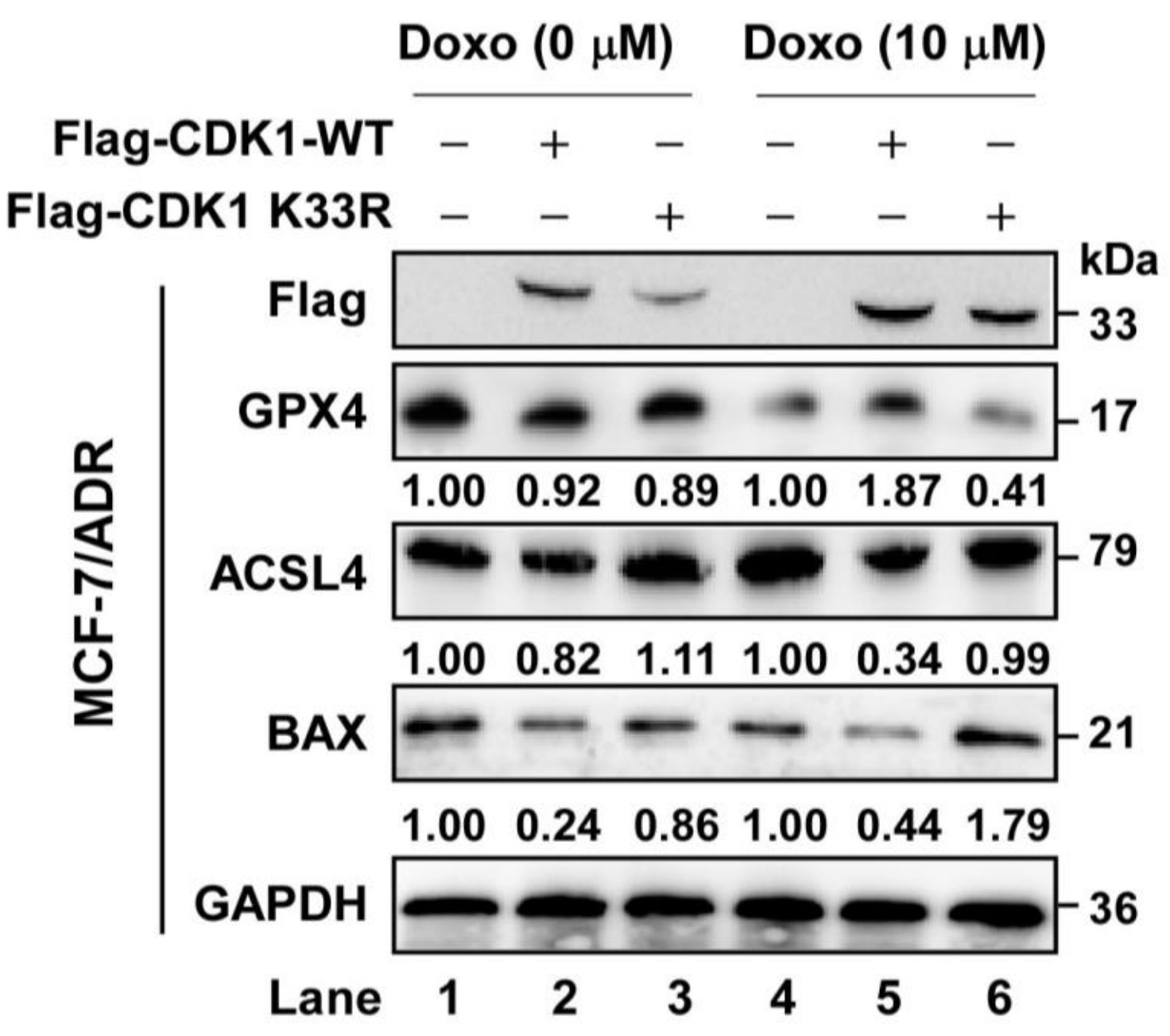

Doxo (0 μM)
Doxo (10 μM)
Flag-CDK1-WT
Flag-CDK1 K33R
MCF-7/ADR
Flag
GPX4
ACSL4
BAX
GAPDH
kDa
33
17
79
21
36
1.00 0.92 0.89 1.00 1.87 0.41
1.00 0.82 1.11 1.00 0.34 0.99
1.00 0.24 0.86 1.00 0.44 1.79
Lane 1 2 3 4 5 6

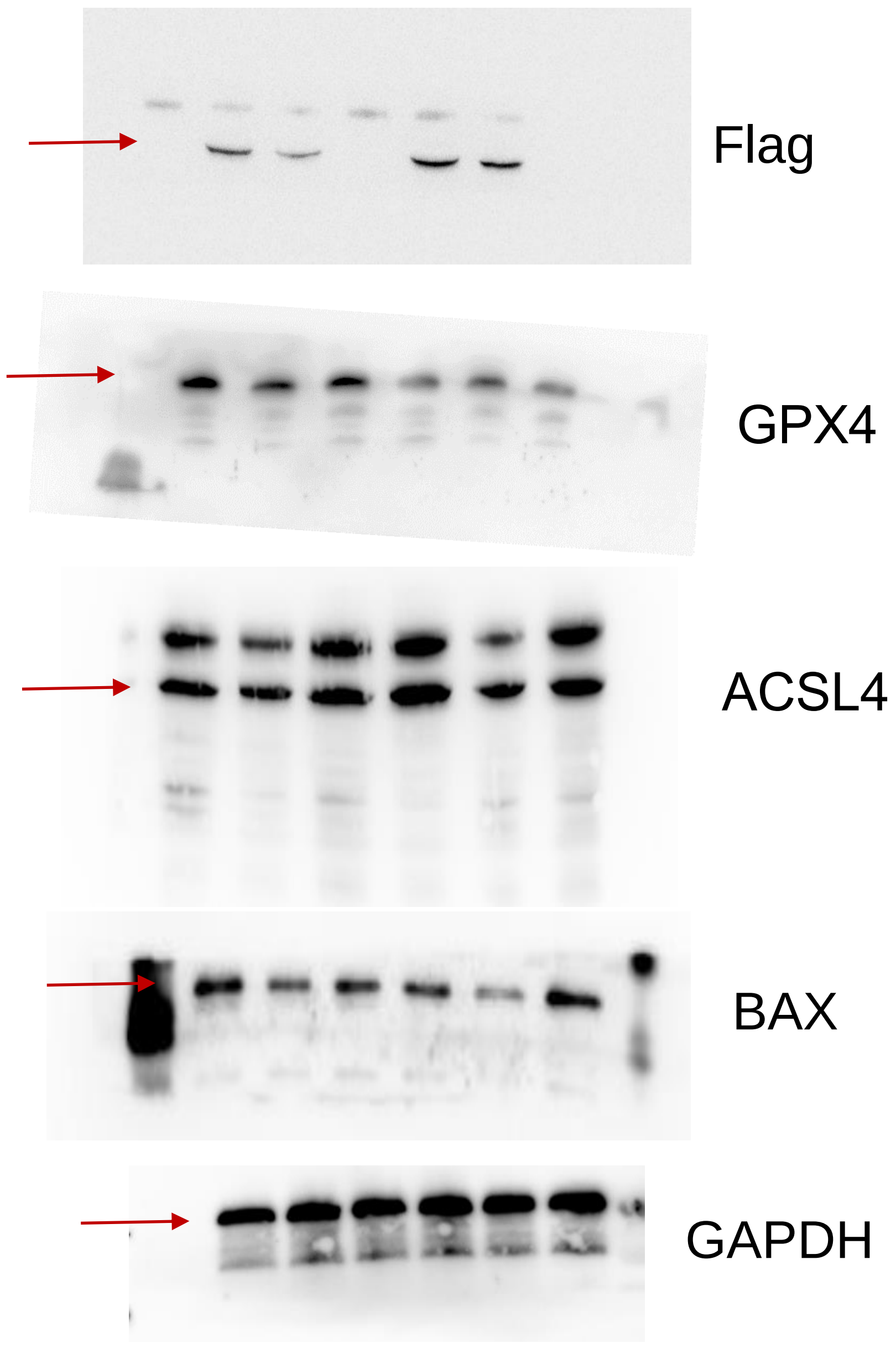

Flag
GPX4
ACSL4
BAX
GAPDH

Figure 7D

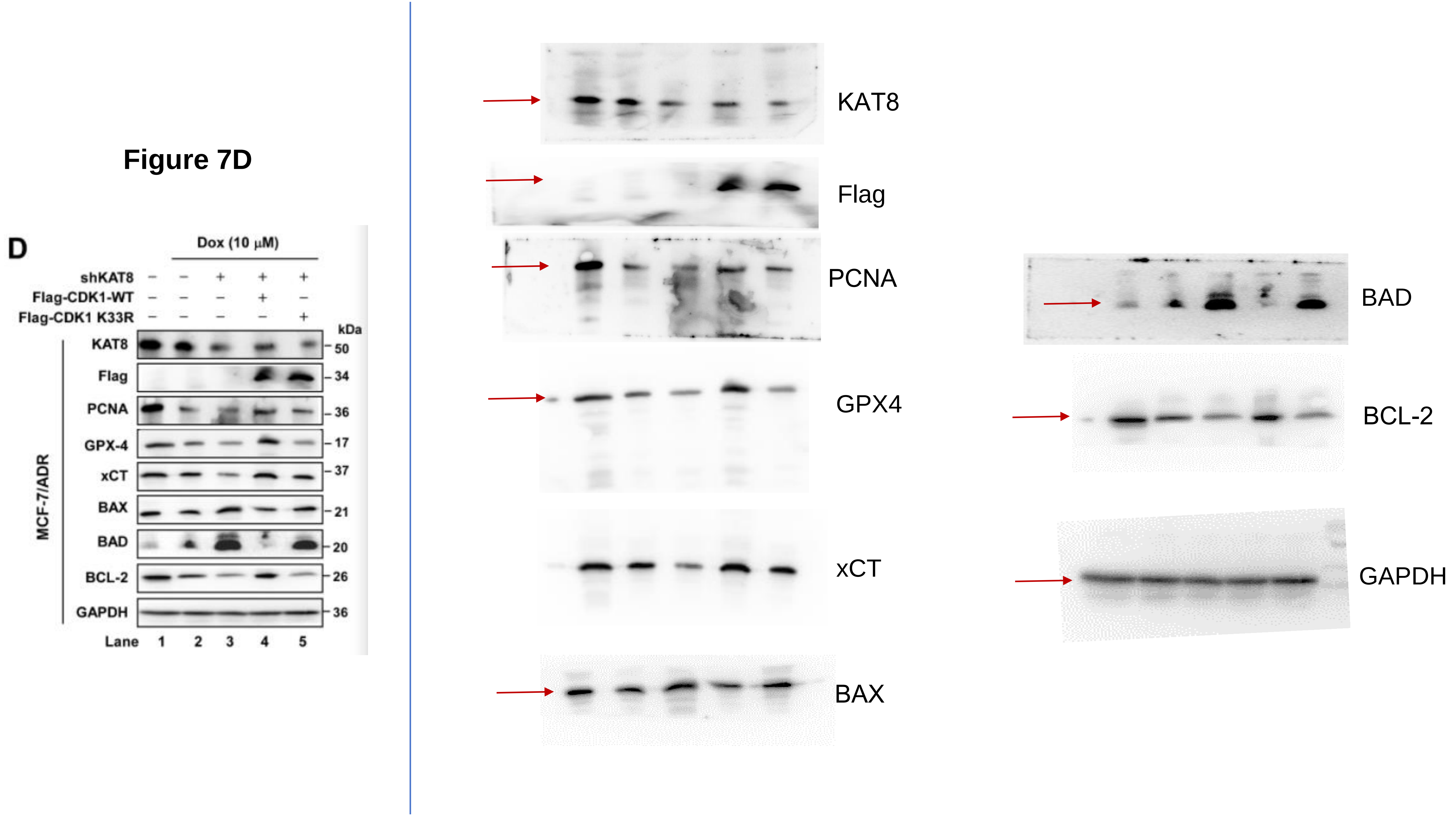
D
Dox (10 μM)
shKAT8 − − + + +
Flag-CDK1-WT − − − + −
Flag-CDK1 K33R − − − − +
kDa
MCF-7/ADR
KAT8 50
Flag 34
PCNA 36
GPX-4 17
xCT 37
BAX 21
BAD 20
BCL-2 26
GAPDH 36
Lane 1 2 3 4 5
KAT8
Flag
PCNA
GPX4
xCT
BAX
BAD
BCL-2
GAPDH

Figure 8K

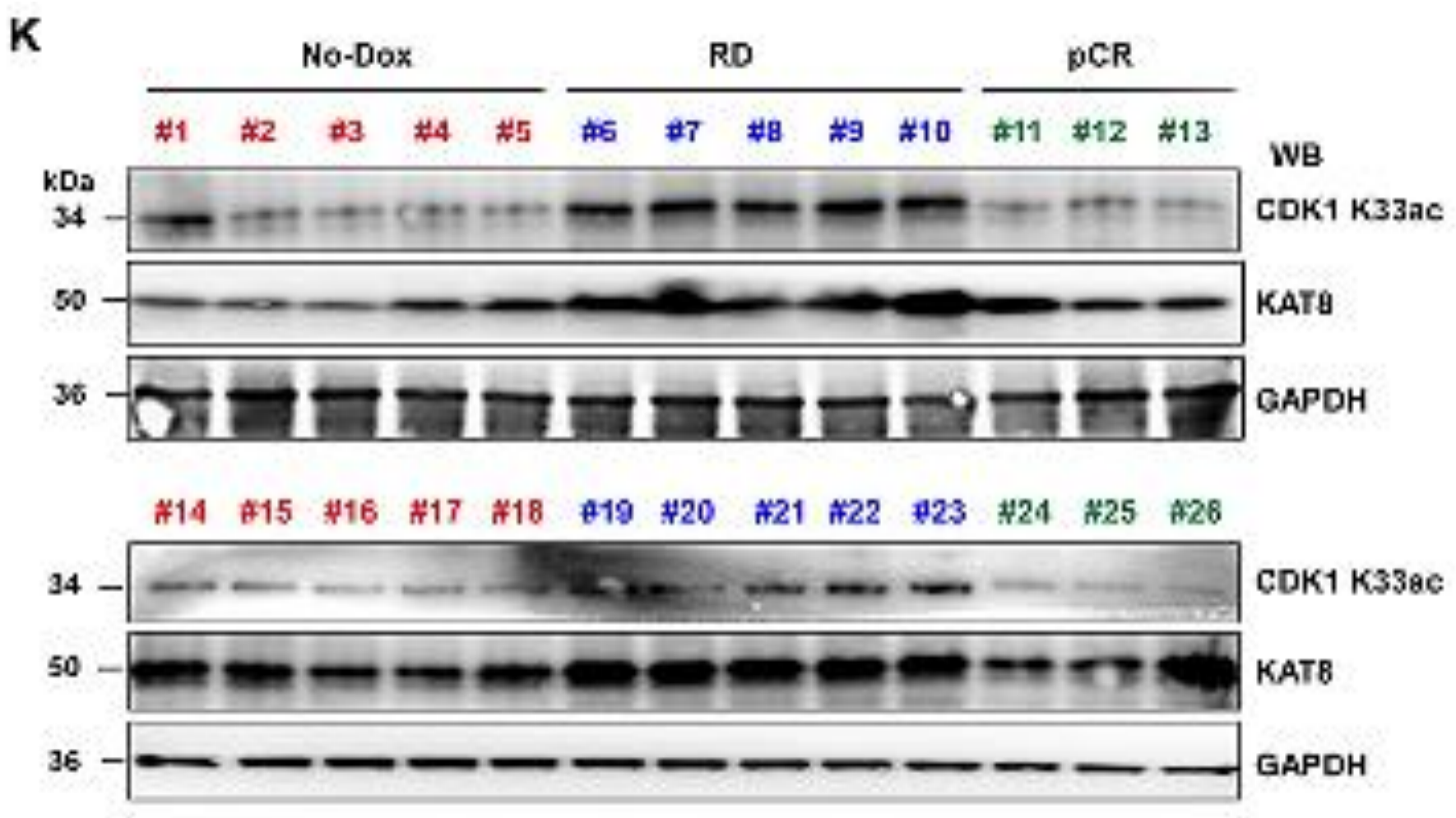
K
No-Dox
RD
pCR
#1 #2 #3 #4 #5 #6 #7 #8 #9 #10 #11 #12 #13
WB
kDa
34
CDK1 K33ac
50
KAT8
36
GAPDH
#14 #15 #16 #17 #18 #19 #20 #21 #22 #23 #24 #25 #26
34
CDK1 K33ac
50
KAT8
36
GAPDH

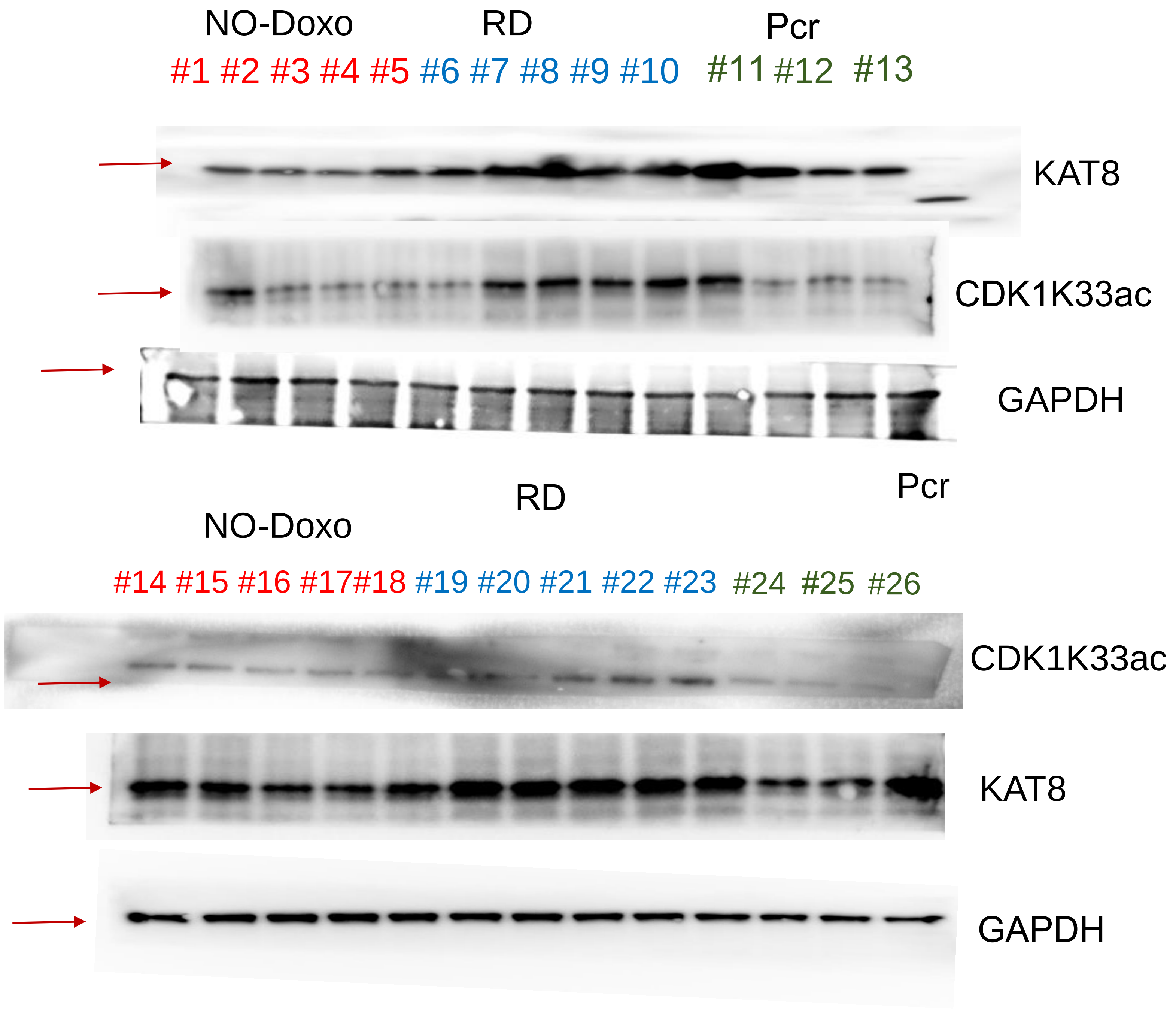
NO-Doxo
RD
Pcr
#1 #2 #3 #4 #5 #6 #7 #8 #9 #10 #11 #12 #13
KAT8
CDK1K33ac
GAPDH
NO-Doxo
RD
Pcr
#14 #15 #16 #17#18 #19 #20 #21 #22 #23 #24 #25 #26
CDK1K33ac
KAT8
GAPDH

Figure S2A

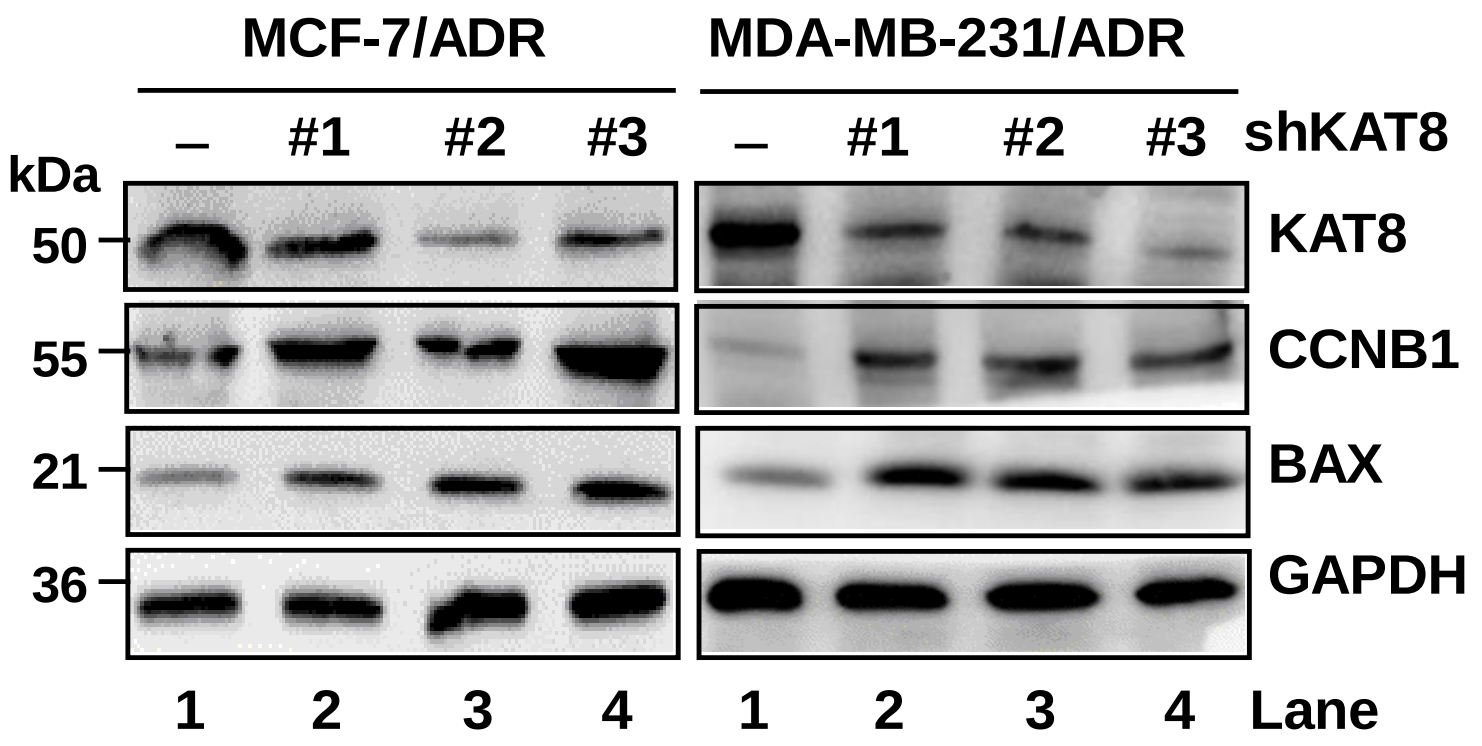
MCF-7/ADR
MDA-MB-231/ADR
kDa
– #1 #2 #3 – #1 #2 #3 shKAT8
50
55
21
36
KAT8
CCNB1
BAX
GAPDH
1 2 3 4 1 2 3 4 Lane

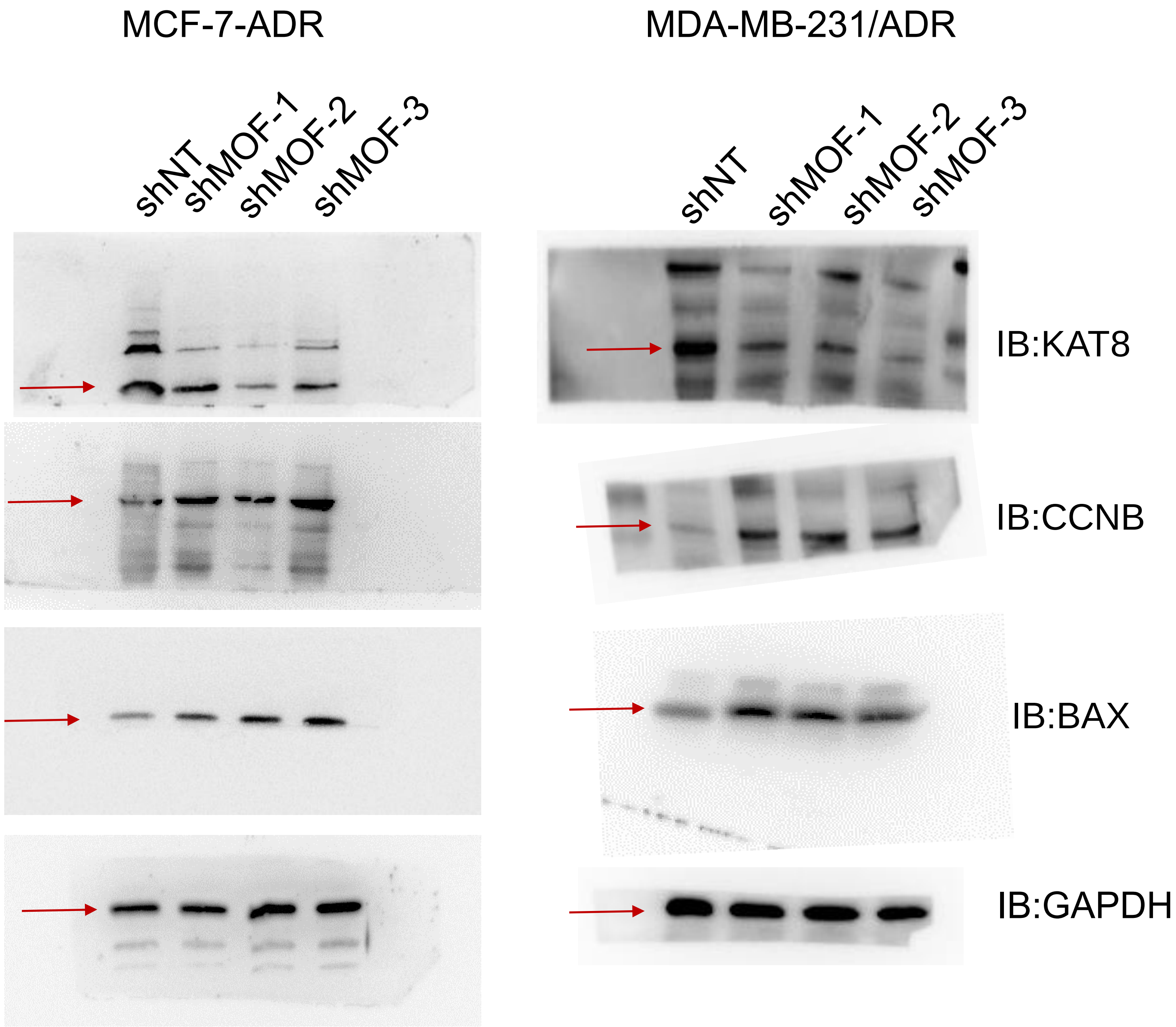
MCF-7-ADR
MDA-MB-231/ADR
shNT
shMOF-1
shMOF-2
shMOF-3
shNT
shMOF-1
shMOF-2
shMOF-3
IB:KAT8
IB:CCNB
IB:BAX
IB:GAPDH

Figure S3A

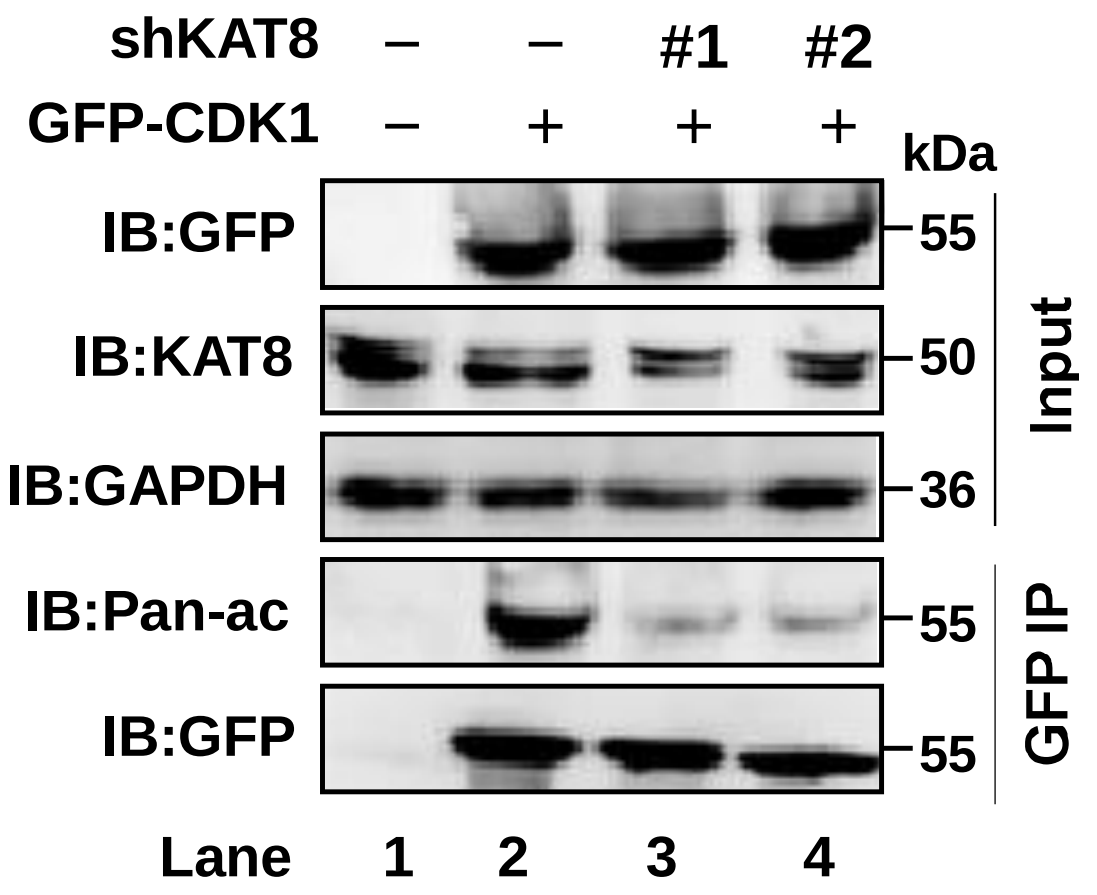

shKAT8 – – #1 #2
GFP-CDK1 – + + +
kDa
IB:GFP
55
IB:KAT8
50
IB:GAPDH
36
Input
IB:Pan-ac
55
IB:GFP
55
GFP IP
Lane 1 2 3 4

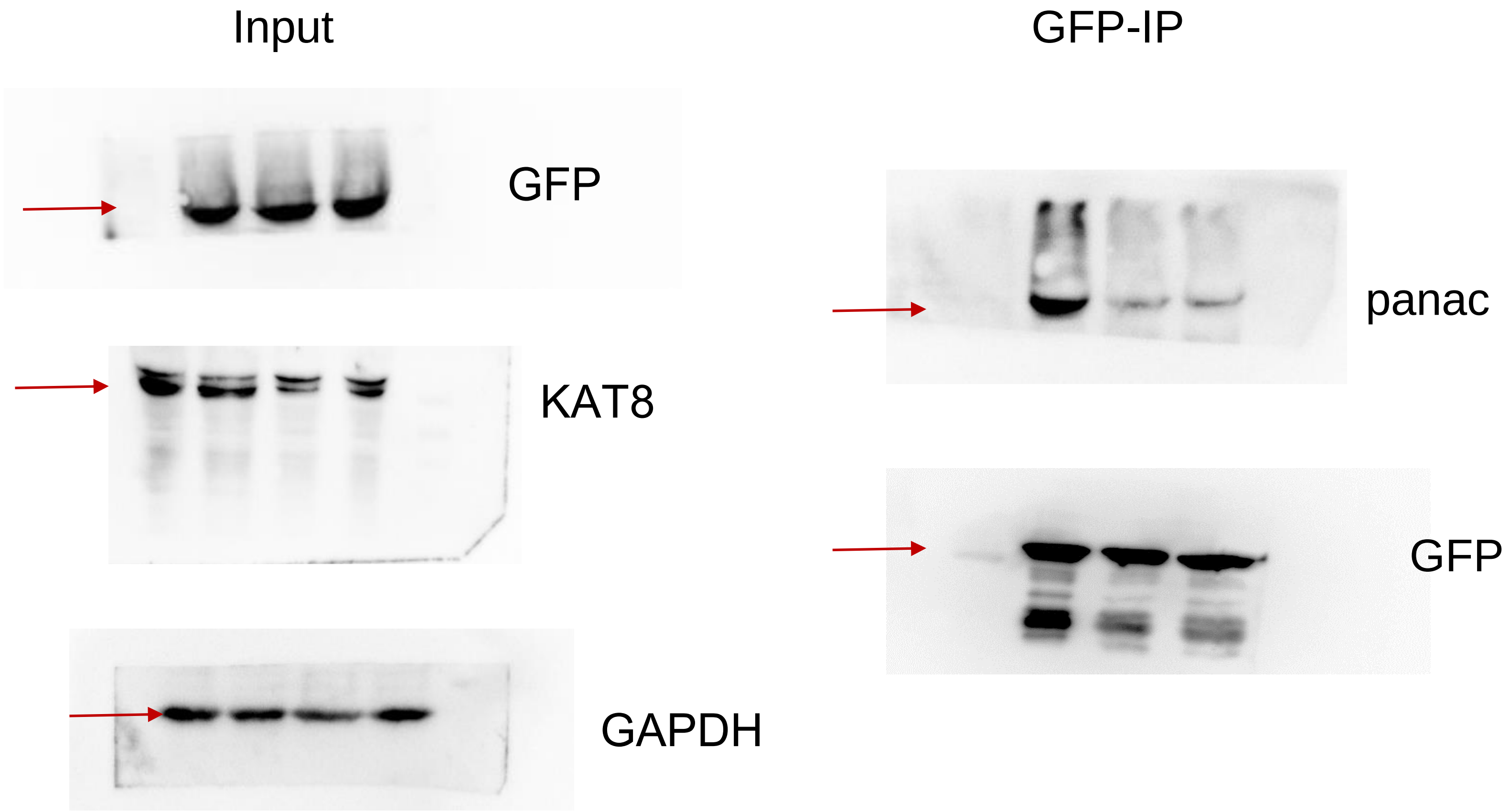

Input
GFP-IP
GFP
panac
KAT8
GFP
GAPDH

Figure S3B

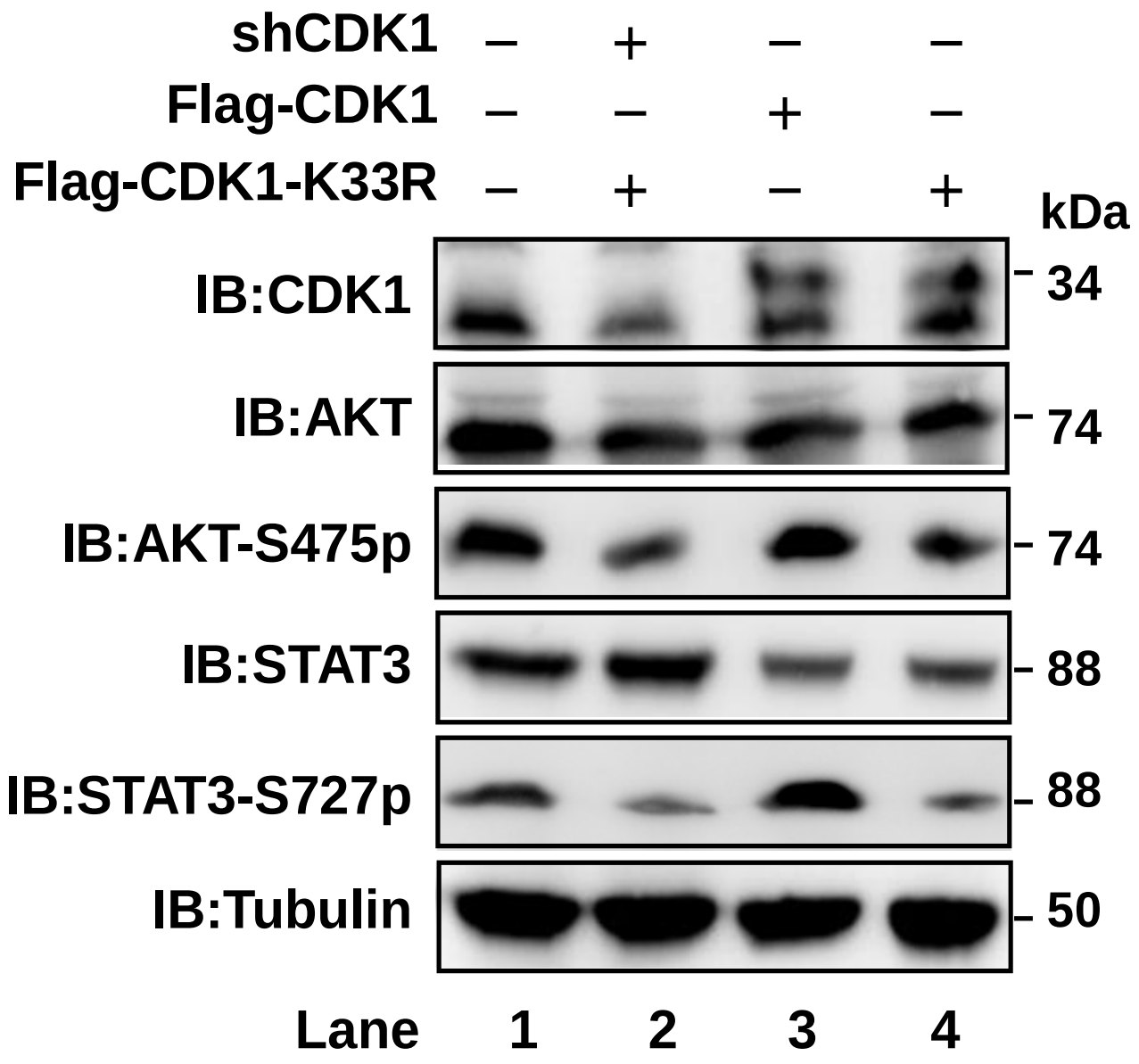
shCDK1 – + – –
Flag-CDK1 – – + –
Flag-CDK1-K33R – + – +
kDa
IB:CDK1
34
IB:AKT
74
IB:AKT-S475p
74
IB:STAT3
88
IB:STAT3-S727p
88
IB:Tubulin
50
Lane 1 2 3 4

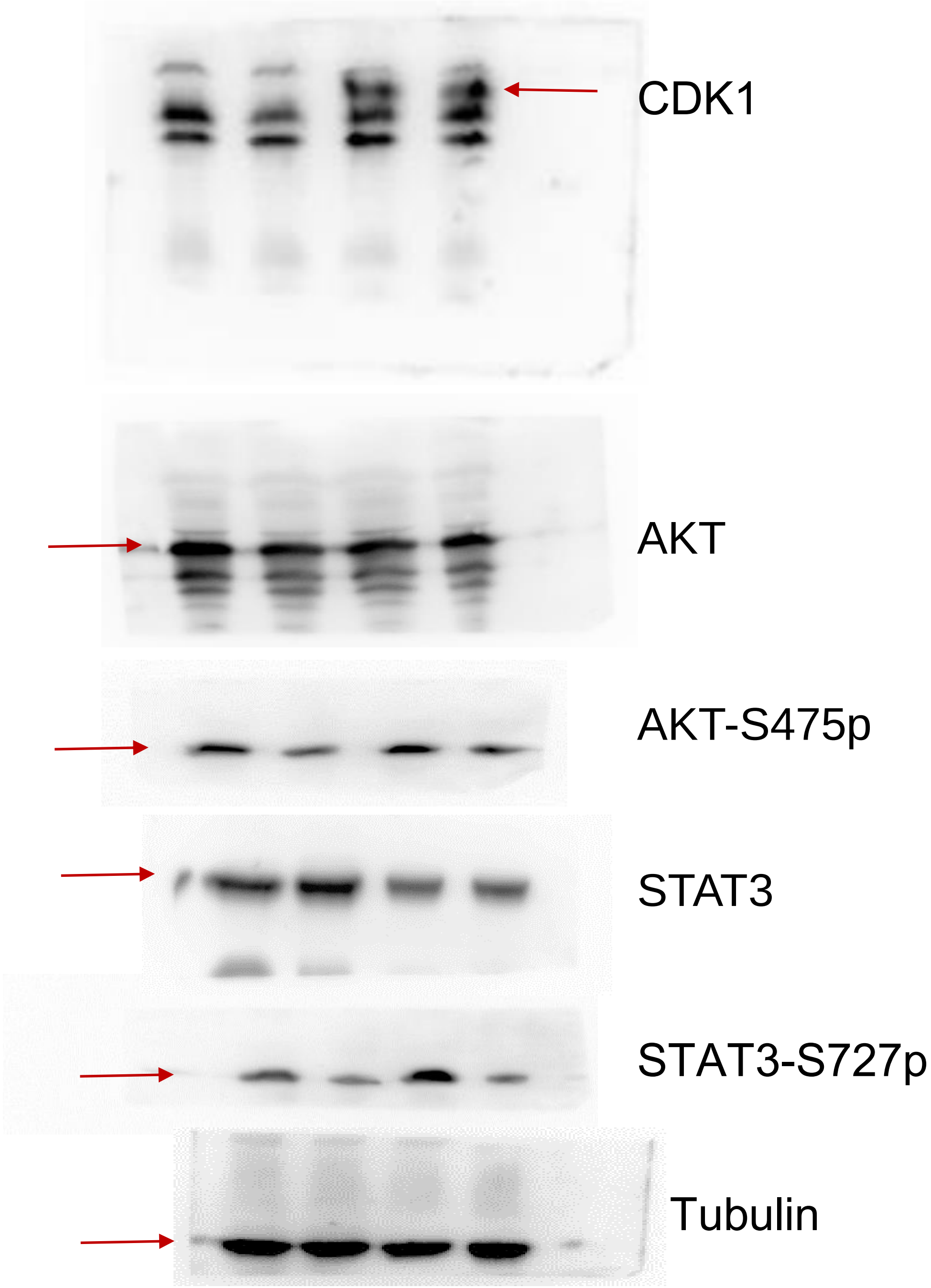
CDK1
AKT
AKT-S475p
STAT3
STAT3-S727p
Tubulin

Figure 3C

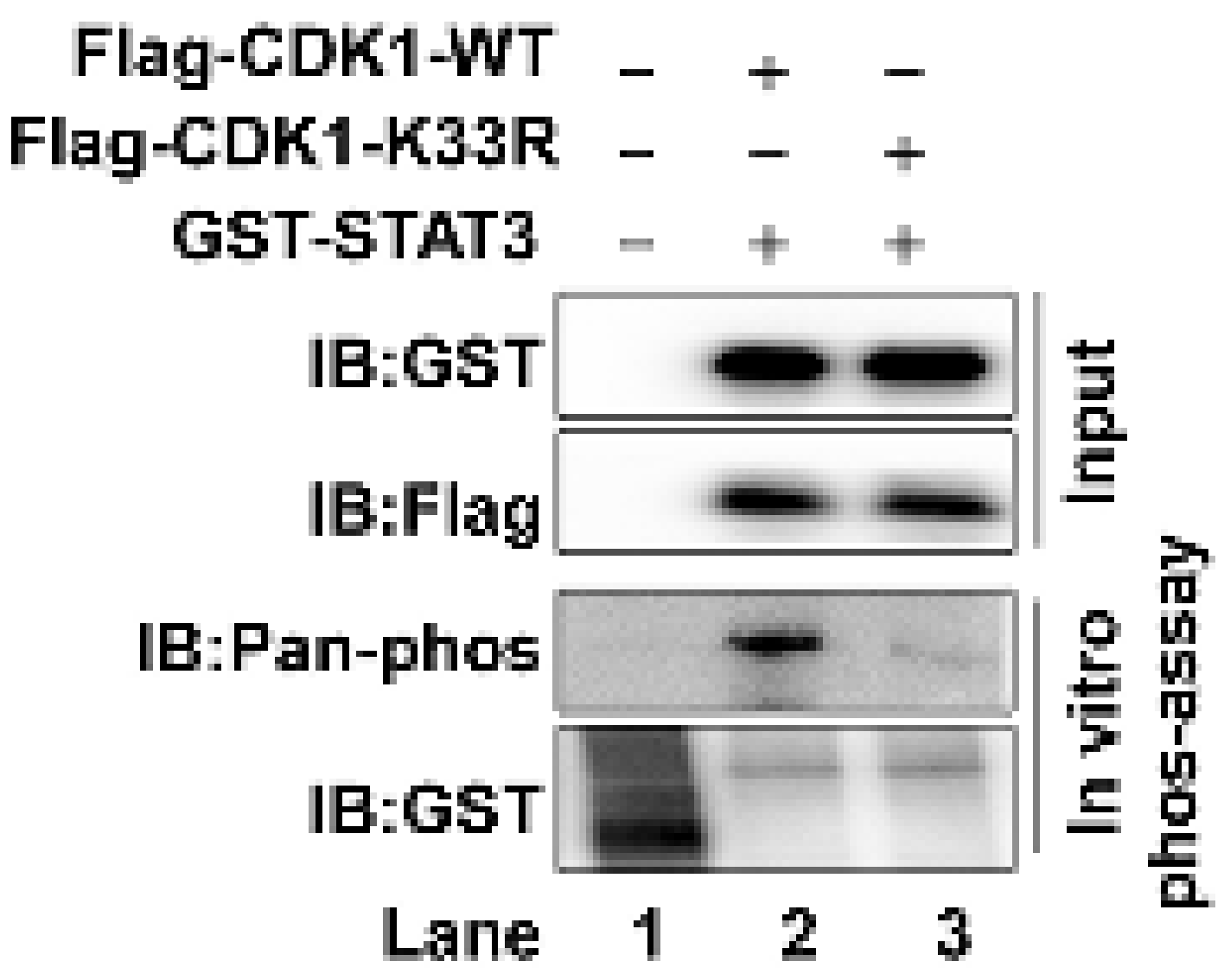

Flag-CDK1-WT
Flag-CDK1-K33R
GST-STAT3
IB:GST
IB:Flag
IB:Pan-phos
IB:GST
Input
In vitro phos-assay
Lane 1 2 3

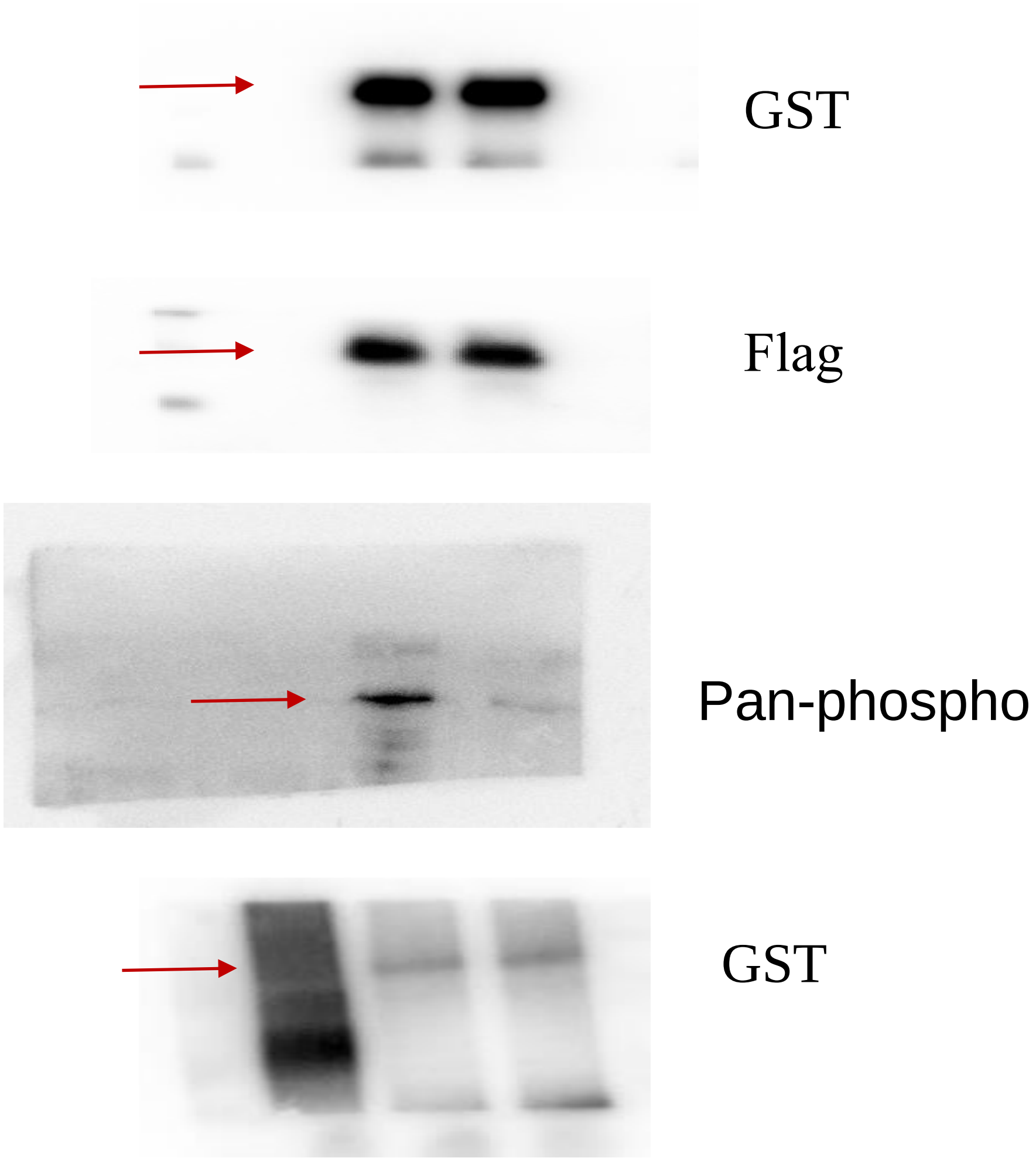

GST
Flag
Pan-phospho
GST

Figure S3D

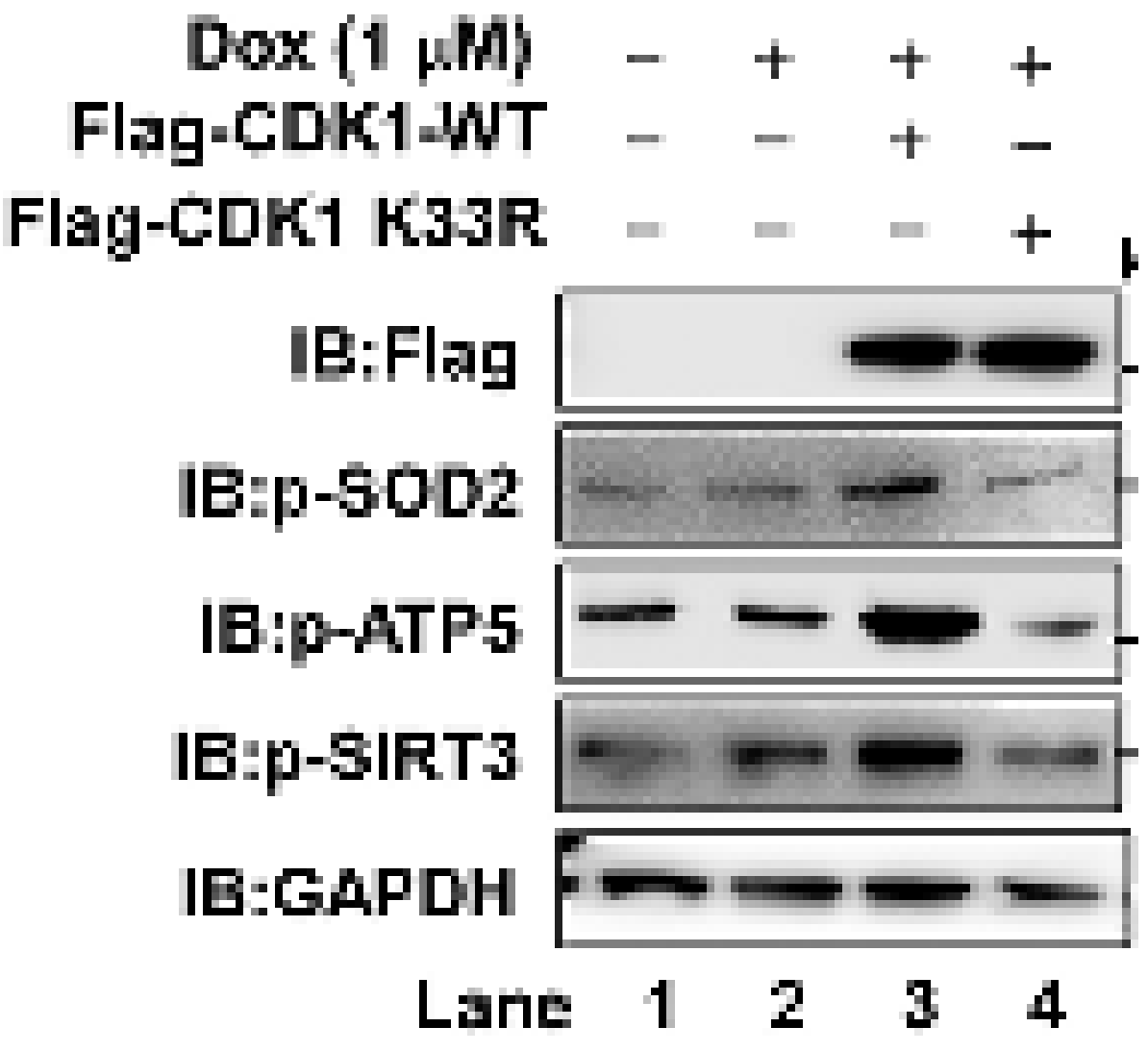
Dox (1 µM) − + + +
Flag-CDK1-WT − − + −
Flag-CDK1 K33R − − − +
IB:Flag
IB:p-SOD2
IB:p-ATP5
IB:p-SIRT3
IB:GAPDH
Lane 1 2 3 4

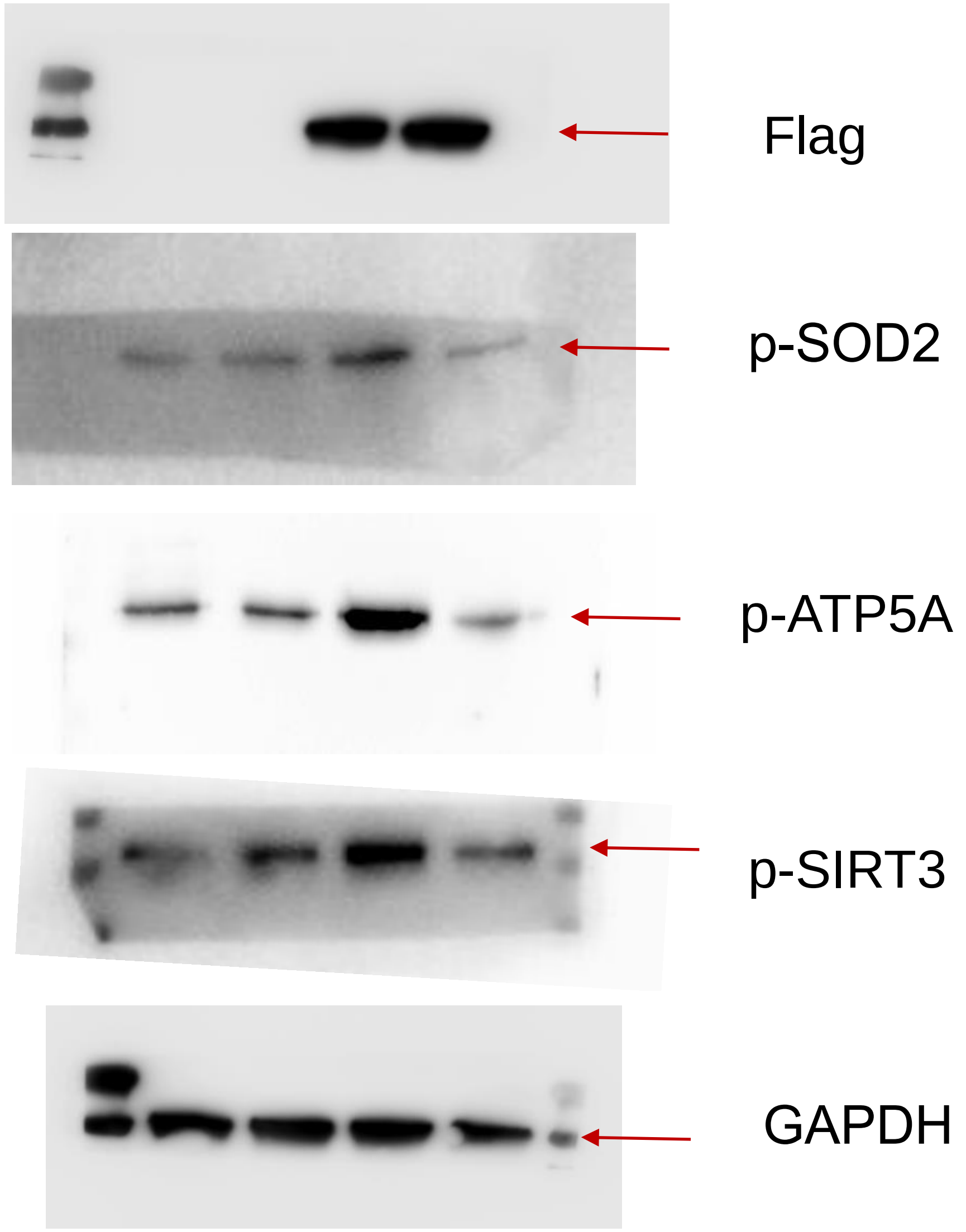
Flag
p-SOD2
p-ATP5A
p-SIRT3
GAPDH
