# Supplementary material for: The KAT8-CDK1 K33 acetylation axis drives doxorubicin resistance by suppressing ferroptosis and apoptosis in breast cancer
Source: Cell Death Dis. 2026 Aug 3;17(1):825. doi: 10.1038/s41419-026-09162-6 (PMC13601616; doi:10.1038/s41419-026-09162-6)
Supplement: Supplementary file 2 [file 41419_2026_9162_MOESM1_ESM.docx]

**Supplemental Methods**

**Antibodies and reagents**

The following antibodies were used in this study: Sigma-Aldrich (St. Louis, MO, USA): Anti-Flag (M2)- and anti-Myc-agarose, anti-Flag M2 (F3165) mouse monoclonal antibody; Epizyme Biotech (Shanghai, China): Anti-ACSL4 (R013285) and anti-P-gp (R013688) rabbit polyclonal antibodies; Servicebio (Wuhan, China): Anti-STAT3 (GB12176), anti-HDAC1 (GB112052-100) and anti-GFP (GB11602-100) rabbit polyclonal antibodies; Ruiying Biological (Suzhou, China): Anti-HA (RLM3003) mouse monoclonal antibody; Santa Cruz Biotechnology (Dallas, TX, USA): Anti-Myc (9E10) mouse monoclonal antibody; ABclonal Technology (Wuhan, China): Anti-CDK1 (AP0220), anti-KAT8 (A3390), anti-CCNB1 (A16038), anti-BAX (A12009), anti-CCNA (A2635), anti-phospho-CDK1-T161 (AP0324), anti-phospho-CDK1-Y15 (AP0233), anti-phospho-CDK1-T14 (AP015), and anti-Histone H4 (A23000) rabbit monoclonal antibodies; Jingjie Biotechnology (Hangzhou, China): Anti-pan-acetyl (PTM-105RM), anti-H4K16ac (PTM-122), and anti-CFL1 (PTM-5729) rabbit monoclonal antibodies; Boster Group (Wuhan, China): Anti-GST (BM3900) rabbit monoclonal antibody; Abmart (Shanghai, China): Anti-Tubulin (M2005), anti-AKT (PA1036), anti-pho-AKT(S473) (T10067), and anti-GPX4 (T56959) rabbit monoclonal antibodies; anti-GAPDH (NM_002046, full length) rabbit polyclonal antibody was raised against bacterially expressed proteins at Jilin University (Changchun, China).

The histone acetyltransferase (HAT) inhibitor MG149 (S7476) was obtained from Selleck Chemicals (Shanghai, China), TSA (M1753), Doxorubicin hydrochloride (M1969), MS-275 (M1791), and N-acetylcysteine (NAC, M5385) were provided by Abmole (Beijing, China). Nicotinamide (NAM, 65-82-7) was purchased from Aladin (Shanghai, China). Ferrostatin-1 (Fer-1, S7243), deferoxamine (DFO, S5742), chloroquine (CQ, S6999), Z-VAD-FMK (S7023), necrostatin-1 (Nec-1, S8037), and acetylcysteine (NAC, S1623) were sourced from Selleck Chemicals (Shanghai, China). The Lipid Peroxidation MDA Assay Kit (S0131S), and the GSH/GSSG Assay Kit (S0053) were obtained from Beyotime (Shanghai, China).

**Immunofluorescence staining**

MCF-7, MDA-MB-231, MCF-7/ADR, and MDA-MB-231/ADR cells were seeded on coverslips in 24-well plates and cultured to 70–80% confluence. Cells were fixed with 4% paraformaldehyde, permeabilized with 0.3% Triton X-100, and blocked with 1% BSA. After incubation with primary antibodies, cells were washed and incubated with appropriate secondary antibodies. Nuclei were counterstained with DAPI (Vector Laboratories). Fluorescence images were acquired using Olympus BX40F, Zeiss LSM700, or Nikon ECLIPSE Ti2 microscopes and analyzed with NIS-Elements or ZEN software.

**RNA extraction and qPCR**

Total RNA was extracted using a commercial kit and quantified with a Nanodrop spectrophotometer. One microgram of RNA was reverse-transcribed into cDNA. Quantitative PCR was performed with SYBR Green master mix on a real-time PCR system. Relative gene expression was calculated using the 2^−ΔCt method and normalized to an internal reference gene.

**Plasmids, gene silencing and transient transfection**

Full-length cDNAs encoding human KAT8 (NM_032188), CDK1 (NM_001786.5), WEE1 (NM_003390.4), MYT1 (NM_004535.3), HDAC1 (NM_004964.3), and point mutants including CDK1 (K33R) and KAT8 (K274R), were subcloned into pcDNA3.1(–) and pLVX-puro vectors, with GFP, Flag, or Myc tags. Plasmids were transiently transfected into cells using polyethyleneimine (PEI) (23966, PolySciences, Beijing, China) according to the manufacturer’s protocol. KAT8 and CDK1 knockdown, KAT8 depletion/knockout validation, and GPX4, ACSL4 or BAX rescue/silencing experiments were performed using lentiviral or plasmid-based systems as previously described with minor modifications [1].

**shRNA knockdown**

The pLVX-shRNA system was used to express shRNA in MCF-7/ADR or MDA-MB-231/ADR cells. The target sequences were as follows: KAT8-specific shRNAs: shKAT8-1, CGAAATTGATGCCTGGTAT; shKAT8-2, GTGATCCAGTCTCGAGTGA; CDK1-specific shRNA: shCDK1-3’UTR-1, GTGGAATCTTTACAGGACTAT; shACSL4 target: 5'-GCAGTAGTTCATGGGCTAAAT-3', shBAX-1 target: 5'-GCCGGAACTGATCAGAACCAT-3'; KO-KAT8-1 guide: 5'-ACAGGTCCGCACGGAAAACC-3', KO-KAT8-2 guide: 5'-GGACGGCCACAGAAGCCAAT-3'.

**Transient transfection and immunoprecipitation (IP)**

HEK293T and MCF-7/ADR cells were transiently transfected with the indicated plasmids. Forty-eight hours later, cells were lysed in RIPA buffer containing protease inhibitors. Lysates were incubated with the appropriate antibody-conjugated beads, and immunoprecipitated proteins were eluted in SDS sample buffer for analysis by Western blot.

**Recombinant protein expression and GST pull-down**

Full-length and mutant CDK1, and full-length KAT8, were cloned into pGEX-6p-1 and expressed as GST fusion proteins in E. coli. Purified GST or GST-KAT8 proteins were incubated with cell lysates, and protein complexes were captured with glutathione-Sepharose beads. After washing, bound proteins were eluted in SDS sample buffer and analyzed by Western blot.

**In vitro KAT assay**

Purified GST-CDK1, GST-CDK1-K33R, and Myc-KAT8 proteins were incubated with acetyl-CoA in HAT assay buffer. Reactions were terminated by heating in SDS sample buffer, and acetylation was analyzed by Western blot.

**Cell viability and oxidative stress assays**

Cell viability was measured in MCF-7, MCF-7/ADR, MDA-MB-231, and MDA-MB-231/ADR cells using the CCK-8 assay. Intracellular ROS levels were assessed with the DCFH-DA probe by fluorescence imaging. Reduced (GSH) and oxidized (GSSG) glutathione levels were determined using a GSH/GSSG assay kit. Lipid peroxidation was quantified by measuring malondialdehyde (MDA) in cell lysates using an MDA assay kit, with results normalized to total protein.

**Mitochondrial membrane potential assay**

ΔΨm was assessed using JC-1 dye. Cells were incubated with the dye, washed, and fluorescence was imaged to evaluate changes in mitochondrial membrane potential.

**Lipid peroxidation assay (C11-BODIPY staining)**

Cells were pretreated as indicated, incubated with C11-BODIPY, fixed, and fluorescence imaging was performed to assess lipid peroxidation.

**Colony formation and organoid assays**

Cells were seeded at low density for colony formation, treated as indicated, cultured to allow colony growth, then fixed, stained, and counted. Colonies with >20 cells were scored as positive. For organoid formation, cells were embedded in Matrigel, cultured in complete medium, and observed by fluorescence microscopy. Organoid diameter and formation efficiency were quantified. Experiments were performed in triplicate.

**Apoptosis assay**

MCF-7, MDA-MB-231, MCF-7/ADR and MDA-MB-231/ADR cells were treated as indicated, harvested, and stained with Annexin V-FITC/PI. Apoptosis and necrosis were analyzed by flow cytometry.

**AO/PI cell viability assay**

Cells were stained with acridine orange (AO) and propidium iodide (PI) and analyzed by fluorescence microscopy or a cell counter to distinguish live and dead cells.

**Cellular thermal shift assay (CETSA)**

The thermal stability of P-glycoprotein (P-gp) was assessed using CETSA. Treated cells were lysed, subjected to a temperature gradient, and centrifuged to remove aggregated proteins. Soluble proteins were collected for subsequent analysis.

**P-gp functional assay**

The activity of P-glycoprotein (P-gp) was assessed by measuring intracellular accumulation of Rhodamine 123. Treated cells were incubated with the fluorescent substrate, washed, and fluorescence intensity was measured to evaluate P-gp function.

**Immunohistochemistry and in vivo experiments**

Protein expression in tumor samples was evaluated by immunohistochemistry for Ki-67, GPX4, BAX, KAT8, and H4K16ac. For xenograft studies, stably transfected or control MCF-7/ADR cells were subcutaneously injected into nude mice. When tumors reached 80–100 mm³, mice received intraperitoneal injections of saline, doxorubicin, or MG149 at indicated time points. Tumor growth was monitored, and tissues were collected for downstream analyses. All animal experiments were approved by the Institutional Animal Care and Use Committee of Jilin University.

**Reference**

1. Chu J, Zhao Q, Wang Y, Cai C, Cui X, Zhang N, et al. Ginsenoside Rg5 targets the KAT8-CISD2 axis to maintain mitochondrial homeostasis and antagonize senescence. Phytomedicine 2026, **153:** 157923.

**Supplemental Figures**

**Figures S1**

**Fig. S1** KAT8 depletion impairs proliferation, mitochondrial function, and Dox resistance in breast cancer cells. **A** Correlation analysis between KAT8 and H4K16ac expression based on Pearson’s correlation coefficient. **B** EdU incorporation analysis showing the proportion of EdU-positive cells in MCF-7/ADR and MDA-MB-231/ADR cells following transfection with non-targeting shRNA (shNT) or two independent KAT8 shRNAs. **C** Quantification of organoid diameters in shNT- and shKAT8-expressing MCF-7/ADR and MDA-MB-231/ADR cells. **D** JC-1 staining showing changes in mitochondrial membrane potential in MCF-7/ADR and MDA-MB-231/ADR cells treated with DMSO, Dox, MG149, or Dox plus MG149. The red/green fluorescence ratio indicates relative mitochondrial membrane potential. Scale bars, 50 μm. **E** Statistical analysis of organoid diameters following treatment with DMSO, Dox, MG149, or combined Dox and MG149. **F** Cell viability curves and IC_50_ values for Dox in vector-control, MG149-treated, KAT8-knockout, and KAT8-knockout plus MG149-treated MCF-7/ADR and MDA-MB-231/ADR cells. Data are presented as mean ± SD; ns, not significant; **P < 0.01, ***P < 0.001, ****P < 0.0001.

**Figures S2**

**Fig. S2** KAT8 depletion suppresses cell-cycle progression and enhances Dox sensitivity through CDK1 regulation. **A** Western blot analysis of KAT8, CCNB1 and BAX expression in MCF-7/ADR and MDA-MB-231/ADR cells expressing control shRNA or independent KAT8 shRNAs. GAPDH was used as a loading control. **B** Colony formation analysis of MCF-7/ADR and MDA-MB-231/ADR cells treated with DMSO or Dox after expression of vector, shKAT8, or combined shKAT8 and shCDK1. **C** Colony formation analysis of MCF-7/ADR and MDA-MB-231/ADR cells treated with DMSO or Dox after KAT8 knockdown with or without re-expression of Flag-CDK1. Data are presented as mean ± SD; ns, not significant; *P < 0.05, **P < 0.01, ***P < 0.001, ****P < 0.0001.

**Figures S3**

**Fig. S3** KAT8-dependent CDK1 K33 acetylation regulates kinase activity, DNA damage reapir, mitochondrial signaling, and cell-cycle progression. **A** Immunoprecipitation of GFP-CDK1 followed by immunoblotting with an anti-pan-acetyl-lysine antibody showing that KAT8 knockdown decreases CDK1 acetylation. **B** Western blot analysis of CDK1, phospho-AKT (S473), total AKT, STAT3 and phospho-STAT3 (S727) after endogenous CDK1 knockdown and rescue with Flag-CDK1-WT or Flag-CDK1-K33R. Tubulin served as a loading control. **C** In vitro kinase assay using GST-STAT3 as substrate and Flag-CDK1 WT or K33R, followed by immunoblotting with anti-pan-phospho-threonine/serine antibody. **D** Western blot analysis of phospho-SOD2, phospho-ATP5 and phospho-SIRT3 in cells treated with Dox and rescued with Flag-CDK1 WT or K33R. **E, F** Representative γ-H2AX immunofluorescence images and quantification of γ-H2AX-positive cells in MCF-7/ADR, MDA-MB-231/ADR and MCF-7 cells under the indicated treatment conditions. **G, H** Flow-cytometric analysis and quantification of cell-cycle distribution MCF-7/ADR and MDA-MB-231/ADR cells, showing the proportions of cell in G0/G1, S and G2/M phases. Data are presented as mean ± SD; ns, not significant; **P < 0.01, ***P < 0.001, ****P < 0.0001.

**Figures S4**

**Fig. S4** Ferroptosis and apoptosis contributes to cell death induced by disruption of the KAT8-CDK1 axis. **A** Cell viability of MCF-7/ADR and MDA-MB-231/ADR cells treated with DMSO, Dox, Dox plus CDK1 K33R, or CDK1 K33R in combination with the ferroptosis inhibitor Fer-1 and/or the pan-caspase inhibitor Z-VAD-FMK. **B** C11-BODIPY staining showing lipid peroxidation in MCF-7/ADR and MDA-MB-231/ADR cells treated with Dox plus shKAT8 and CDK1-K33R, with additional GPX4/ACSL4 overexpression or GPX4/ACSL4 knockdown, as indicated. The oxidized/unoxidized fluorescence ratio indicates relative lipid peroxidation. Scale bars, 50 μm. **C, D** Representative Annexin V-FITC/PI flow-cytometry plots and quantification of early and late apoptotic cells in MCF-7/ADR and MDA-MB-231/ADR cells following Dox treatment, KAT8 knockdown, rescue with CDK1 WT or K33R, and BAX knockdown. Data are presented as mean ± SD; ***P < 0.001.

**Figures S5**

**Fig. S5** Disruption of the KAT8-CDK1 axis enhances oxidative stress and lipid peroxidation in Dox-treated breast cancer cells. **A, B** Representative DCFH-DA fluorescence images and quantification of intracellular ROS levels in MCF-7, MCF-7/ADR, MDA-MB-231 and MDA-MB-231/ADR cells treated with Dox in combination with shKAT8 and Flag-CDK1 WT or CDK1-K33R, as indicated. Scale bars, 100 μm. **C** Relative intracellular reduced glutathione (GSH) levels in the indicated treatment groups. **D** Relative malondialdehyde (MDA) levels, indicating lipid peroxidation, in the indicated cells and treatment groups. **E** Representative C11-BODIPY staining images showing oxidized and unoxidized lipid signals in parental and Dox-resistant breast cancer cells under the indicated conditions. Scale bars, 50 μm. Data are presented as mean ± SD; ns, not significant; *P < 0.05, **P < 0.01, ***P < 0.001.

**Table S1 Relationship between KAT8 expression (qPCR) and IHC parameters of breast cancer.**

| Factor | Case  (n) | No Dox  Mean ± SEM  (2^-ΔΔCT^ values) | Dox  Mean ± SEM  (2^-ΔΔCT^ values) | *P*-value  No Dox vs. Dox | Significant difference |
| --- | --- | --- | --- | --- | --- |
| Ki-67 IHC (%)  ≤20  >20%  ER IHC (%)  Negative  Positive  HER-2 IHC (%)  Negative  Positive  PR IHC (%)  Negative  Positive | 48  47  36  59  37  58  55  40 | 0.002041629 ± 0.00058  0.001177844 ± 0.00031  0.001052003 ± 0.00048  0.0016073 ± 0.00030  0.002209043±0.00046  0.000685871 ± 0.00023  0.001682659 ± 0.00085  0.001285084 ± 0.00025 | 0.03235892 ± 0.01011  0.038278601 ± 0.01867  0.019710026 ± 0.00768  0.04183013 ± 0.01299  0.048901828 ± 0.01267  0.003944864 ± 0.00149  0.01829891 ± 0.00581  0.050666878 ± 0.01684 | 0.618  0.132  0.999  0.022  0.007  0.999  0.999  0.002 | NS  NS  NS  *P* < 0.05  *P* < 0.01  NS  NS  *P* < 0.01 |

IHC, immunohistochemistry; Ki-67, marker of proliferation Ki-67; ER, estrogen receptor; PR, progesterone receptor; HER-2, human epidermal growth factor receptor 2; NS, no significant difference. All qPCRs were performed in three independent experiments with three replicates per group. Statistical differences between two groups were analyzed using the Mann Whitney test.
